# Supplementary material for: Anticipating regime shifts by mixing early warning signals from different nodes
Source: Nat Commun. 2024 Feb 5;15:1086. doi: 10.1038/s41467-024-45476-9 (PMC10844243; doi:10.1038/s41467-024-45476-9)
Supplement: Supplementary file 1 — Supplementary Information [file 41467_2024_45476_MOESM1_ESM.pdf]

Supplementary Information for: Anticipating regime shifts by mixing early warning signals from different nodes

Naoki Masuda, Kazuyuki Aihara, Neil G. MacLaren

Supplementary Note 1: Derivation of var$[\hat{V}_S]$

We obtain the unbiased sample covariance of $x_i(t)$ calculated from L samples as follows:

$$
\begin{aligned}
\hat{V}_i =& \frac{1}{L-1} \sum_{\ell=1}^{L} \left(x_{i,\ell} - \frac{1}{L} \sum_{\ell'=1}^{L} x_{i,\ell'} \right)^2 \\
=& \frac{1}{L-1} \sum_{\ell=1}^{L} \left(z_{i,\ell} - \frac{1}{L} \sum_{\ell'=1}^{L} z_{i,\ell'} \right)^2 \\
=& \frac{1}{L-1} \left[\sum_{\ell=1}^{L} z_{i,\ell}^2 - \frac{1}{L} \left(\sum_{\ell'=1}^{L} z_{i,\ell'} \right)^2 \right],
\end{aligned}
\tag{S1}
$$

where $x_{i,\ell}$ (with $i \in \{1, \ldots, N\}$ and $\ell \in \{1, \ldots, L\}$) is the ℓth sample of $x_i(t)$ in the equilibrium, and $z_{i,\ell} = x_{i,\ell} - x_i^*$. We obtain

$$
E[\hat{V}_i] = C_{ii}, \tag{S2}
$$

$$
E\left[\left(\hat{V}_i \right)^2 \right] = \frac{(L-1)E[z_i^4] + (L^2 - 2L + 3)E[z_i^2]}{L(L-1)}, \tag{S3}
$$

where E denotes the expectation [1–3].

For $\hat{V}_S = \sum_{i=1}^n \hat{V}_i/n$, we similarly obtain

$$
\begin{aligned}
E\left[\left(\hat{V}_S\right)^2\right] =& \frac{1}{n^2}\sum_{i=1}^n\sum_{j=1}^n \frac{L\left(\sum_{\ell=1}^L z_{i,\ell}^2\right)^2 - \left(\sum_{\ell'=1}^L z_{i,\ell'}\right)^2}{L(L-1)} \cdot \frac{L\left(\sum_{\ell=1}^L z_{j,\ell}^2\right)^2 - \left(\sum_{\ell'=1}^L z_{j,\ell'}\right)^2}{L(L-1)} \\
=& \frac{1}{n^2 L^2 (L-1)^2}\left\{L^2 \sum_{i=1}^n\sum_{j=1}^n E\left[\left(\sum_{\ell=1}^L z_{i,\ell}^2\right)\left(\sum_{\ell'=1}^L z_{j,\ell'}^2\right)\right] - 2L\sum_{i=1}^n\sum_{j=1}^n E\left[\left(\sum_{\ell=1}^L z_{i,\ell}^2\right)\left(\sum_{\ell'=1}^L z_{j,\ell'}\right)^2\right]\right. \\
&\left. + \sum_{i=1}^n\sum_{j=1}^n E\left[\left(\sum_{\ell=1}^L z_{i,\ell}\right)^2\left(\sum_{\ell'=1}^L z_{j,\ell'}\right)^2\right]\right\} \\
=& \frac{1}{n^2 L^2 (L-1)^2}\left\{L^2 \sum_{i=1}^n\sum_{j=1}^n \left(LE[z_i^2 z_j^2] + L(L-1)E[z_i^2]E[z_j^2]\right)\right. \\
& - 2L\sum_{i=1}^n\sum_{j=1}^n \left(LE[z_i^2 z_j^2] + L(L-1)E[z_i^2]E[z_j^2]\right) \\
&\left. + \sum_{i=1}^n\sum_{j=1}^n \left(LE[z_i^2 z_j^2] + \frac{L(L-1)}{2}\cdot 2E[z_i^2]E[z_j^2] + \frac{L(L-1)}{2}\cdot 4E[z_i z_j]^2\right)\right\} \\
=& \frac{1}{n^2 L(L-1)}\sum_{i=1}^n\sum_{j=1}^n \left\{(L-1)E[z_i^2 z_j^2] + (L-1)^2 C_{ii}C_{jj} + 2(C_{ij})^2\right\}. \qquad \text{(S4)}
\end{aligned}
$$

In Eq. (S4), we have omitted subscript ℓ from $z_{i,\ell}$ to denote it by z_i when it is an arbitrary sample and does not cause confusion.

Using Eq. (5) in the main text and Eq. (S4), we obtain the variance of $\hat{V}_S$, denoted by $\mathrm{var}[\hat{V}_S]$, as follows:

$$
\begin{aligned}
\mathrm{var}[\hat{V}_S] =& E\left[\left(\hat{V}_S\right)^2\right] - \left(E[\hat{V}_S]\right)^2 \\
=& \frac{1}{n^2 L}\left(\sum_{i=1}^n\sum_{j=1}^n E[z_i^2 z_j^2] - \sum_{i=1}^n\sum_{j=1}^n C_{ii}C_{jj} + \frac{1}{L-1}\sum_{i=1}^n\sum_{j=1}^n (C_{ij})^2\right). \qquad \text{(S5)}
\end{aligned}
$$

Because the stationary distribution of the multivariate OU process is a multivariate Gaussian distribution [4, 5], we obtain $E[z_i^2 z_j^2] = C_{ii}C_{jj} + 2(C_{ij})^2$ including the case of $i = j$. By substituting this relationship in Eq. (S5), we obtain

$$
\begin{aligned}
\mathrm{var}[\hat{V}_S] =& \frac{2}{n^2(L-1)}\sum_{i=1}^n\sum_{j=1}^n (C_{ij})^2 \\
=& \frac{2}{n^2(L-1)}\mathrm{Tr}\left(\overline{C}^2\right) \\
=& \frac{2}{n^2(L-1)}\sum_{i=1}^n \lambda_i^2. \qquad \text{(S6)}
\end{aligned}
$$

Supplementary Note 2: Performance of the maximizer of d when different pairs of bifurcation parameter values are used for calculating d

A Networks with two or three nodes

For the network with two nodes connected by a directed edge and the chain network with three nodes, we used $r = -0.3$ and -0.1 in the main text to compute covariance matrices $C^{(1)}$ and $C^{(2)}$ and then d. In this section, we evaluate the robustness of the maximizer of d with respect to the choice of the two r values. For each pair of r values drawn from the entire range of r that we used in the main text, we computed d for all the possible node sets.

First, we consider the two-node network. For each set of the parameter values used in Fig. 3, we identify the node set among the three candidates, i.e., $S = \{1\}$, $\{2\}$, and $\{1, 2\}$, that maximizes d. We show the S maximizing d for a range of the two r values in Figs. S1(a), (b), and (c), which correspond to the parameter values used in Figs. 3(a), (b), and (c), respectively. We showed in the main text that d is the largest with $S = \{1, 2\}$ for the parameter values used in Figs. S1(a) and (b), and with $S = \{1\}$ for those used in Fig. S1(c) when the two r values are -0.3 and -0.1. Figures S1(a), (b), and (c) indicate that the node set maximizing d remains the same for all (see Fig. S1(a)) or almost all (see Figs. S1(b) and (c)) the pairs of r values.

We carried out the same analysis for the chain network with three nodes. We show the node set maximizing d for each pair of r values in Figs. S1(d), (e), and (f), which correspond to the parameter values used in Figs. 4(a), (b), and (c), respectively. We showed in the main text that d is the largest with $S = \{1, 2, 3\}$, $S = \{2\}$, and $S = \{1, 3\}$ for the parameter values used in Figs. S1(a), (b), and (c), respectively, when the two r values are -0.3 and -0.1. We find that the node set maximizing d remains the same for all the pairs of r values in Figs. S1(d) and (f). In Fig. S1(e), $S = \{2\}$ maximizes d only when the two r values are large. In other regions of the two r values, the node set maximizing d is $S = \{1, 2, 3\}$, which realizes the second largest d value when the two r values are -0.3 and -0.1.

Putting these results together, we conclude that the maximizer of d is fairly robust for these two- and three-node networks against the variation in the r value. The only exception is Fig. S1(e). However, even in this case, $S = \{2\}$, which we highlighted in the main text, is the maximizer of d in a reasonably large region of the two r values.

B Larger networks

For expository purposes, let us assume that the bifurcation parameter is $u = \overline{u}_k$, $k \in \{1, 2, \ldots, \tilde{K}\}$ such that $\overline{u}_{\tilde{K}}$ is the u value closest to the first tipping point. In the main text, we computed covariance matrices $C^{(1)}$ and $C^{(2)}$ at $\overline{u}_k$ with $k = k^{(1)} = \text{round}(0.1\tilde{K})$ and $k = k^{(2)} = \text{round}(0.9\tilde{K})$, respectively, where round() represents rounding to the closest integer. Then, we calculated d for each node set S. In the remainder of this section, we omit round() to simplify the notation. In this section, we evaluate the robustness of the performance of the maximizer of d with respect to the choice of $k^{(1)}$ and $k^{(2)}$. Specifically, we calculate p_1 and p_2 with $(k^{(1)}, k^{(2)}) = (0.1\tilde{K}, 0.3\tilde{K})$, $(0.1\tilde{K}, 0.5\tilde{K})$, $(0.1\tilde{K}, 0.7\tilde{K})$, $(0.3\tilde{K}, 0.5\tilde{K})$, $(0.3\tilde{K}, 0.7\tilde{K})$, $(0.3\tilde{K}, 0.9\tilde{K})$, $(0.5\tilde{K}, 0.7\tilde{K})$, $(0.5\tilde{K}, 0.9\tilde{K})$, and $(0.7\tilde{K}, 0.9\tilde{K})$, and for each scenario for u_i and σ_i (i.e., constant across all nodes or heterogeneous), network, dynamical system, and bifurcation parameter (i.e., u or D).

We show the results for the BA and Chesapeake Bay networks in Figs. S2–S10, those for the Erdős-Rényi and node fitness networks in Figs. S11–S19, and those for the freshwater stream food web and dolphin social networks in Figs. S20–S28. Except for the gene regulatory dynamics, for which the maximizer of d performs relatively poorly even with the original $k^{(1)}$ and $k^{(2)}$ values (see Figs. 8 and S31), p_1 and p_2 are always less than 1 if $(k^{(1)}, k^{(2)}) = (0.1\tilde{K}, 0.7\tilde{K})$, $(0.3\tilde{K}, 0.9\tilde{K})$, $(0.5\tilde{K}, 0.9\tilde{K})$, or $(0.7\tilde{K}, 0.9\tilde{K})$, only with a single exception (Fig. S19(e)). The p_1 and p_2 values for these $(k^{(1)}, k^{(2)})$ pairs are substantially

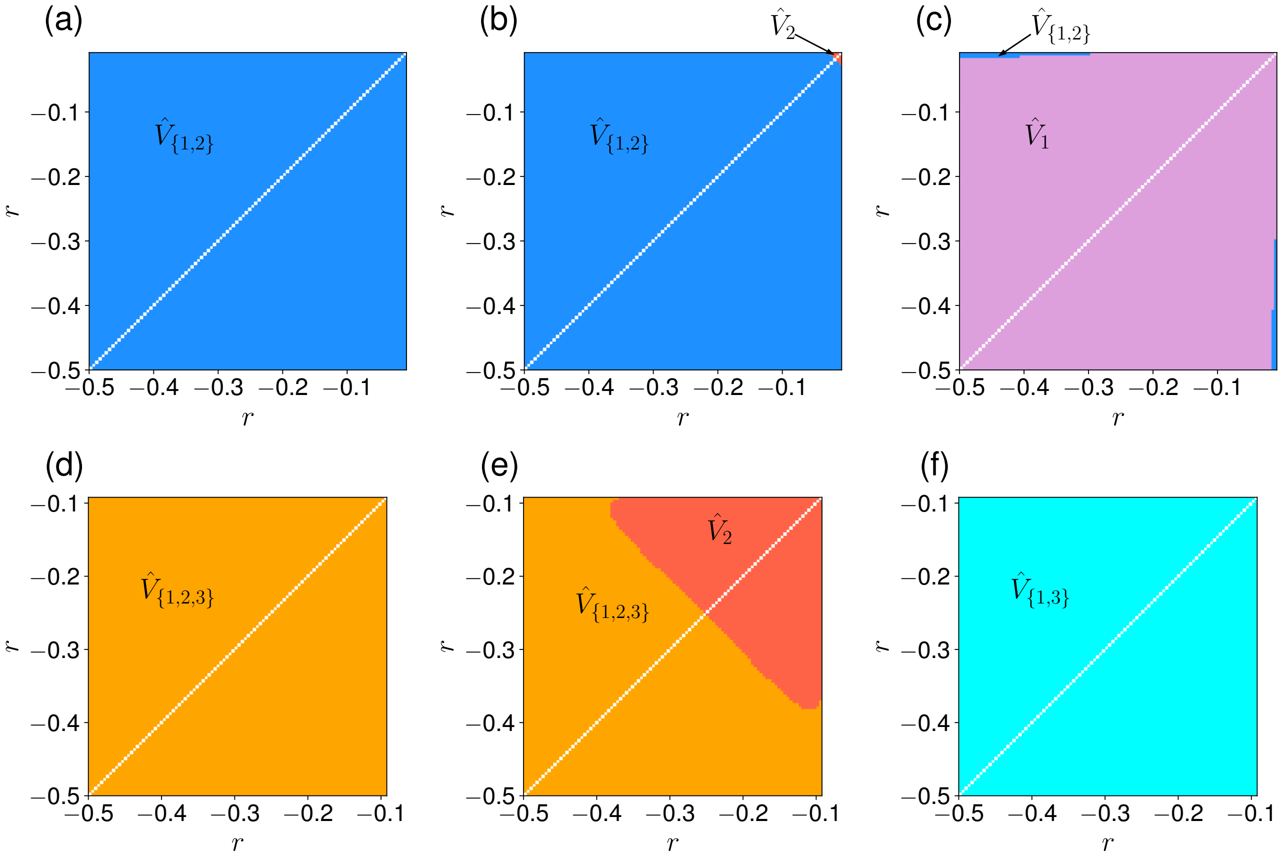

Figure S1: Robustness of the optimized node set for small networks. In each panel, the horizontal and vertical axes show the two values of r. Each color represents the node set that maximizes d. (a) $N = 2$ and $(\sigma_2, \Delta r) = (0.1, 1)$. (b) $N = 2$ and $(\sigma_2, \Delta r) = (0.1, 0.5)$. (c) $N = 2$ and $(\sigma_2, \Delta r) = (0.2, 1)$. (d) $N = 3$ and $\sigma_1 = 0.1$. (e) $N = 3$ and $\sigma_1 = 0.7$. (f) $N = 100$ and $\sigma_1 = 0.015$. We set $w = 0.5$ and $\sigma_1 = 0.1$ for the network with $N = 2$ nodes connected by a directed edge (i.e., (a)–(c)). We set $w = 0.05$ and $\sigma_2 = 0.1$ for the chain network with $N = 3$ nodes (i.e., (d)–(f)). We set $L = 100$ for both networks. All the other parameter values used are the same as those used in Figs. 3 and 4 in the main text. Because one needs to use two different r values to calculate d, the diagonals are shown in white.

smaller than 1 in most cases. These results indicate that the performance of the maximizer of d is robust with respect to the choice of $k^{(1)}$ and $k^{(2)}$ as long as the two bifurcation parameter values are reasonably far from each other (i.e., $(k^{(1)}, k^{(2)}) = (0.1\tilde{K}, 0.7\tilde{K})$ or $(0.3\tilde{K}, 0.9\tilde{K})$) or one of them is close to the tipping point (i.e., $(k^{(1)}, k^{(2)}) = (0.3\tilde{K}, 0.9\tilde{K})$, $(0.5\tilde{K}, 0.9\tilde{K})$, or $(0.7\tilde{K}, 0.9\tilde{K})$).

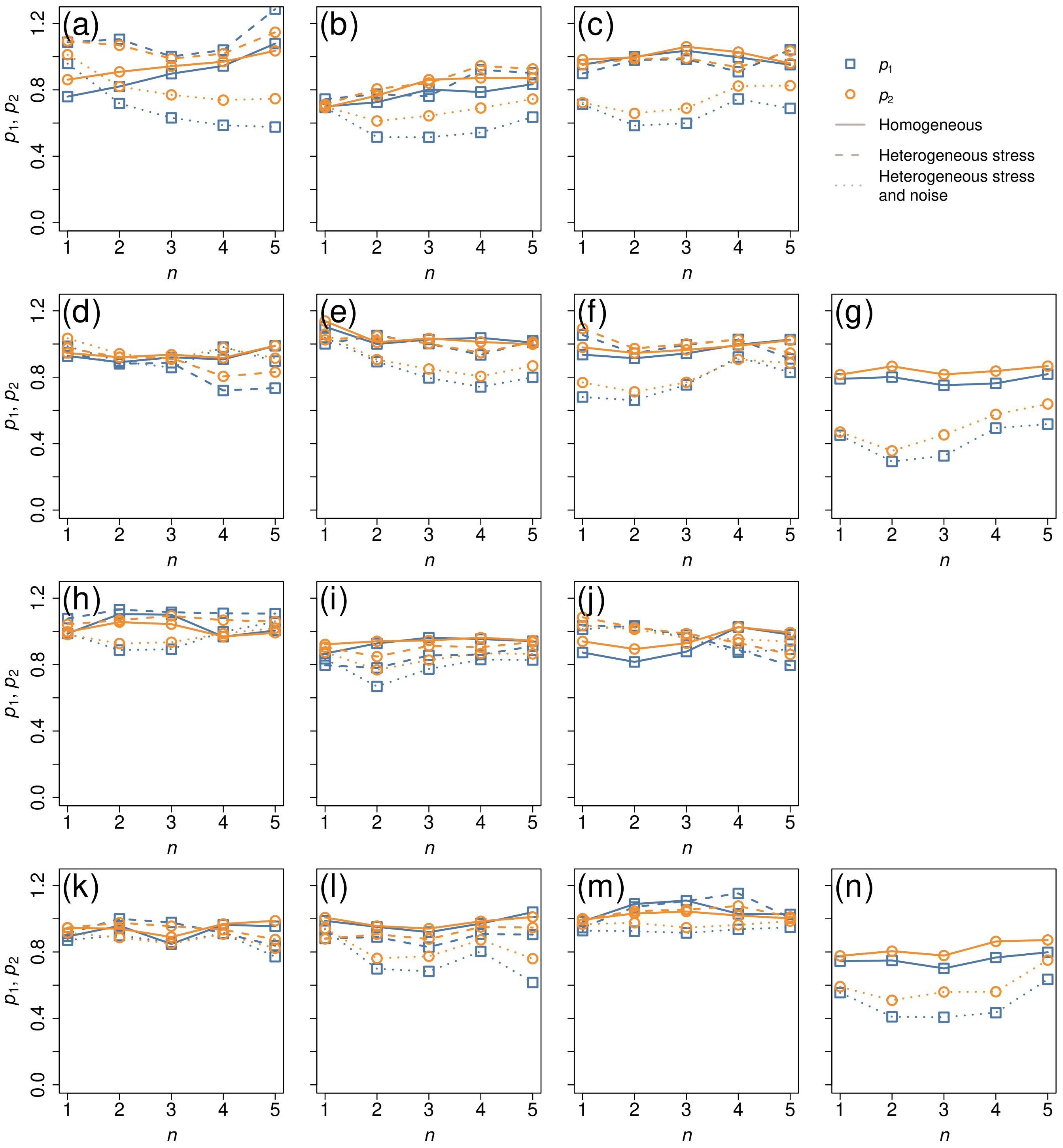

Figure S2: Performance of the node set maximizing d on the BA and Chesapeake Bay networks when $k^{(1)} = 0.1\tilde{K}$ and $k^{(2)} = 0.3\tilde{K}$. The squares and circles represent p_1 and p_2, respectively, for the given n, dynamics, network, and condition (i.e., whether u_i or σ_i is homogeneously or heterogeneously distributed) averaged over 50 series of simulations. (a)–(g): BA network. (h)–(n): Chesapeake Bay network. (a) and (h): Double-well, u. (b) and (i): Mutualistic interaction, u. (c) and (j): Gene regulatory, u. (d) and (k): Double-well, D. (e) and (l): Mutualistic interaction, D. (f) and (m): Gene regulatory, D. (g) and (n): SIS, λ.

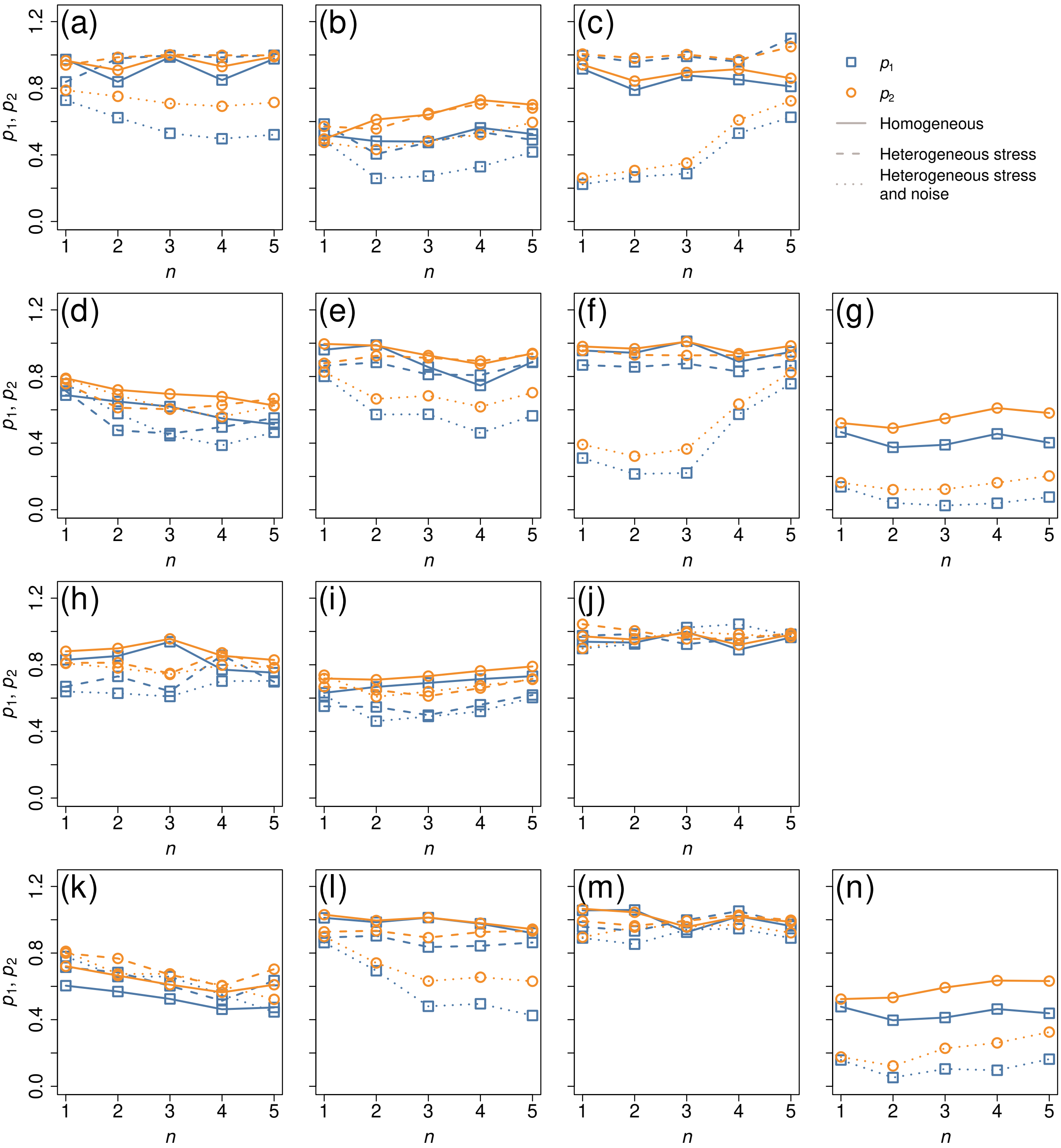

Figure S3: Performance of the node set maximizing d on the BA and Chesapeake Bay networks when $k^{(1)} = 0.1\tilde{K}$ and $k^{(2)} = 0.5\tilde{K}$. (a)–(g): BA network. (h)–(n): Chesapeake Bay network. (a) and (h): Double-well, u. (b) and (i): Mutualistic interaction, u. (c) and (j): Gene regulatory, u. (d) and (k): Double-well, D. (e) and (l): Mutualistic interaction, D. (f) and (m): Gene regulatory, D. (g) and (n): SIS, λ.

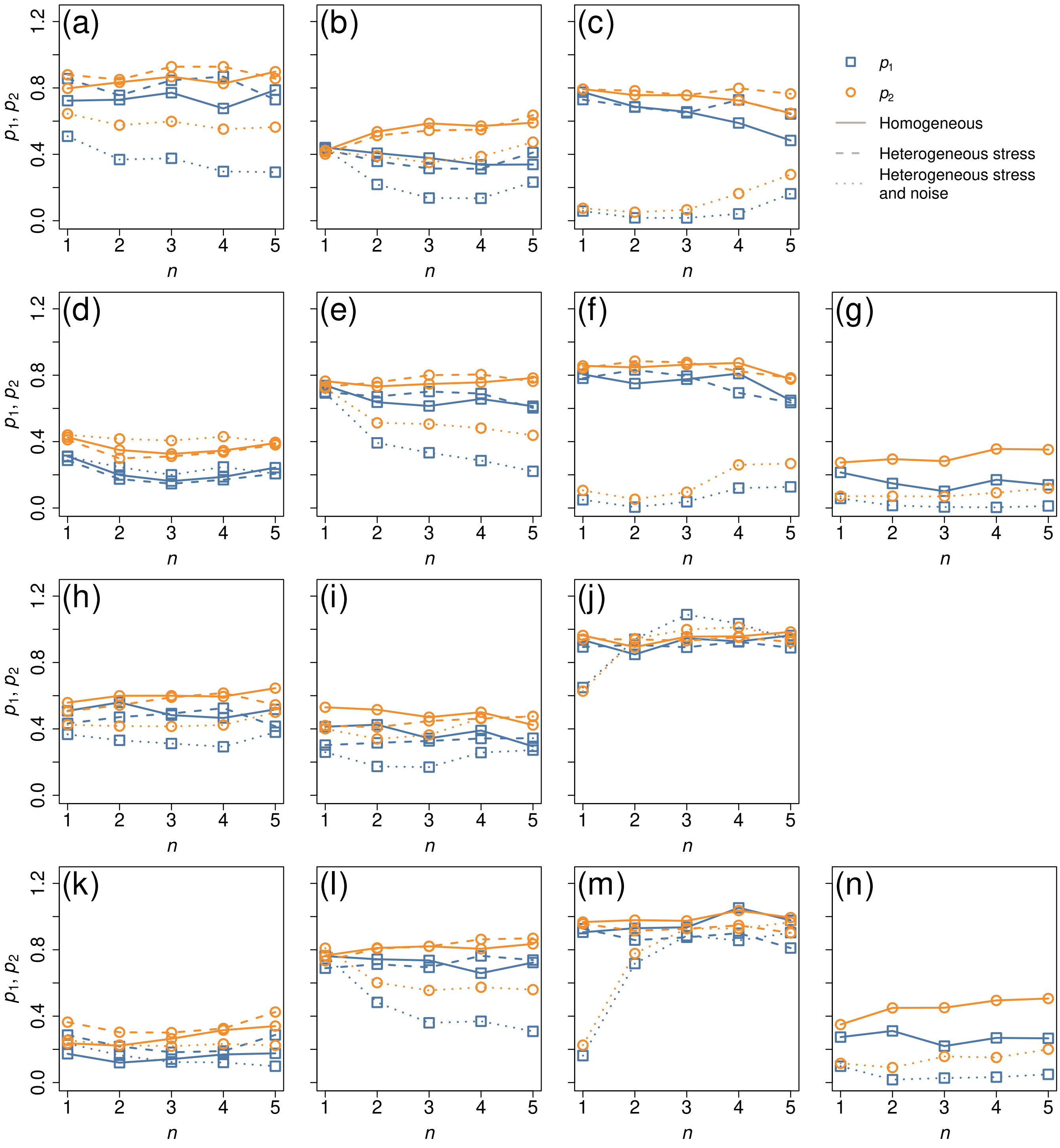

Figure S4: Performance of the node set maximizing d on the BA and Chesapeake Bay networks when $k^{(1)} = 0.1\tilde{K}$ and $k^{(2)} = 0.7\tilde{K}$. (a)–(g): BA network. (h)–(n): Chesapeake Bay network. (a) and (h): Double-well, u. (b) and (i): Mutualistic interaction, u. (c) and (j): Gene regulatory, u. (d) and (k): Double-well, D. (e) and (l): Mutualistic interaction, D. (f) and (m): Gene regulatory, D. (g) and (n): SIS, λ.

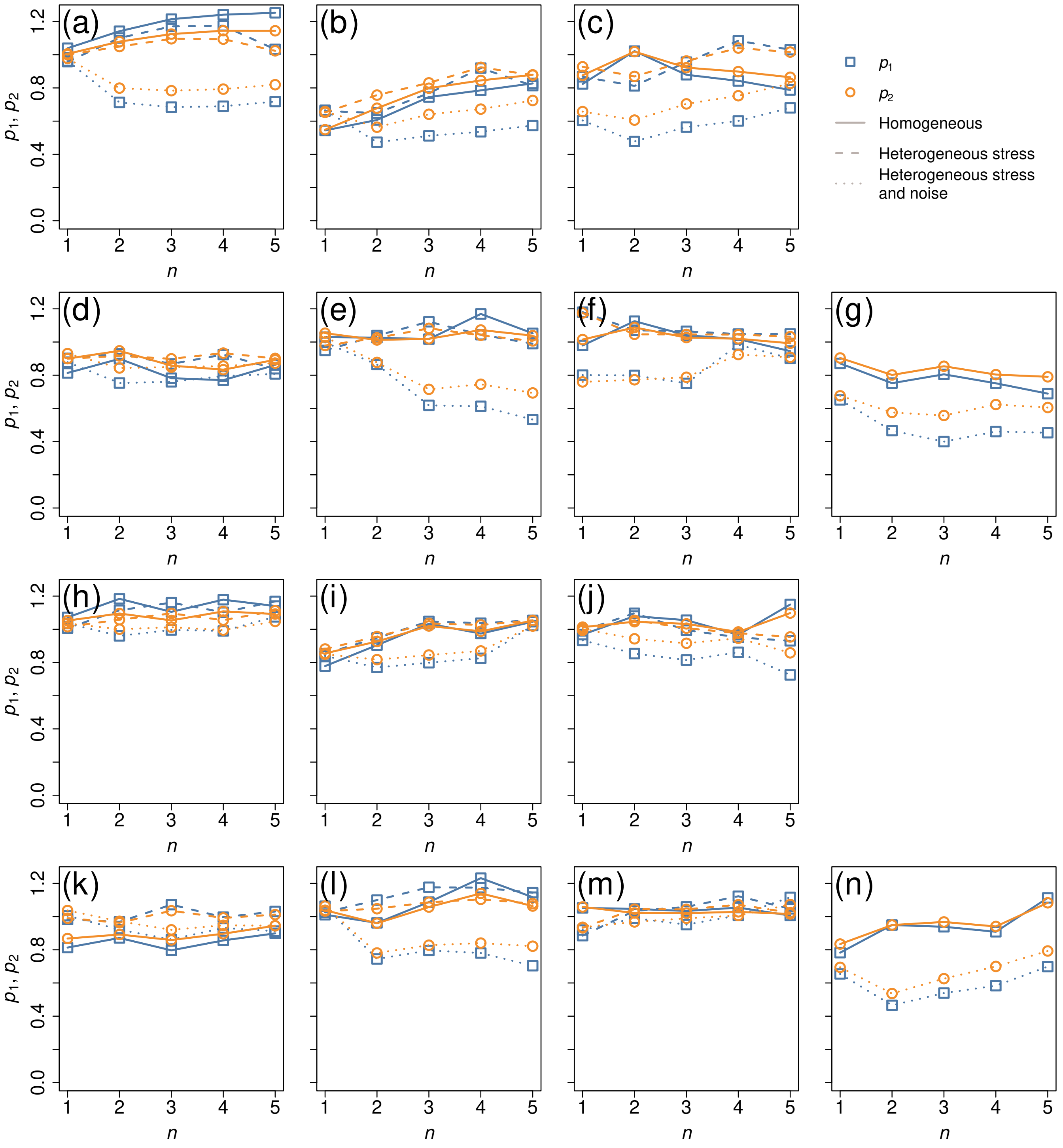

Figure S5: Performance of the node set maximizing d on the BA and Chesapeake Bay networks when $k^{(1)} = 0.3\tilde{K}$ and $k^{(2)} = 0.5\tilde{K}$. (a)–(g): BA network. (h)–(n): Chesapeake Bay network. (a) and (h): Double-well, u. (b) and (i): Mutualistic interaction, u. (c) and (j): Gene regulatory, u. (d) and (k): Double-well, D. (e) and (l): Mutualistic interaction, D. (f) and (m): Gene regulatory, D. (g) and (n): SIS, λ.

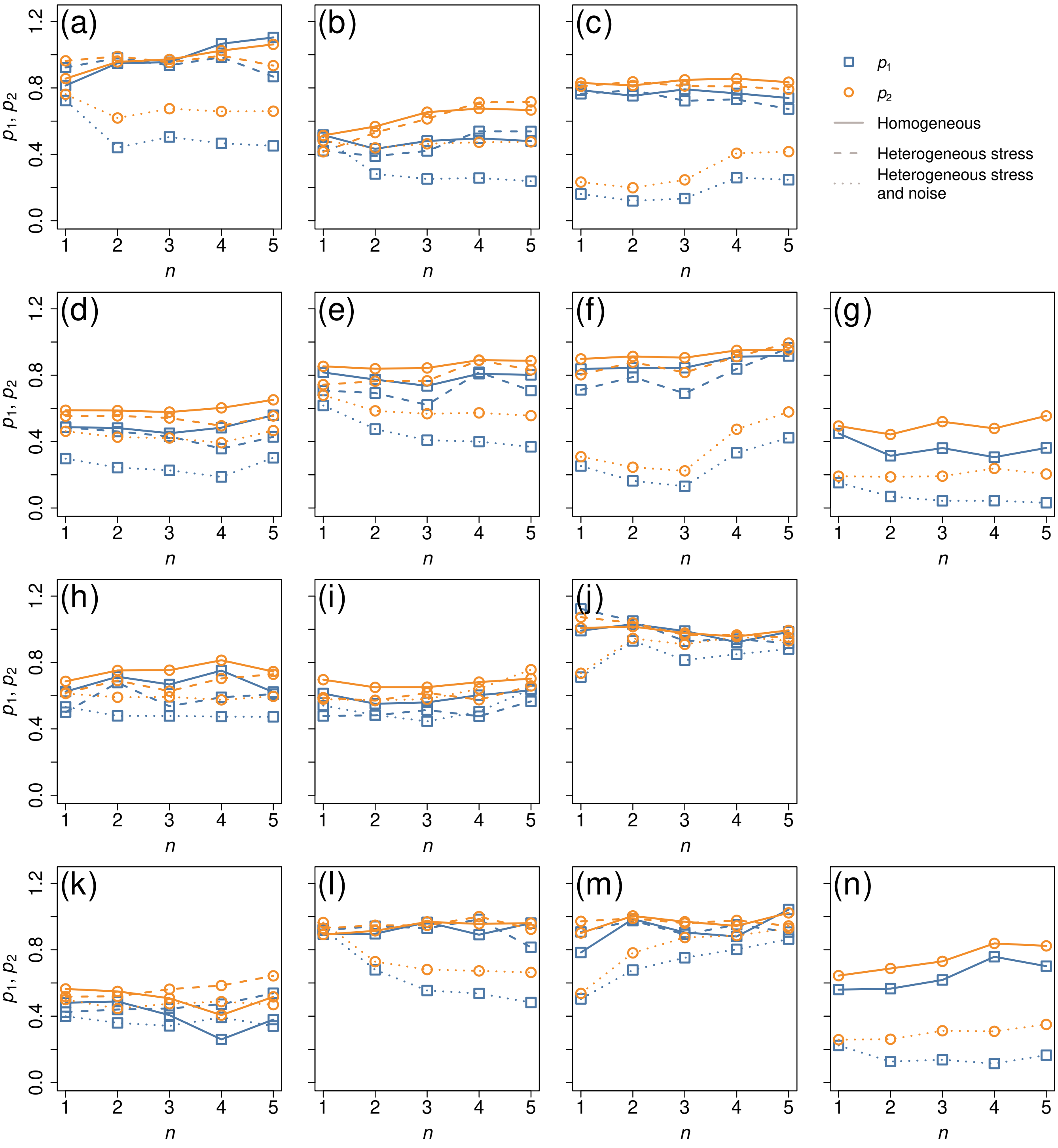

Figure S6: Performance of the node set maximizing d on the BA and Chesapeake Bay networks when $k^{(1)} = 0.3\tilde{K}$ and $k^{(2)} = 0.7\tilde{K}$. (a)–(g): BA network. (h)–(n): Chesapeake Bay network. (a) and (h): Double-well, u. (b) and (i): Mutualistic interaction, u. (c) and (j): Gene regulatory, u. (d) and (k): Double-well, D. (e) and (l): Mutualistic interaction, D. (f) and (m): Gene regulatory, D. (g) and (n): SIS, λ.

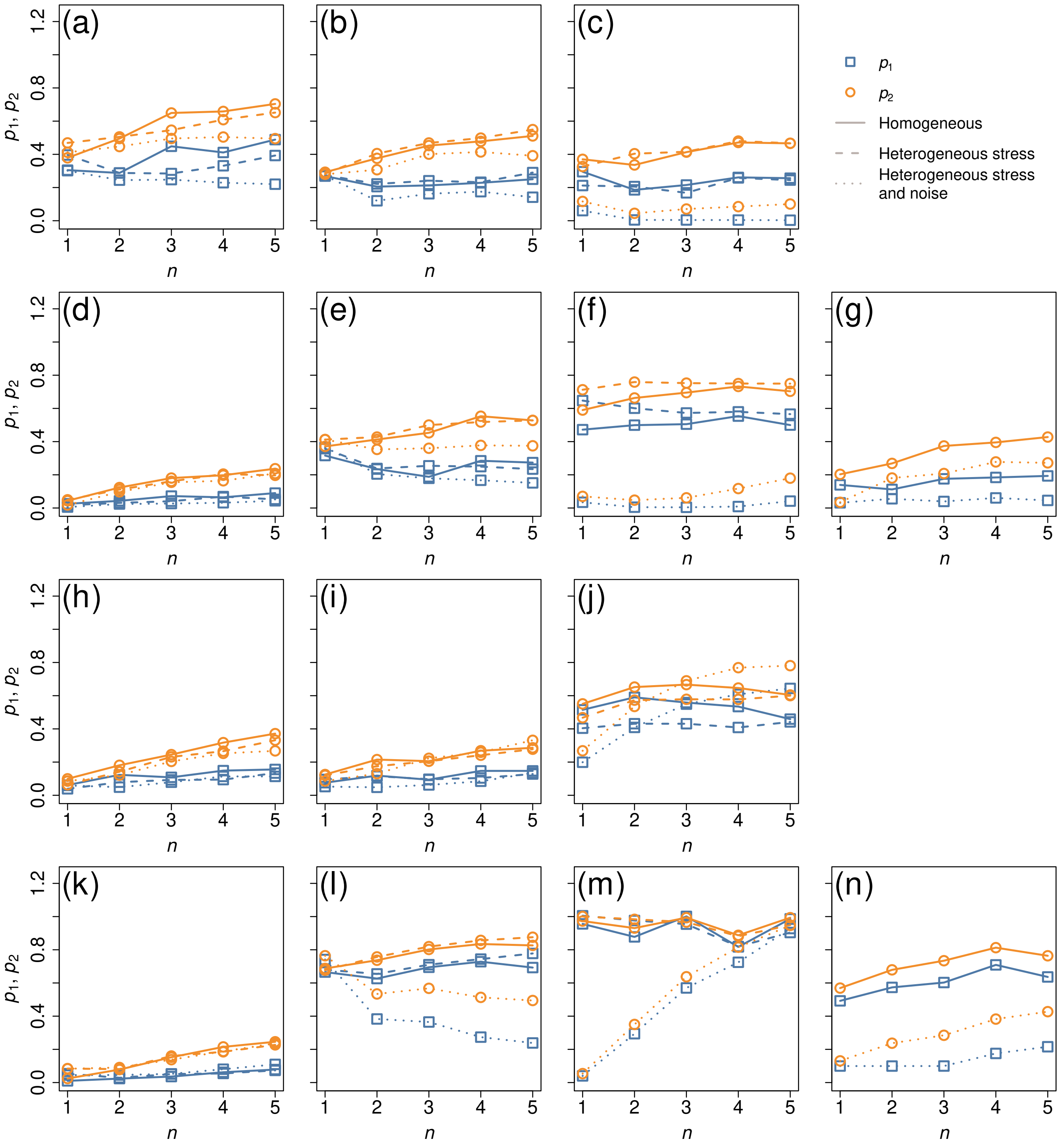

Figure S7: Performance of the node set maximizing d on the BA and Chesapeake Bay networks when $k^{(1)} = 0.3\tilde{K}$ and $k^{(2)} = 0.9\tilde{K}$. (a)–(g): BA network. (h)–(n): Chesapeake Bay network. (a) and (h): Double-well, u. (b) and (i): Mutualistic interaction, u. (c) and (j): Gene regulatory, u. (d) and (k): Double-well, D. (e) and (l): Mutualistic interaction, D. (f) and (m): Gene regulatory, D. (g) and (n): SIS, λ.

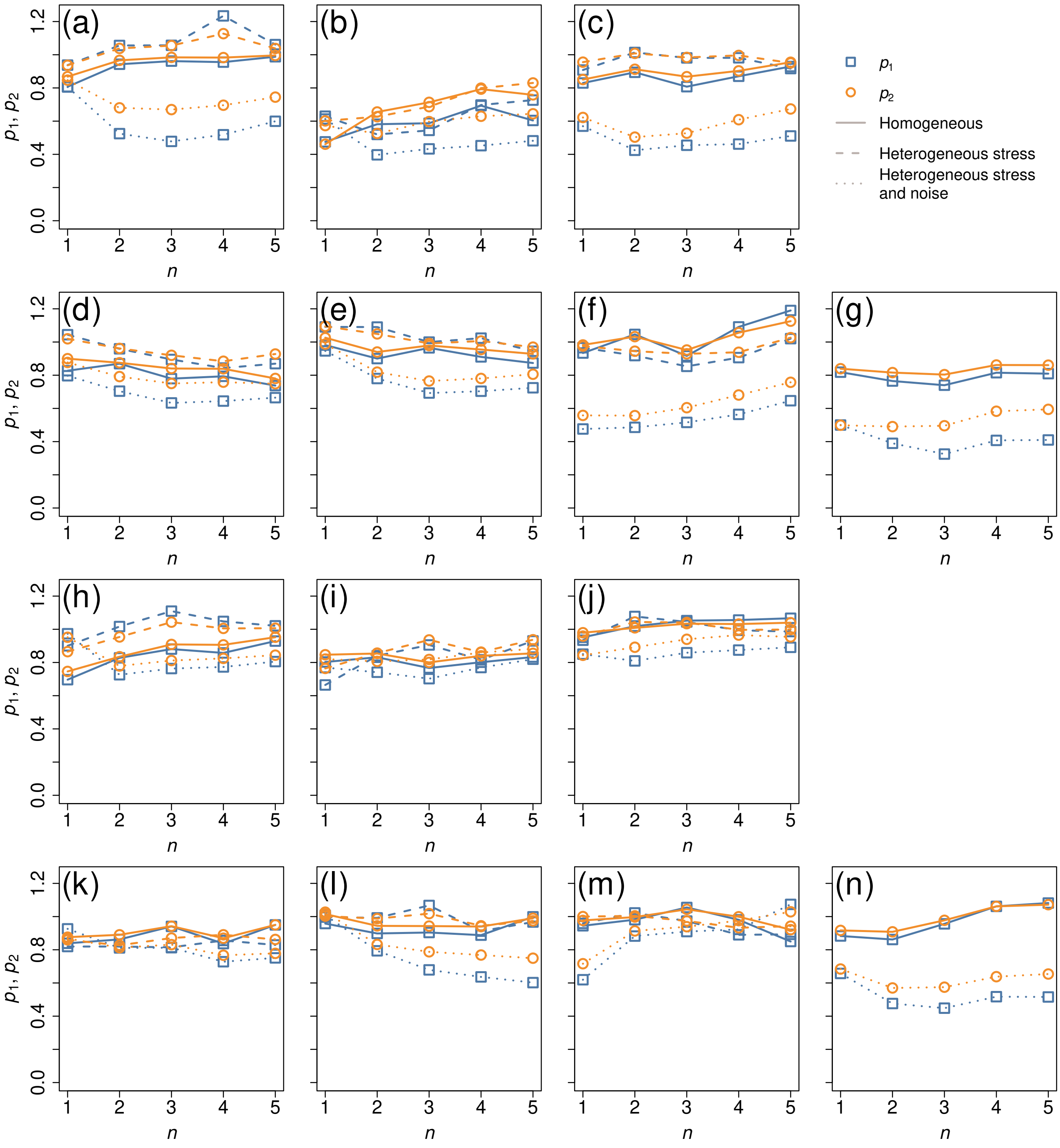

Figure S8: Performance of the node set maximizing d on the BA and Chesapeake Bay networks when $k^{(1)} = 0.5\tilde{K}$ and $k^{(2)} = 0.7\tilde{K}$. (a)–(g): BA network. (h)–(n): Chesapeake Bay network. (a) and (h): Double-well, u. (b) and (i): Mutualistic interaction, u. (c) and (j): Gene regulatory, u. (d) and (k): Double-well, D. (e) and (l): Mutualistic interaction, D. (f) and (m): Gene regulatory, D. (g) and (n): SIS, λ.

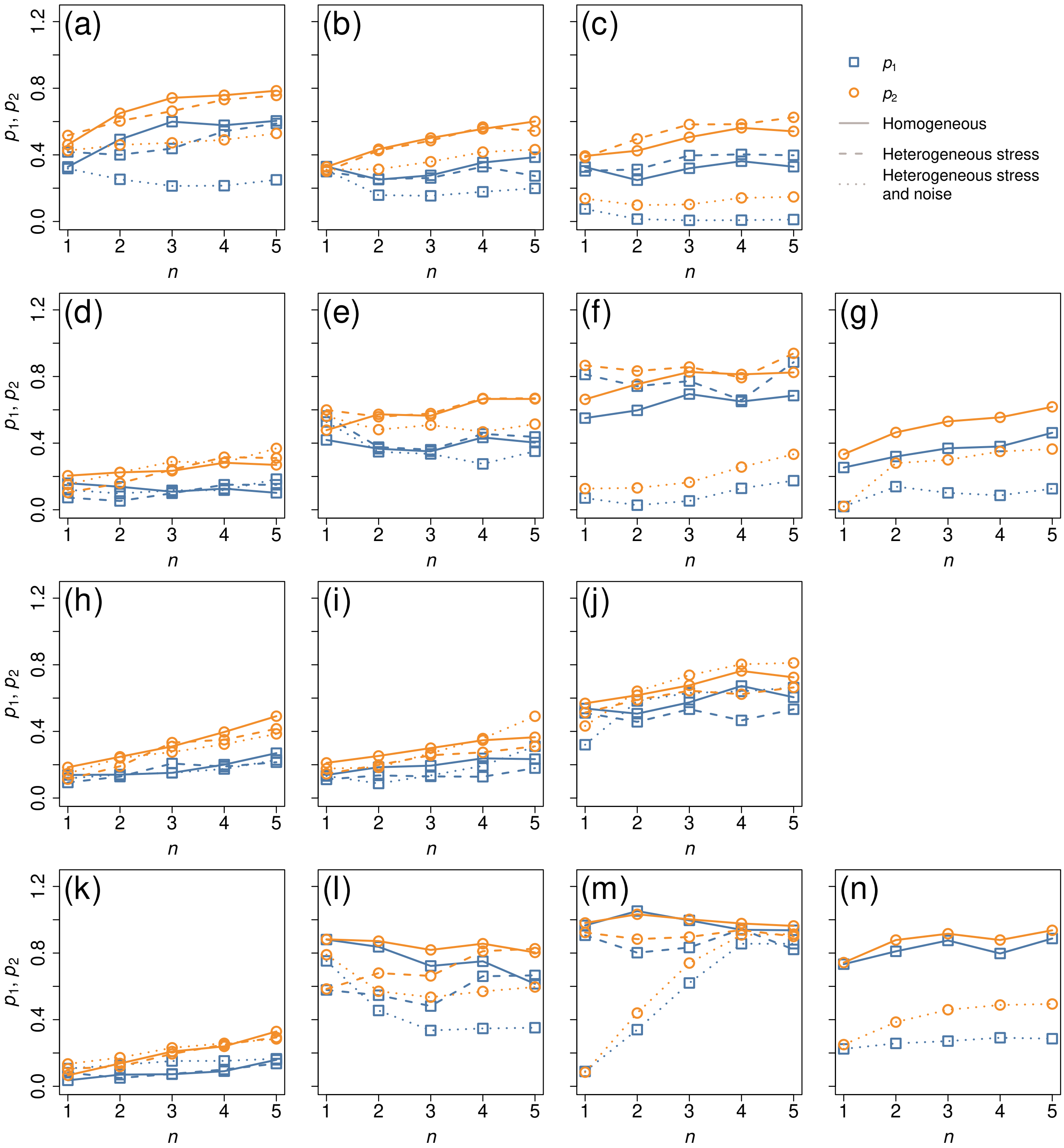

Figure S9: Performance of the node set maximizing d on the BA and Chesapeake Bay networks when $k^{(1)} = 0.5\tilde{K}$ and $k^{(2)} = 0.9\tilde{K}$. (a)–(g): BA network. (h)–(n): Chesapeake Bay network. (a) and (h): Double-well, u. (b) and (i): Mutualistic interaction, u. (c) and (j): Gene regulatory, u. (d) and (k): Double-well, D. (e) and (l): Mutualistic interaction, D. (f) and (m): Gene regulatory, D. (g) and (n): SIS, λ.

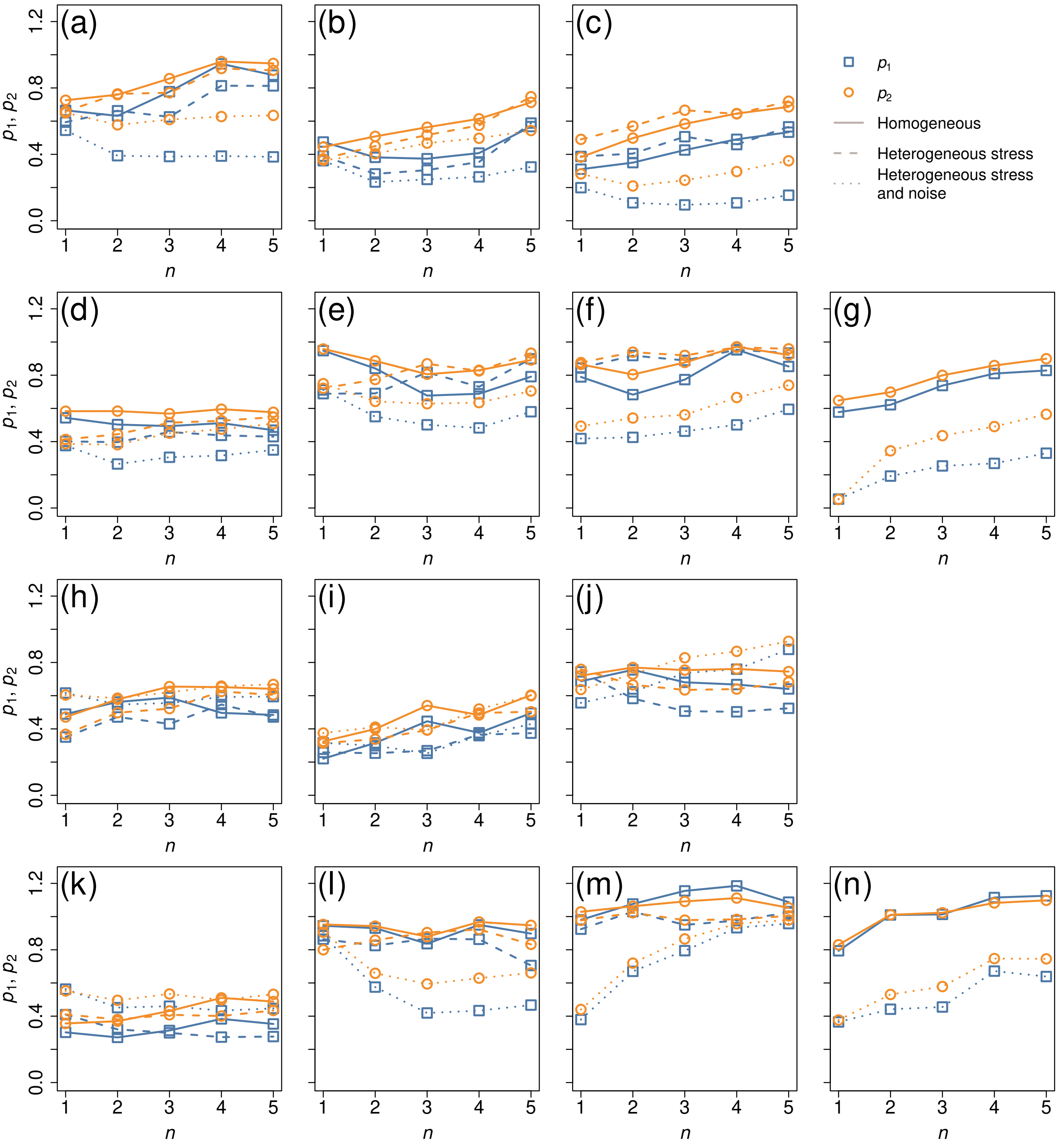

Figure S10: Performance of the node set maximizing d on the BA and Chesapeake Bay networks when $k^{(1)} = 0.7\tilde{K}$ and $k^{(2)} = 0.9\tilde{K}$. (a)–(g): BA network. (h)–(n): Chesapeake Bay network. (a) and (h): Double-well, u. (b) and (i): Mutualistic interaction, u. (c) and (j): Gene regulatory, u. (d) and (k): Double-well, D. (e) and (l): Mutualistic interaction, D. (f) and (m): Gene regulatory, D. (g) and (n): SIS, λ.

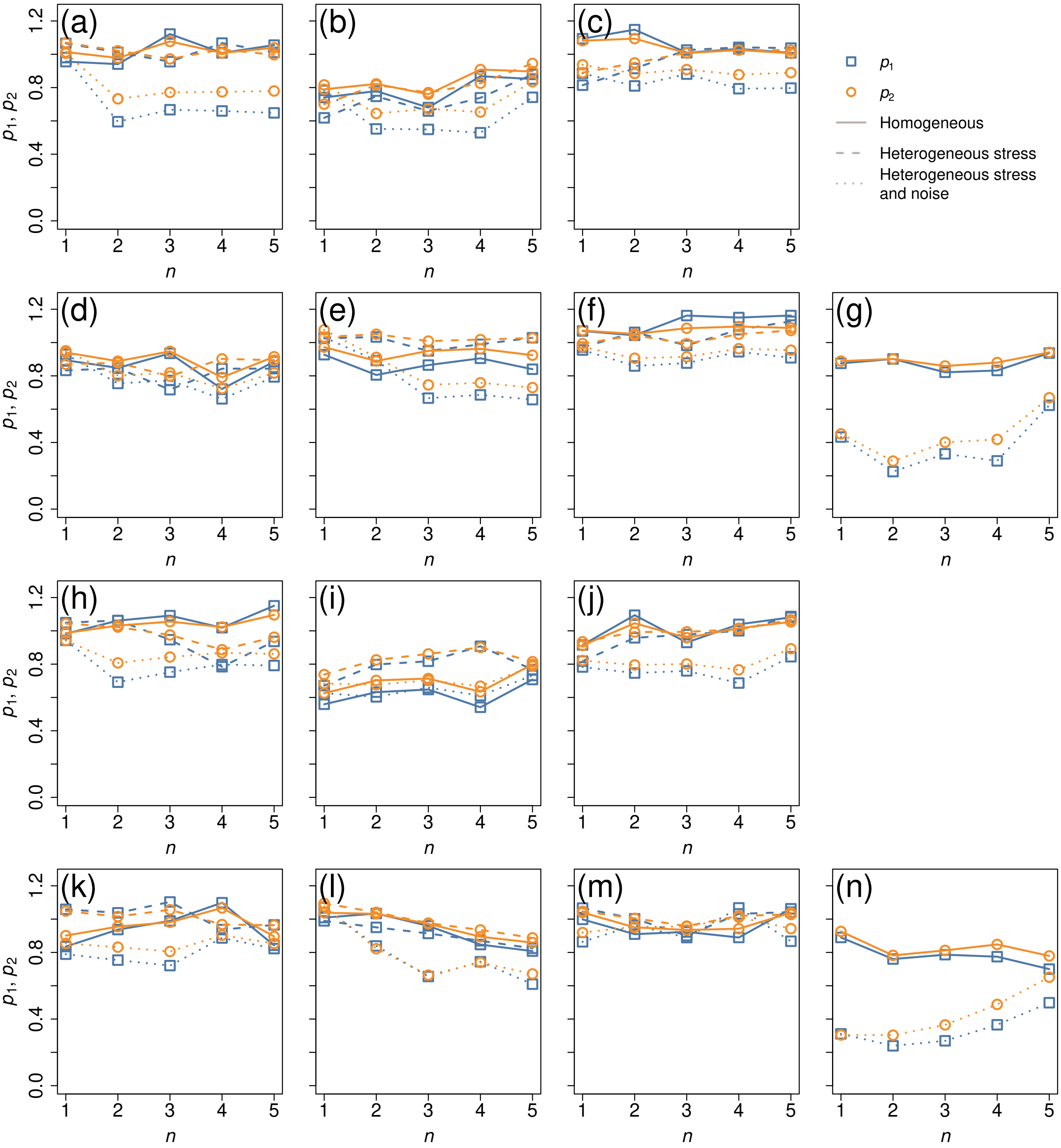

Figure S11: Performance of the node set maximizing d on the Erdős-Rényi and node fitness networks when $k^{(1)} = 0.1\tilde{K}$ and $k^{(2)} = 0.3\tilde{K}$. (a)–(g): Erdős-Rényi network. (h)–(n): Node fitness network. (a) and (h): Double-well, u. (b) and (i): Mutualistic interaction, u. (c) and (j): Gene regulatory, u. (d) and (k): Double-well, D. (e) and (l): Mutualistic interaction, D. (f) and (m): Gene regulatory, D. (g) and (n): SIS, λ.

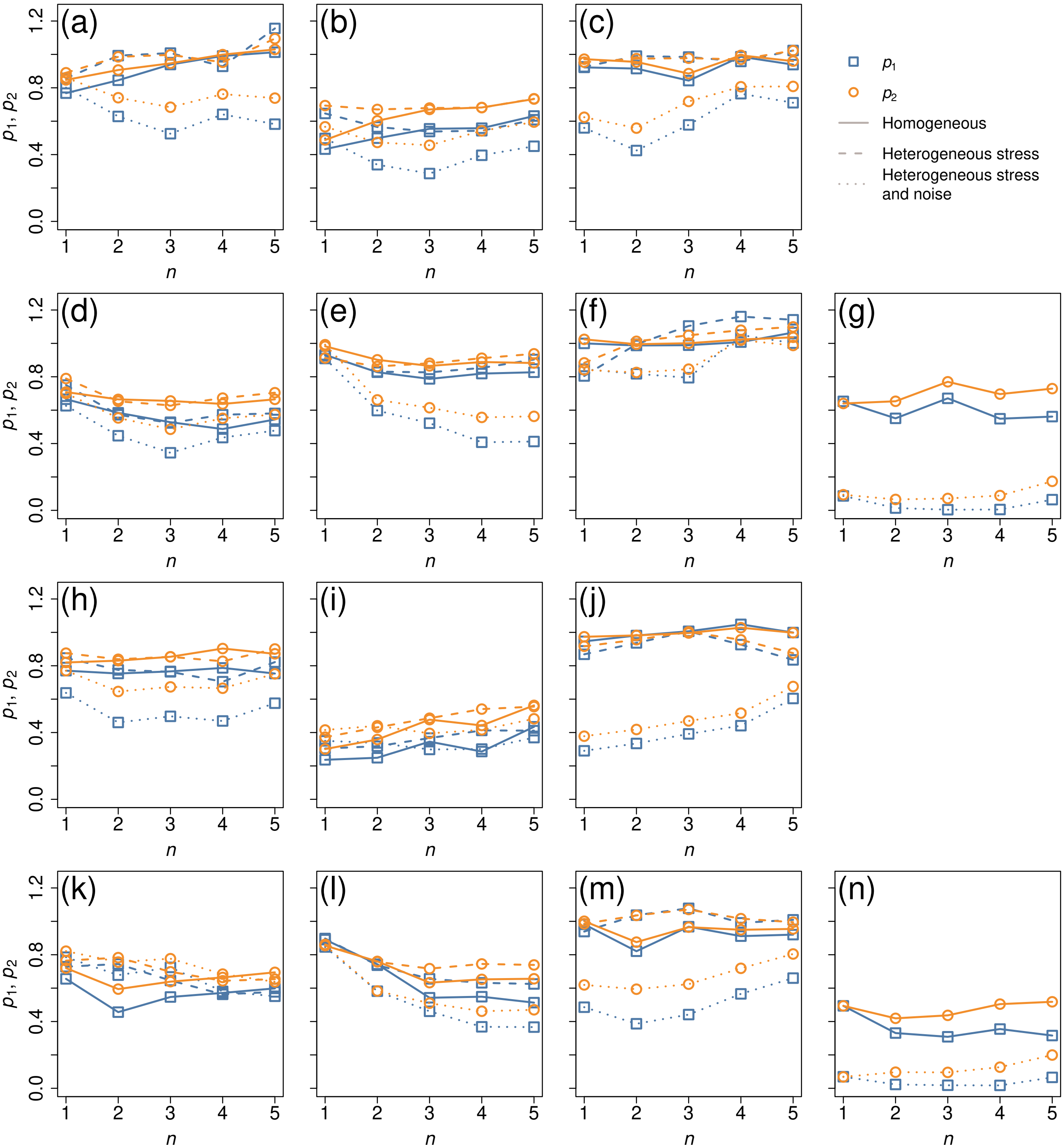

Figure S12: Performance of the node set maximizing d on the Erdős-Rényi and node fitness networks when $k^{(1)} = 0.1\tilde{K}$ and $k^{(2)} = 0.5\tilde{K}$. (a)–(g): Erdős-Rényi network. (h)–(n): Node fitness network. (a) and (h): Double-well, u. (b) and (i): Mutualistic interaction, u. (c) and (j): Gene regulatory, u. (d) and (k): Double-well, D. (e) and (l): Mutualistic interaction, D. (f) and (m): Gene regulatory, D. (g) and (n): SIS, λ.

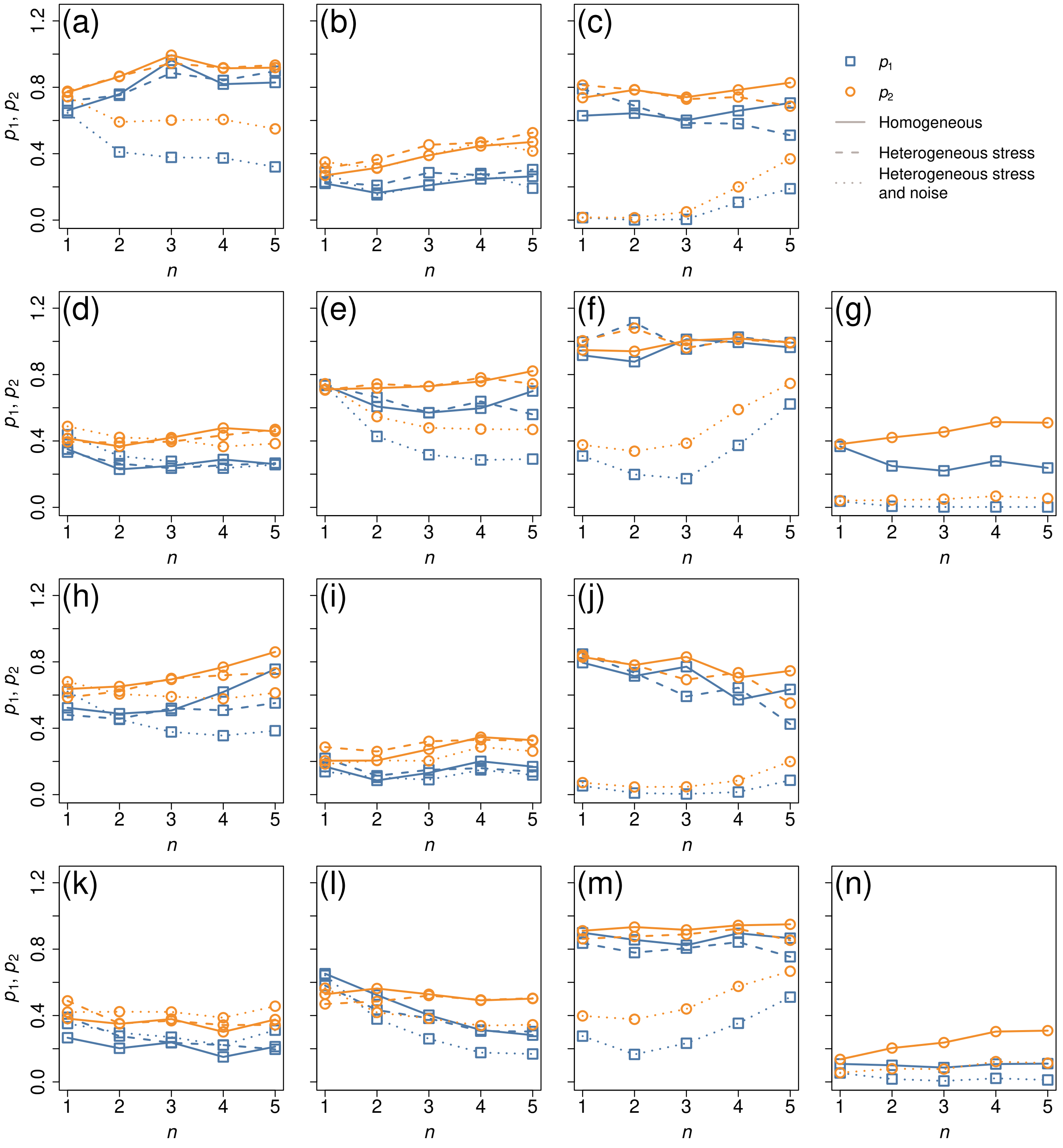

Figure S13: Performance of the node set maximizing d on the Erdős-Rényi and node fitness networks when $k^{(1)} = 0.1\tilde{K}$ and $k^{(2)} = 0.7\tilde{K}$. (a)–(g): Erdős-Rényi network. (h)–(n): Node fitness network. (a) and (h): Double-well, u. (b) and (i): Mutualistic interaction, u. (c) and (j): Gene regulatory, u. (d) and (k): Double-well, D. (e) and (l): Mutualistic interaction, D. (f) and (m): Gene regulatory, D. (g) and (n): SIS, λ.

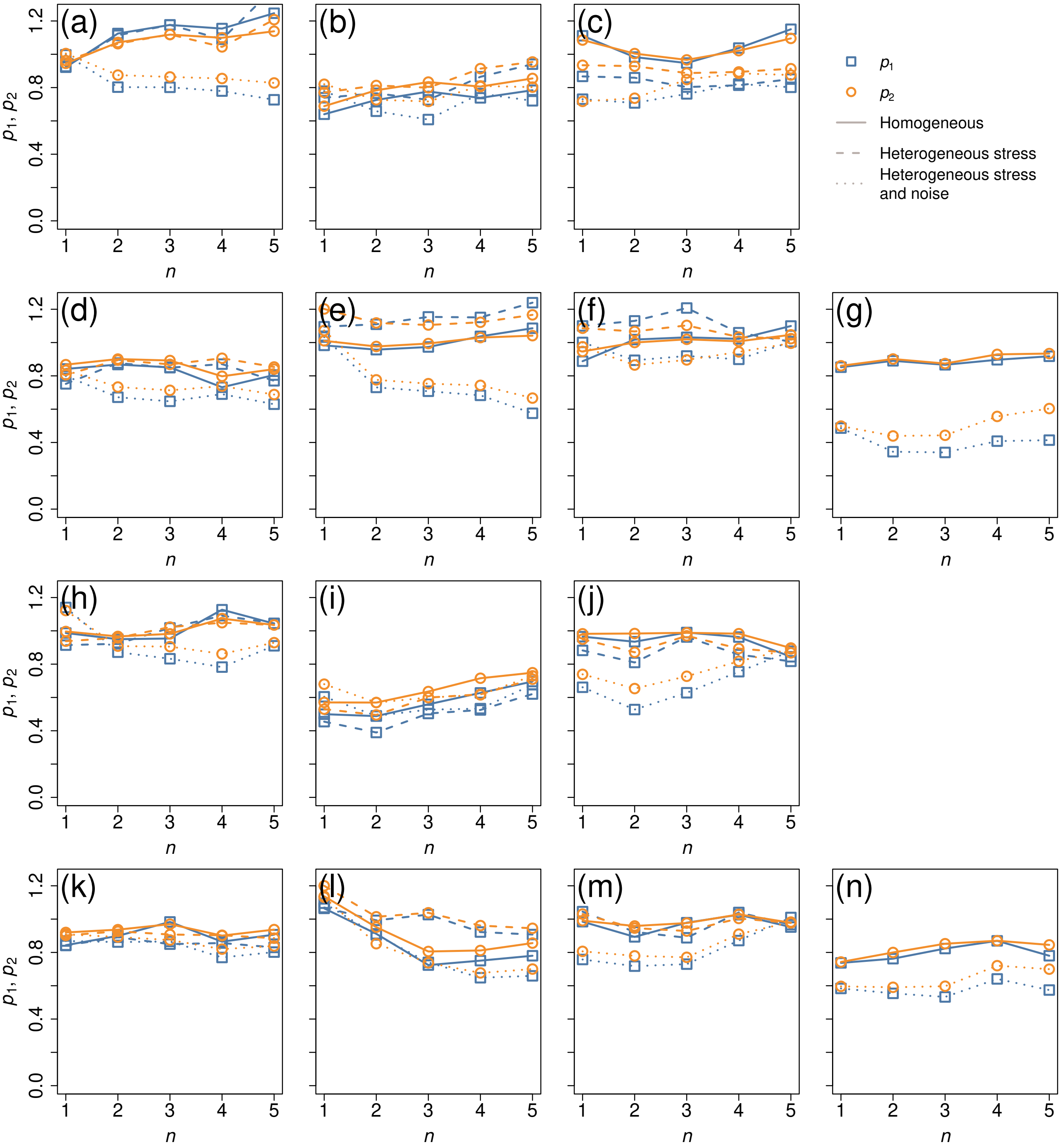

Figure S14: Performance of the node set maximizing d on the Erdős-Rényi and node fitness networks when $k^{(1)} = 0.3\tilde{K}$ and $k^{(2)} = 0.5\tilde{K}$. (a)–(g): Erdős-Rényi network. (h)–(n): Node fitness network. (a) and (h): Double-well, u. (b) and (i): Mutualistic interaction, u. (c) and (j): Gene regulatory, u. (d) and (k): Double-well, D. (e) and (l): Mutualistic interaction, D. (f) and (m): Gene regulatory, D. (g) and (n): SIS, λ.

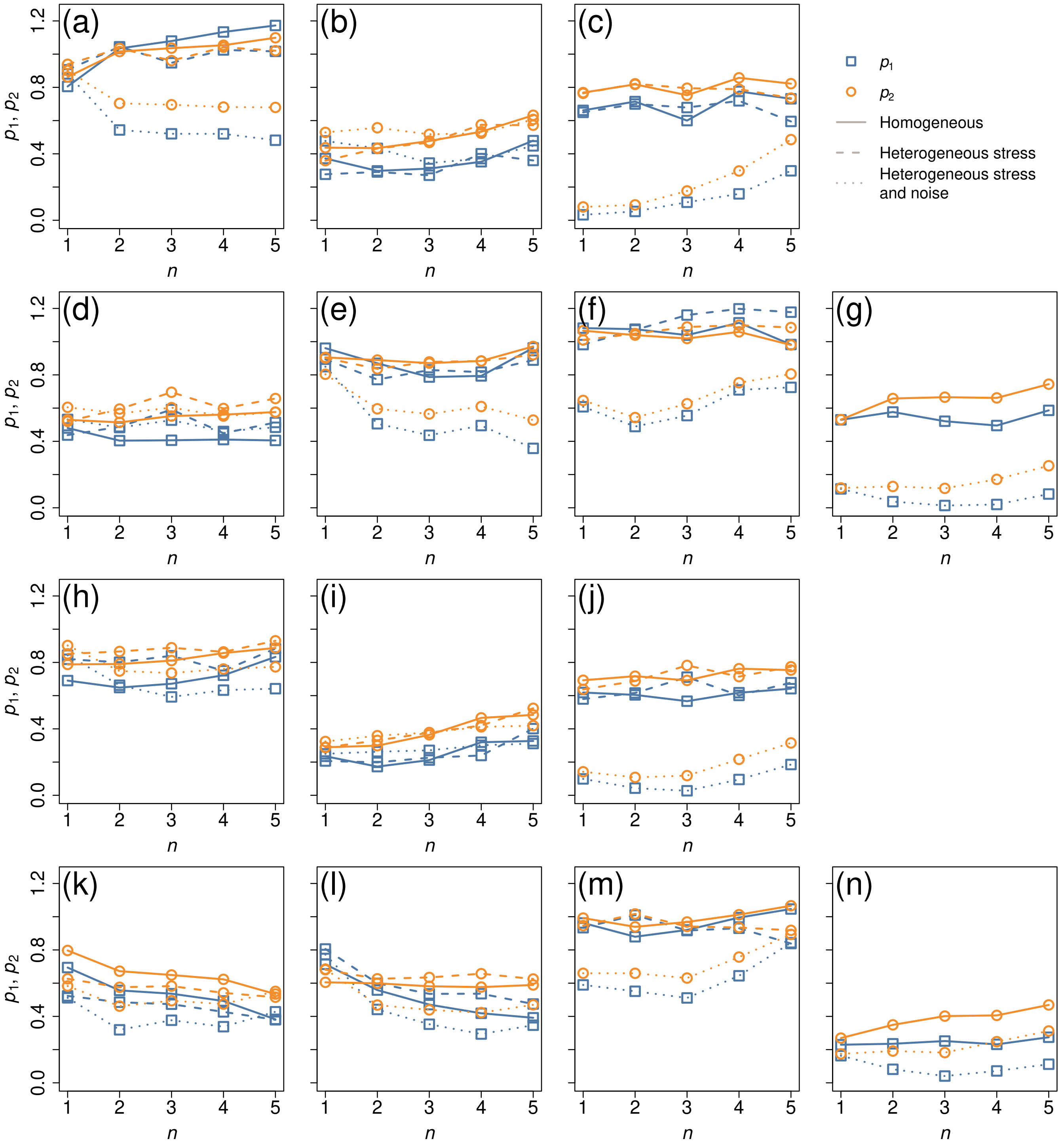

Figure S15: Performance of the node set maximizing d on the Erdős-Rényi and node fitness networks when $k^{(1)} = 0.3\tilde{K}$ and $k^{(2)} = 0.7\tilde{K}$. (a)–(g): Erdős-Rényi network. (h)–(n): Node fitness network. (a) and (h): Double-well, u. (b) and (i): Mutualistic interaction, u. (c) and (j): Gene regulatory, u. (d) and (k): Double-well, D. (e) and (l): Mutualistic interaction, D. (f) and (m): Gene regulatory, D. (g) and (n): SIS, λ.

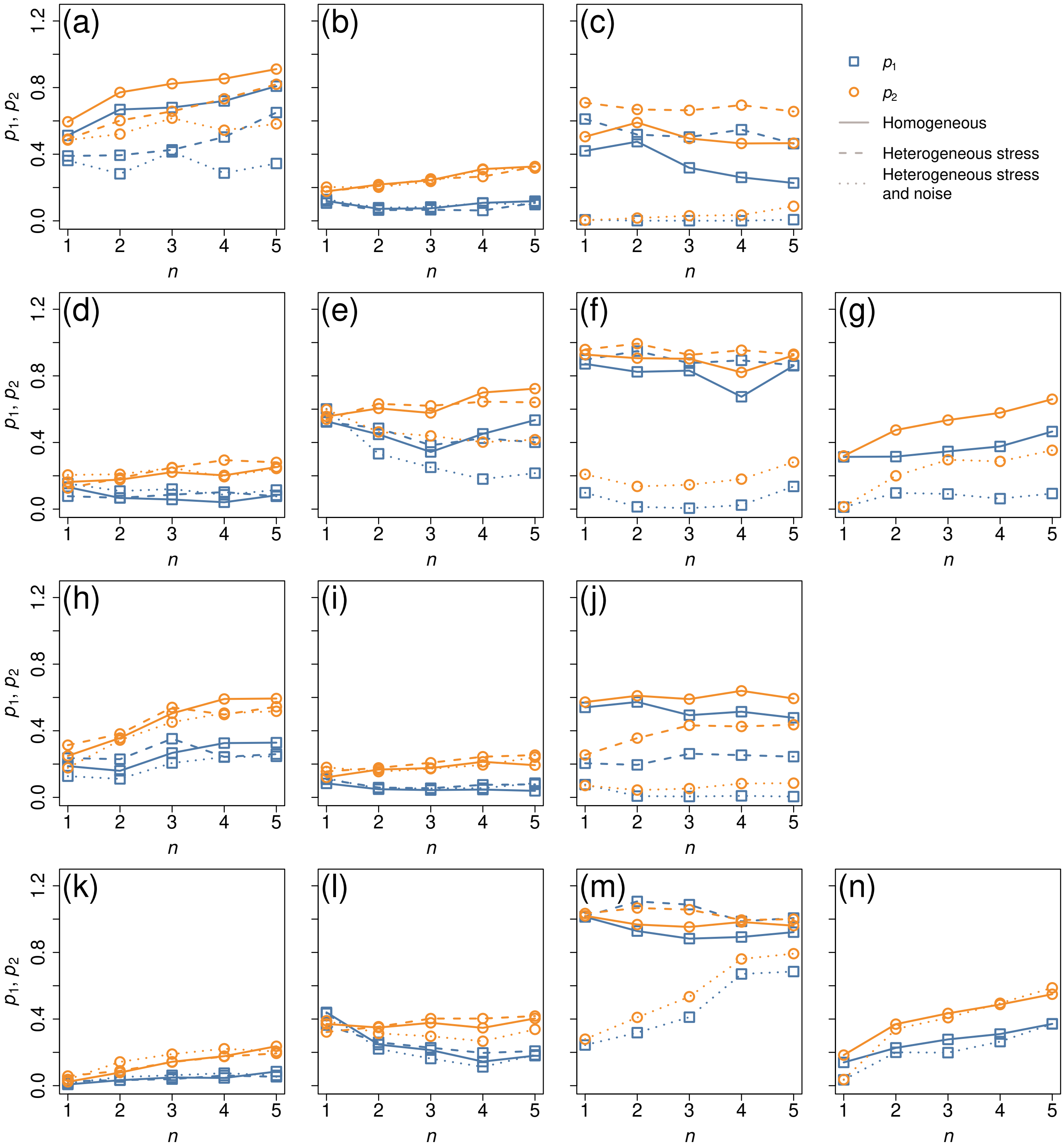

Figure S16: Performance of the node set maximizing d on the Erdős-Rényi and node fitness networks when $k^{(1)} = 0.3\tilde{K}$ and $k^{(2)} = 0.9\tilde{K}$. (a)–(g): Erdős-Rényi network. (h)–(n): Node fitness network. (a) and (h): Double-well, u. (b) and (i): Mutualistic interaction, u. (c) and (j): Gene regulatory, u. (d) and (k): Double-well, D. (e) and (l): Mutualistic interaction, D. (f) and (m): Gene regulatory, D. (g) and (n): SIS, λ.

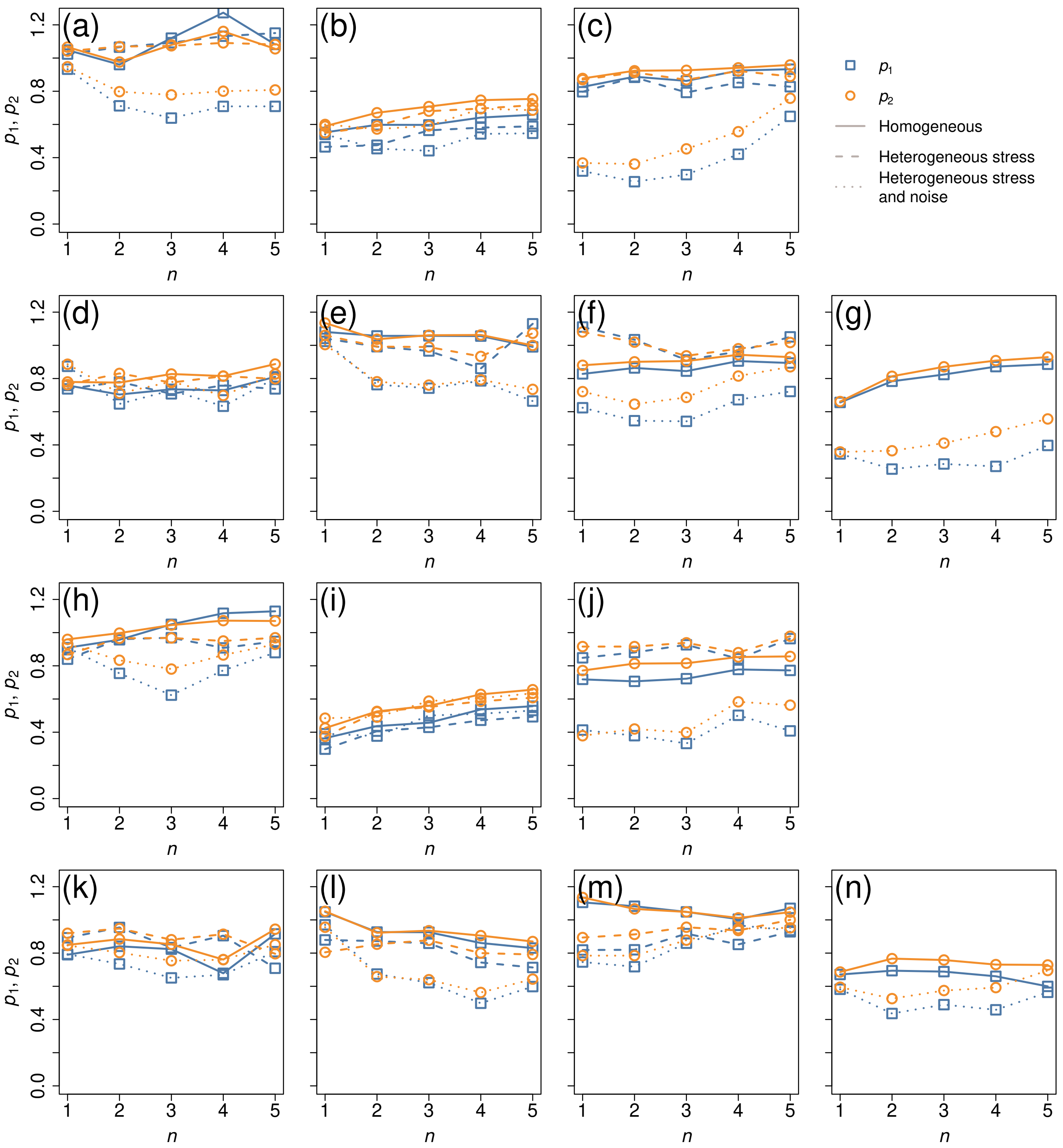

Figure S17: Performance of the node set maximizing d on the Erdős-Rényi and node fitness networks when $k^{(1)} = 0.5\tilde{K}$ and $k^{(2)} = 0.7\tilde{K}$. (a)–(g): Erdős-Rényi network. (h)–(n): Node fitness network. (a) and (h): Double-well, u. (b) and (i): Mutualistic interaction, u. (c) and (j): Gene regulatory, u. (d) and (k): Double-well, D. (e) and (l): Mutualistic interaction, D. (f) and (m): Gene regulatory, D. (g) and (n): SIS, λ.

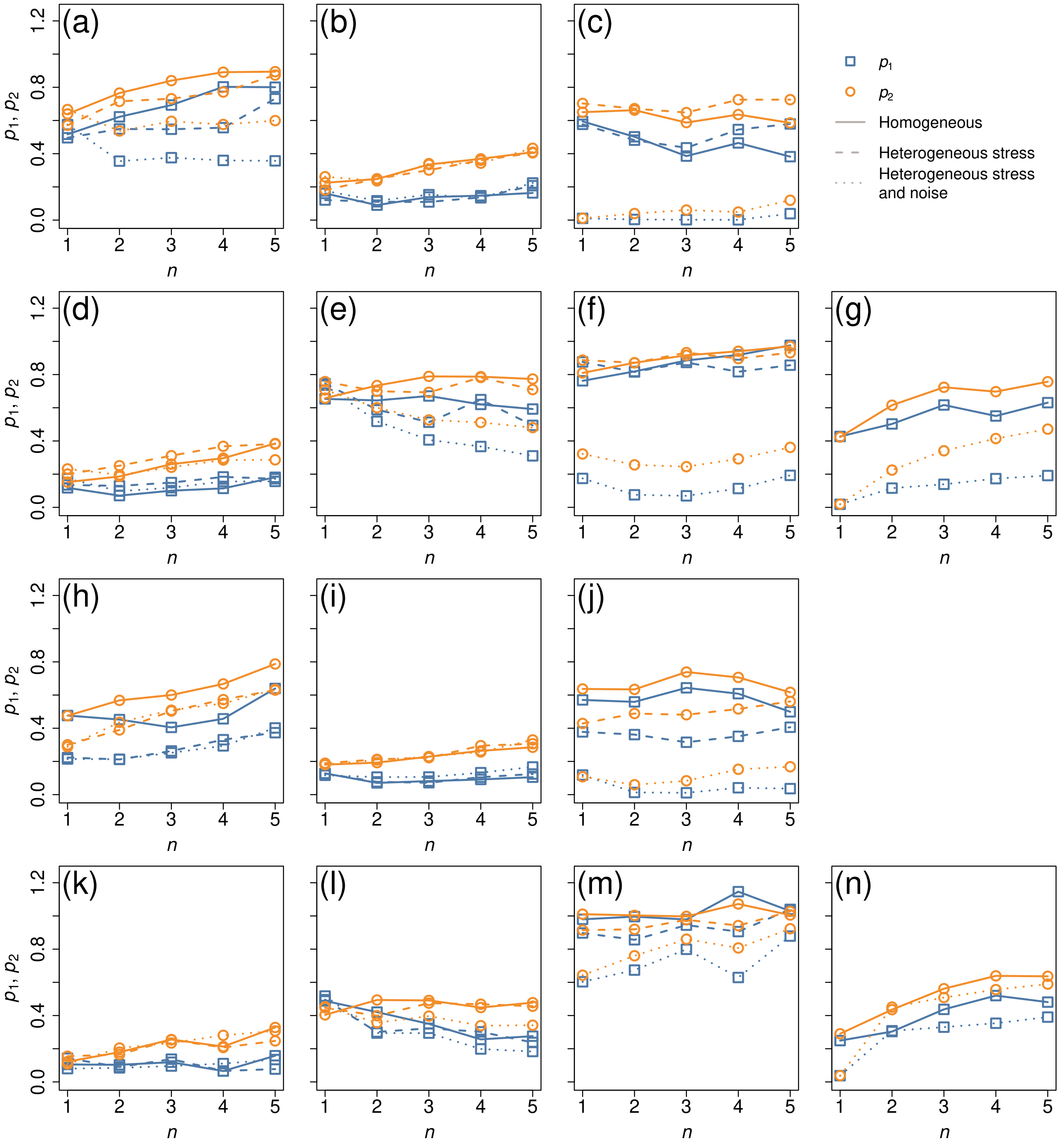

Figure S18: Performance of the node set maximizing d on the Erdős-Rényi and node fitness networks when $k^{(1)} = 0.5\tilde{K}$ and $k^{(2)} = 0.9\tilde{K}$. (a)–(g): Erdős-Rényi network. (h)–(n): Node fitness network. (a) and (h): Double-well, u. (b) and (i): Mutualistic interaction, u. (c) and (j): Gene regulatory, u. (d) and (k): Double-well, D. (e) and (l): Mutualistic interaction, D. (f) and (m): Gene regulatory, D. (g) and (n): SIS, λ.

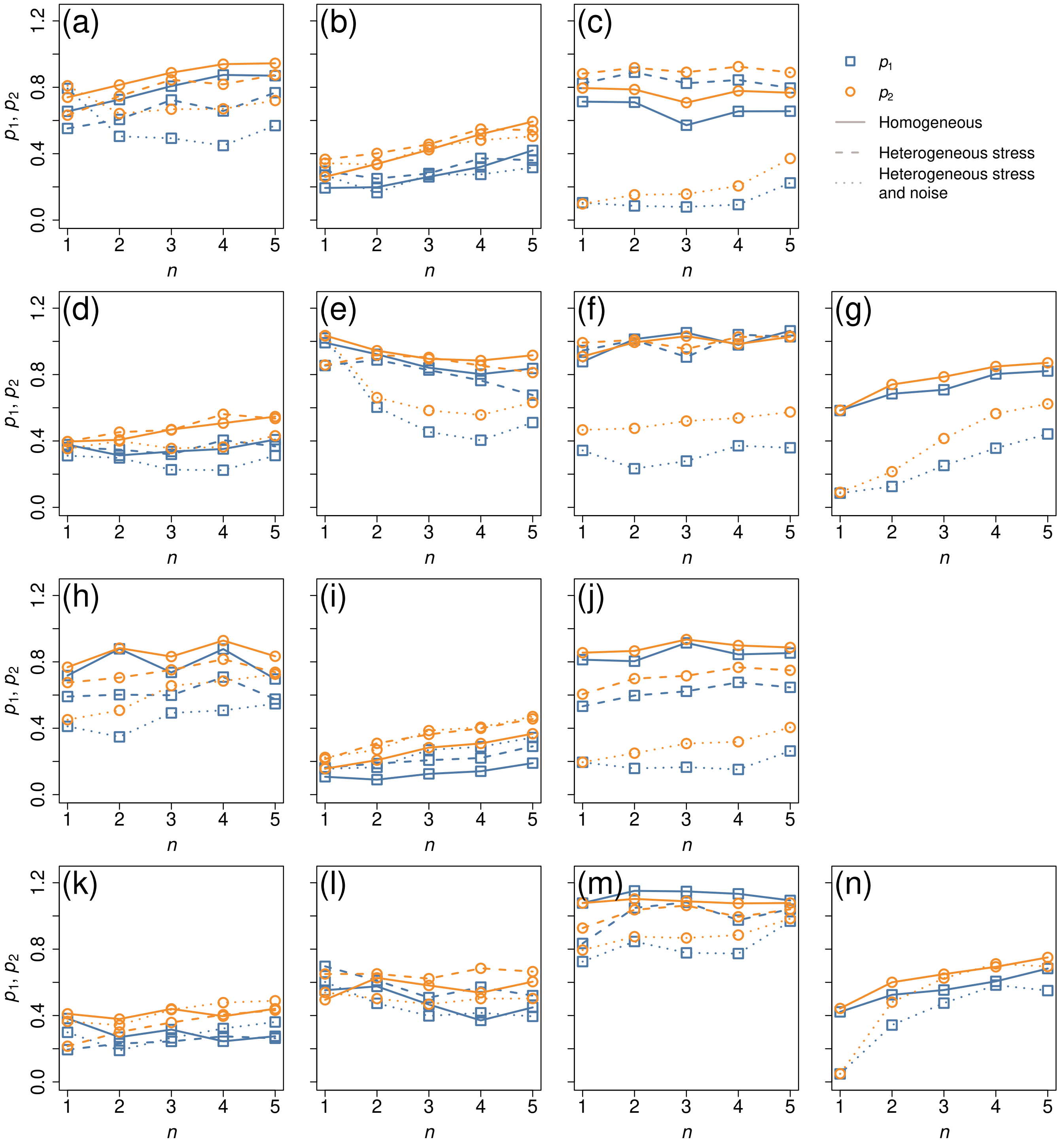

Figure S19: Performance of the node set maximizing d on the Erdős-Rényi and node fitness networks when $k^{(1)} = 0.7\tilde{K}$ and $k^{(2)} = 0.9\tilde{K}$. (a)–(g): Erdős-Rényi network. (h)–(n): Node fitness network. (a) and (h): Double-well, u. (b) and (i): Mutualistic interaction, u. (c) and (j): Gene regulatory, u. (d) and (k): Double-well, D. (e) and (l): Mutualistic interaction, D. (f) and (m): Gene regulatory, D. (g) and (n): SIS, λ.

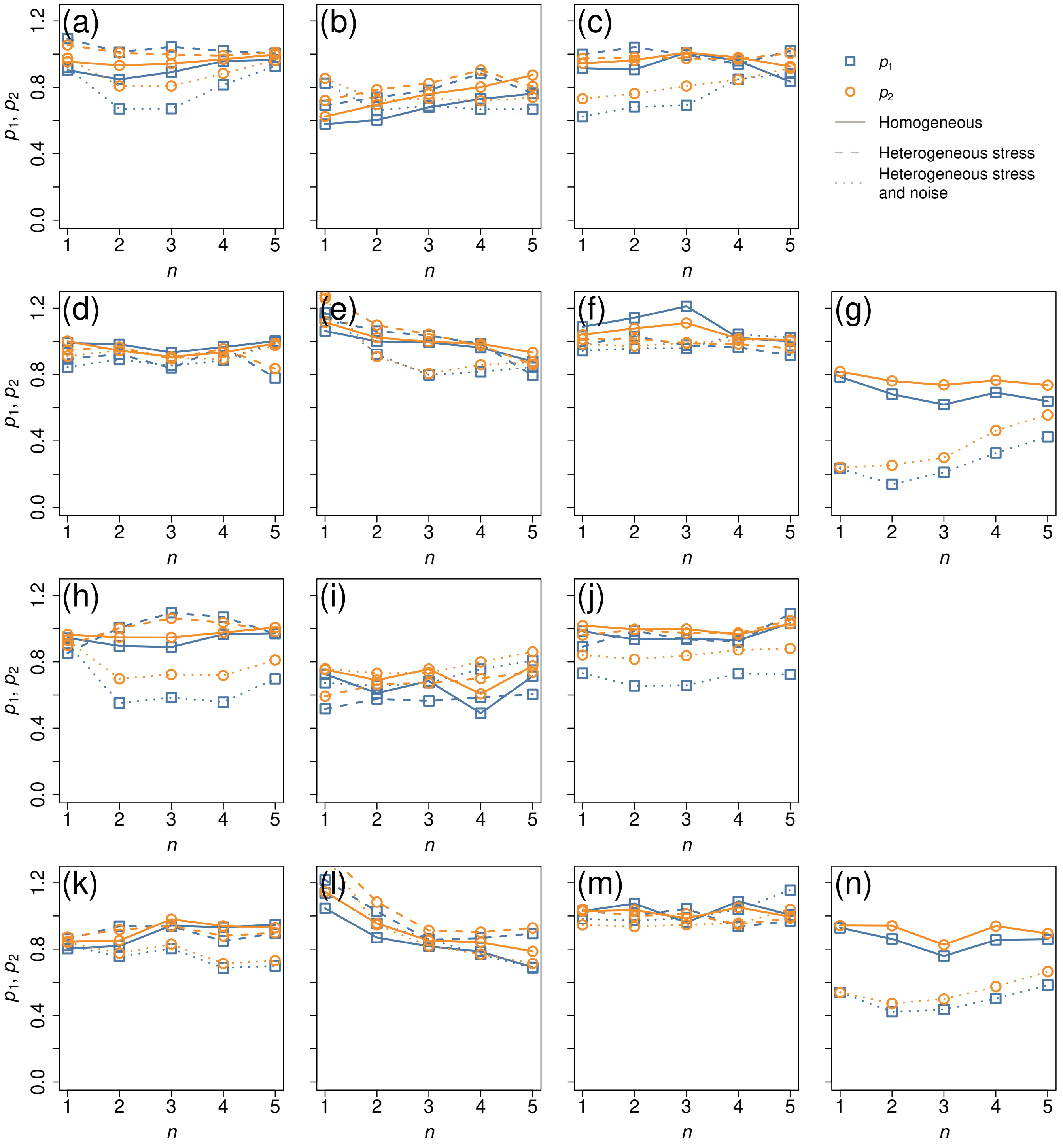

Figure S20: Performance of the node set maximizing d on the freshwater stream food web and dolphin social networks when $k^{(1)} = 0.1\tilde{K}$ and $k^{(2)} = 0.3\tilde{K}$. (a)–(g): Freshwater stream food web network. (h)–(n): Dolphin social network. (a) and (h): Double-well, u. (b) and (i): Mutualistic interaction, u. (c) and (j): Gene regulatory, u. (d) and (k): Double-well, D. (e) and (l): Mutualistic interaction, D. (f) and (m): Gene regulatory, D. (g) and (n): SIS, λ.

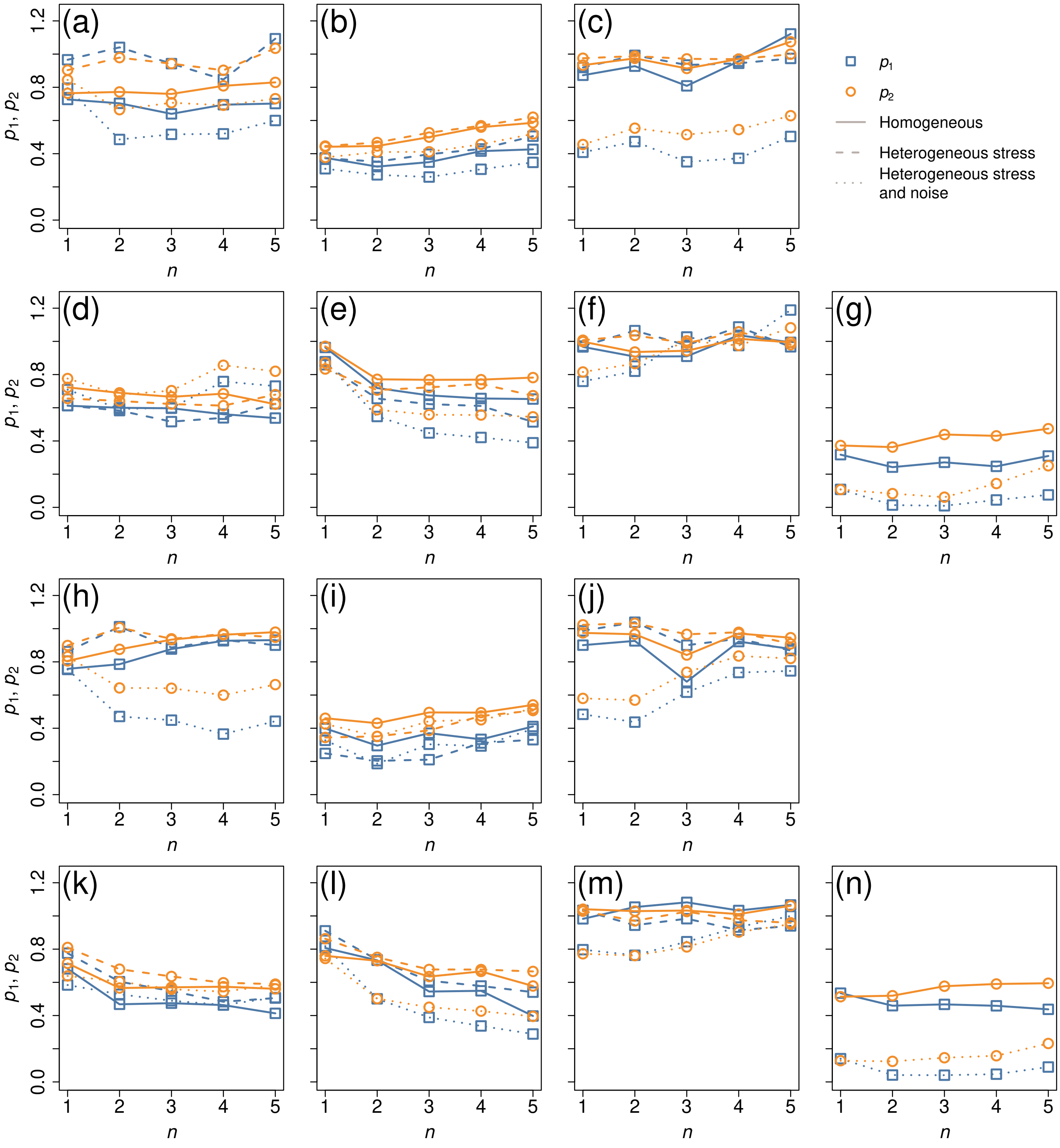

Figure S21: Performance of the node set maximizing d on the freshwater stream food web and dolphin social networks when $k^{(1)} = 0.1\tilde{K}$ and $k^{(2)} = 0.5\tilde{K}$. (a)–(g): Freshwater stream food web network. (h)–(n): Dolphin social network. (a) and (h): Double-well, u. (b) and (i): Mutualistic interaction, u. (c) and (j): Gene regulatory, u. (d) and (k): Double-well, D. (e) and (l): Mutualistic interaction, D. (f) and (m): Gene regulatory, D. (g) and (n): SIS, λ.

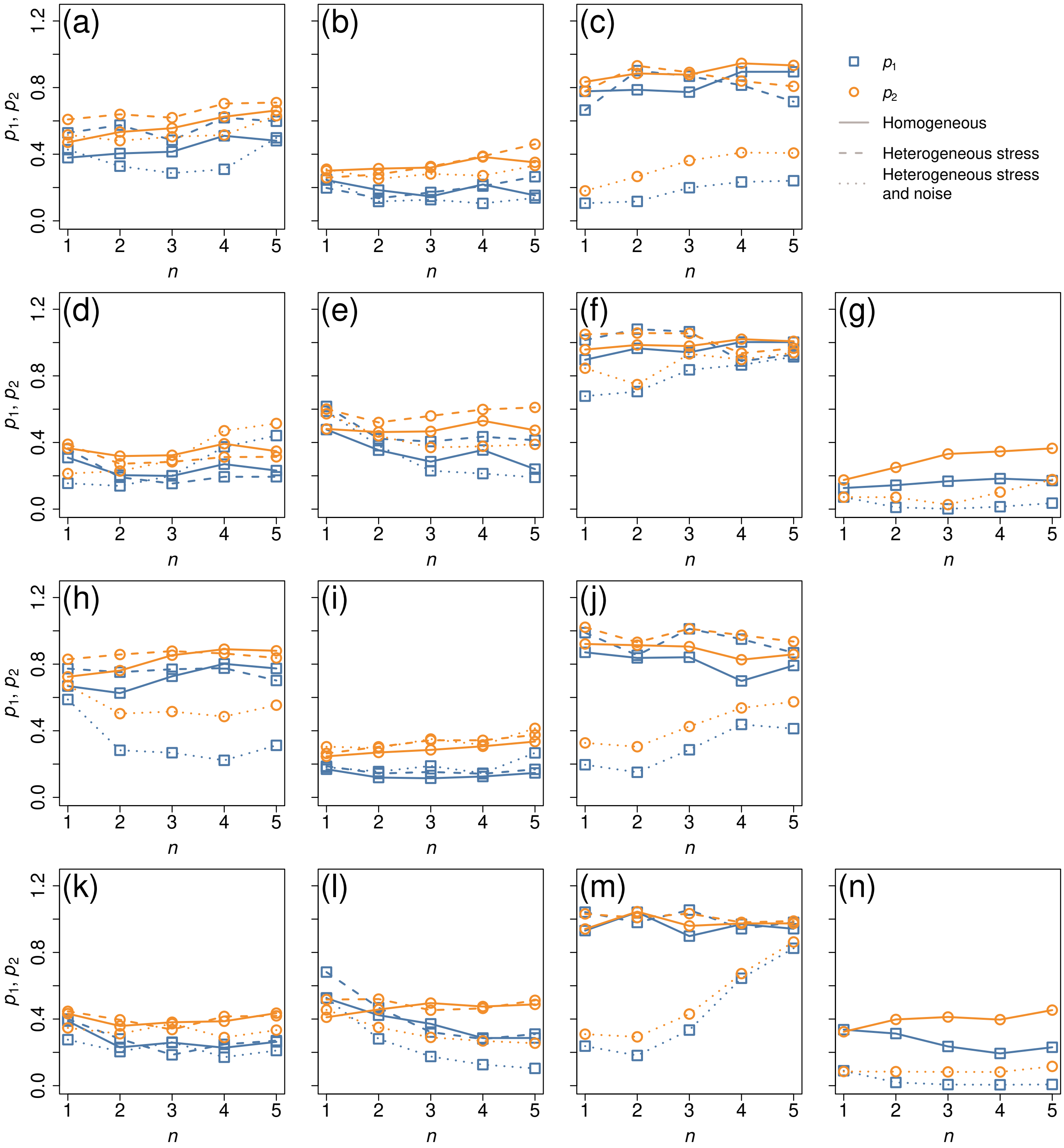

Figure S22: Performance of the node set maximizing d on the freshwater stream food web and dolphin social networks when $k^{(1)} = 0.1\tilde{K}$ and $k^{(2)} = 0.7\tilde{K}$. (a)–(g): Freshwater stream food web network. (h)–(n): Dolphin social network. (a) and (h): Double-well, u. (b) and (i): Mutualistic interaction, u. (c) and (j): Gene regulatory, u. (d) and (k): Double-well, D. (e) and (l): Mutualistic interaction, D. (f) and (m): Gene regulatory, D. (g) and (n): SIS, λ.

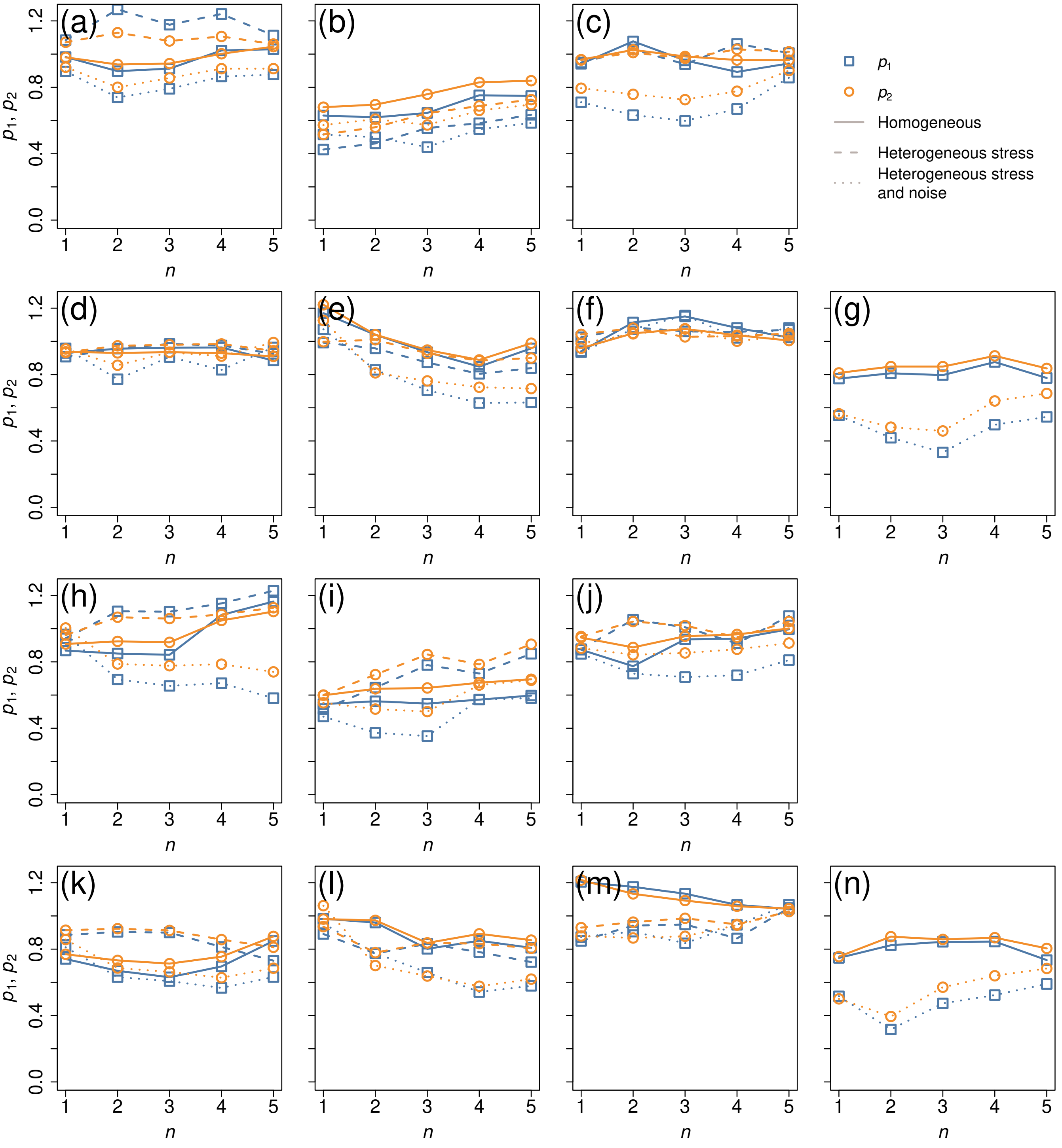

Figure S23: Performance of the node set maximizing d on the freshwater stream food web and dolphin social networks when $k^{(1)} = 0.3\tilde{K}$ and $k^{(2)} = 0.5\tilde{K}$. (a)–(g): Freshwater stream food web network. (h)–(n): Dolphin social network. (a) and (h): Double-well, u. (b) and (i): Mutualistic interaction, u. (c) and (j): Gene regulatory, u. (d) and (k): Double-well, D. (e) and (l): Mutualistic interaction, D. (f) and (m): Gene regulatory, D. (g) and (n): SIS, λ.

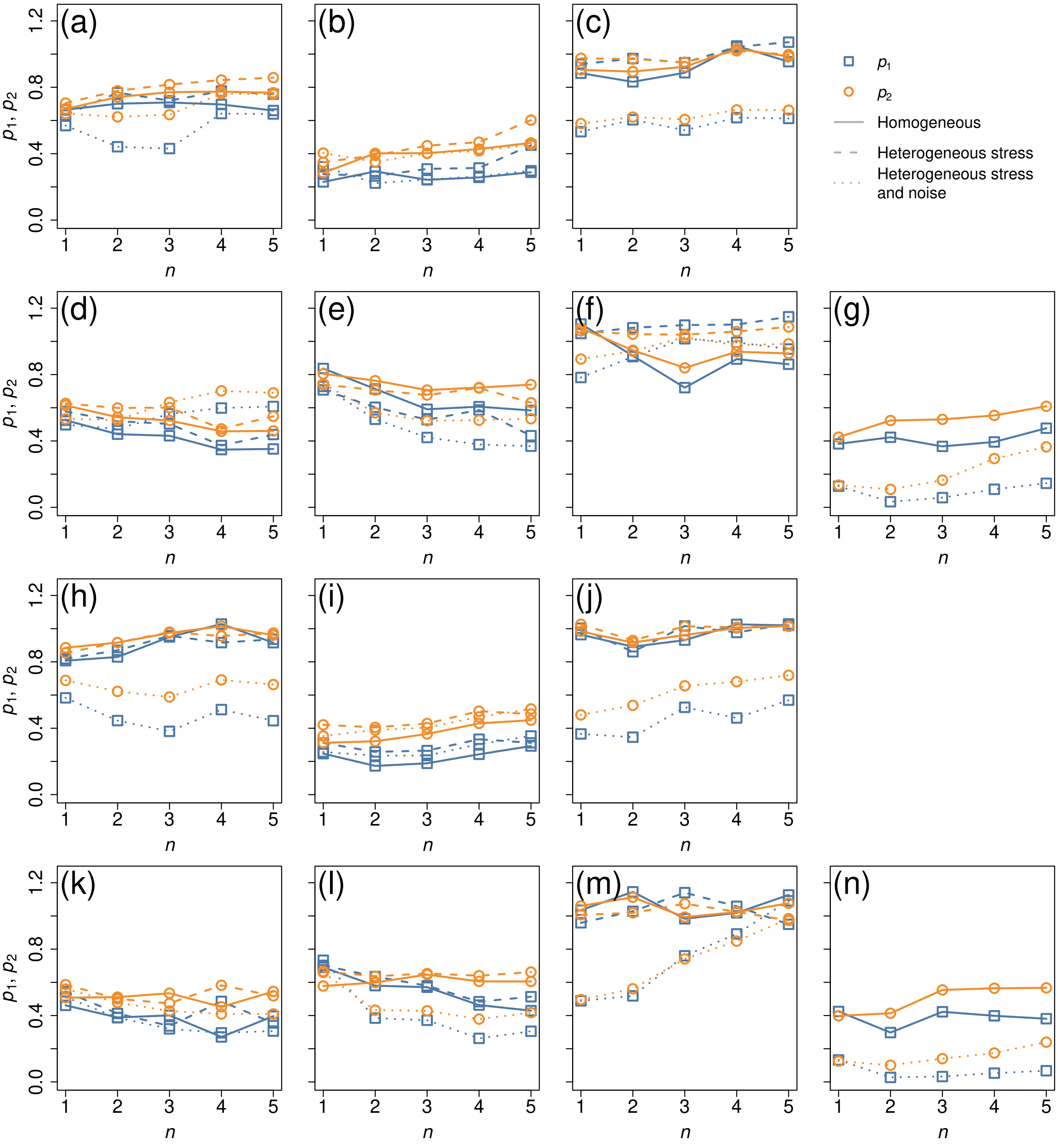

Figure S24: Performance of the node set maximizing d on the freshwater stream food web and dolphin social networks when $k^{(1)} = 0.3\tilde{K}$ and $k^{(2)} = 0.7\tilde{K}$. (a)–(g): Freshwater stream food web network. (h)–(n): Dolphin social network. (a) and (h): Double-well, u. (b) and (i): Mutualistic interaction, u. (c) and (j): Gene regulatory, u. (d) and (k): Double-well, D. (e) and (l): Mutualistic interaction, D. (f) and (m): Gene regulatory, D. (g) and (n): SIS, λ.

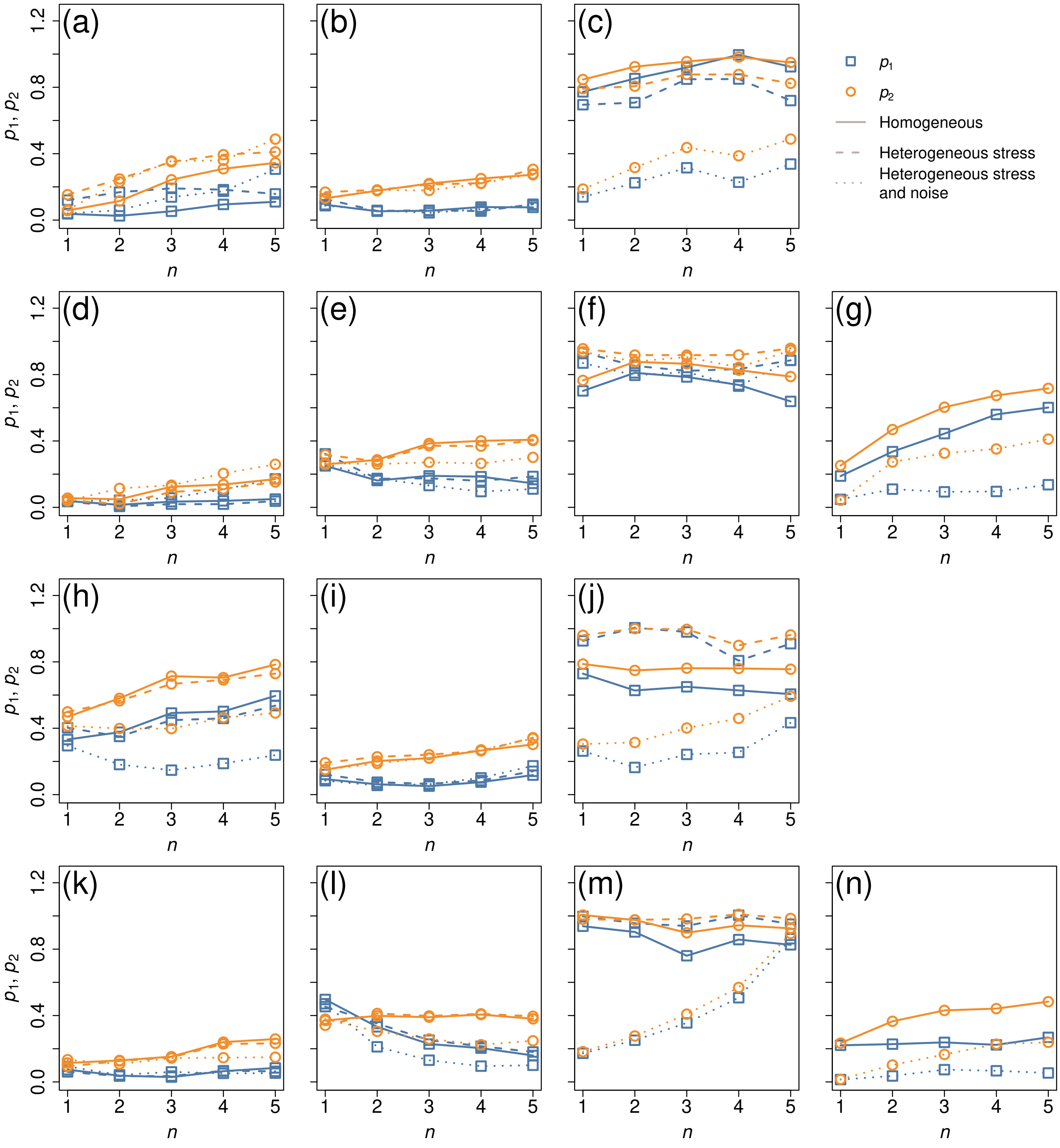

Figure S25: Performance of the node set maximizing d on the freshwater stream food web and dolphin social networks when $k^{(1)} = 0.3\tilde{K}$ and $k^{(2)} = 0.9\tilde{K}$. (a)–(g): Freshwater stream food web network. (h)–(n): Dolphin social network. (a) and (h): Double-well, u. (b) and (i): Mutualistic interaction, u. (c) and (j): Gene regulatory, u. (d) and (k): Double-well, D. (e) and (l): Mutualistic interaction, D. (f) and (m): Gene regulatory, D. (g) and (n): SIS, λ.

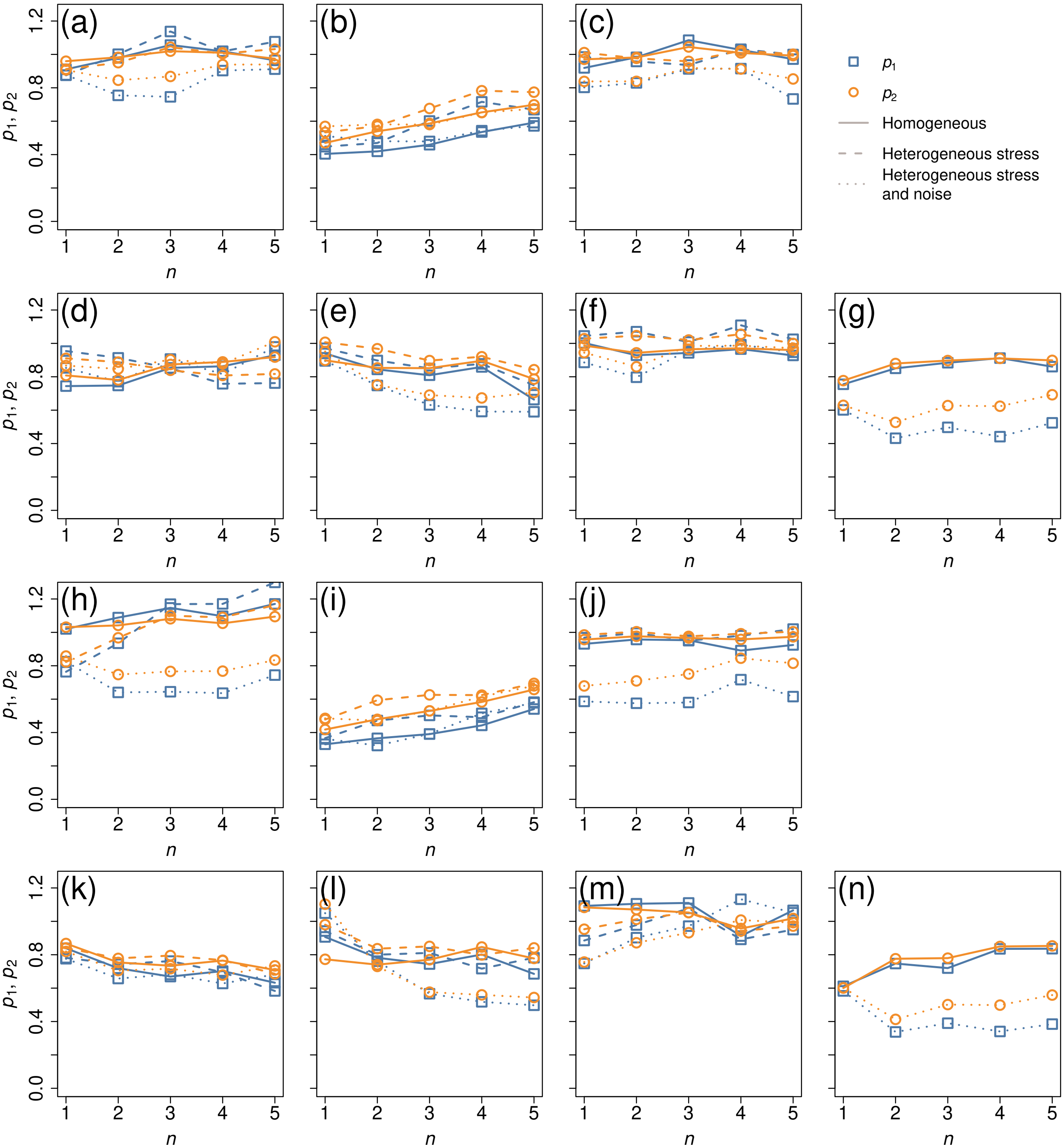

Figure S26: Performance of the node set maximizing d on the freshwater stream food web and dolphin social networks when $k^{(1)} = 0.5\tilde{K}$ and $k^{(2)} = 0.7\tilde{K}$. (a)–(g): Freshwater stream food web network. (h)–(n): Dolphin social network. (a) and (h): Double-well, u. (b) and (i): Mutualistic interaction, u. (c) and (j): Gene regulatory, u. (d) and (k): Double-well, D. (e) and (l): Mutualistic interaction, D. (f) and (m): Gene regulatory, D. (g) and (n): SIS, λ.

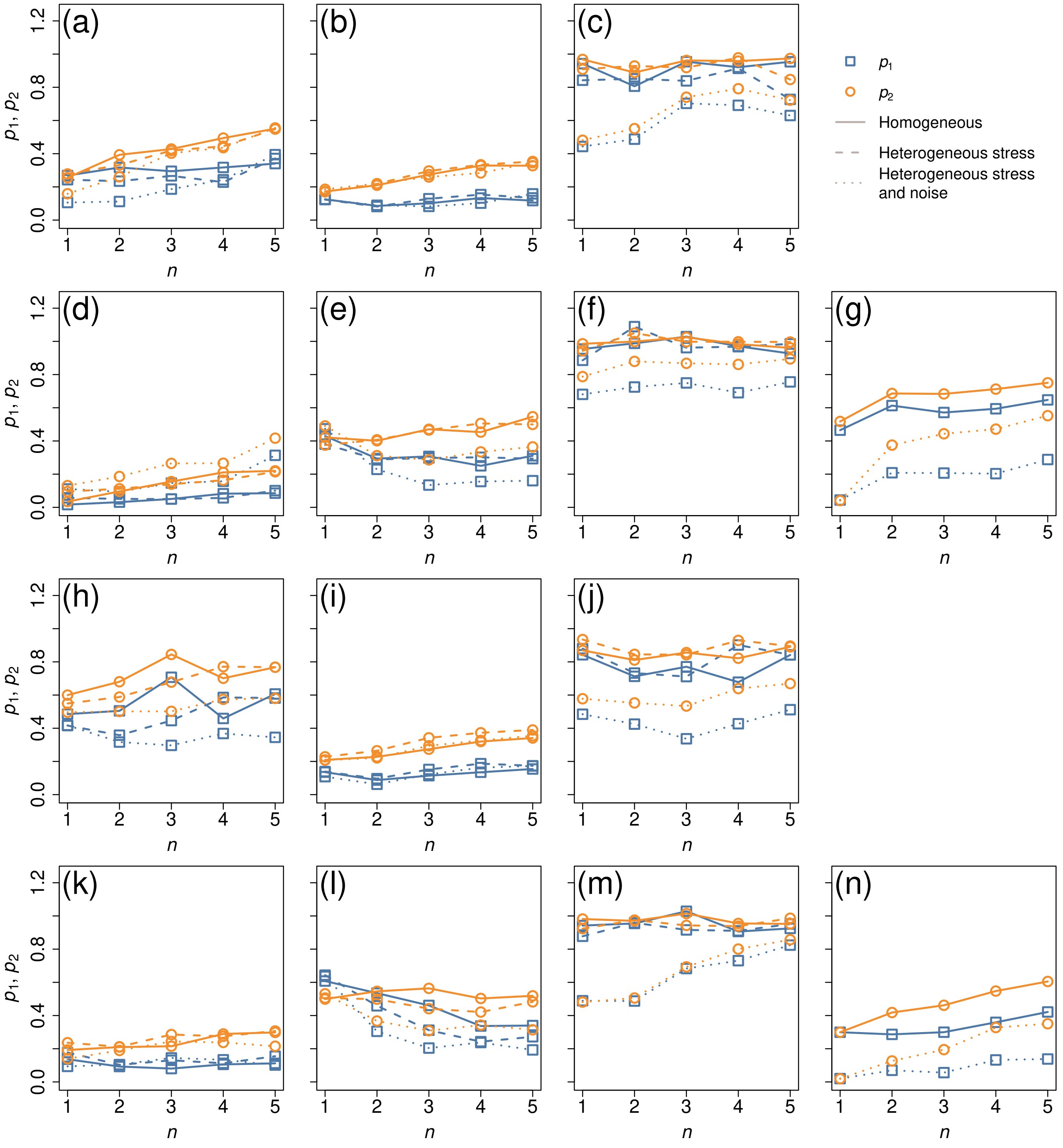

Figure S27: Performance of the node set maximizing d on the freshwater stream food web and dolphin social networks when $k^{(1)} = 0.5\tilde{K}$ and $k^{(2)} = 0.9\tilde{K}$. (a)–(g): Freshwater stream food web network. (h)–(n): Dolphin social network. (a) and (h): Double-well, u. (b) and (i): Mutualistic interaction, u. (c) and (j): Gene regulatory, u. (d) and (k): Double-well, D. (e) and (l): Mutualistic interaction, D. (f) and (m): Gene regulatory, D. (g) and (n): SIS, λ.

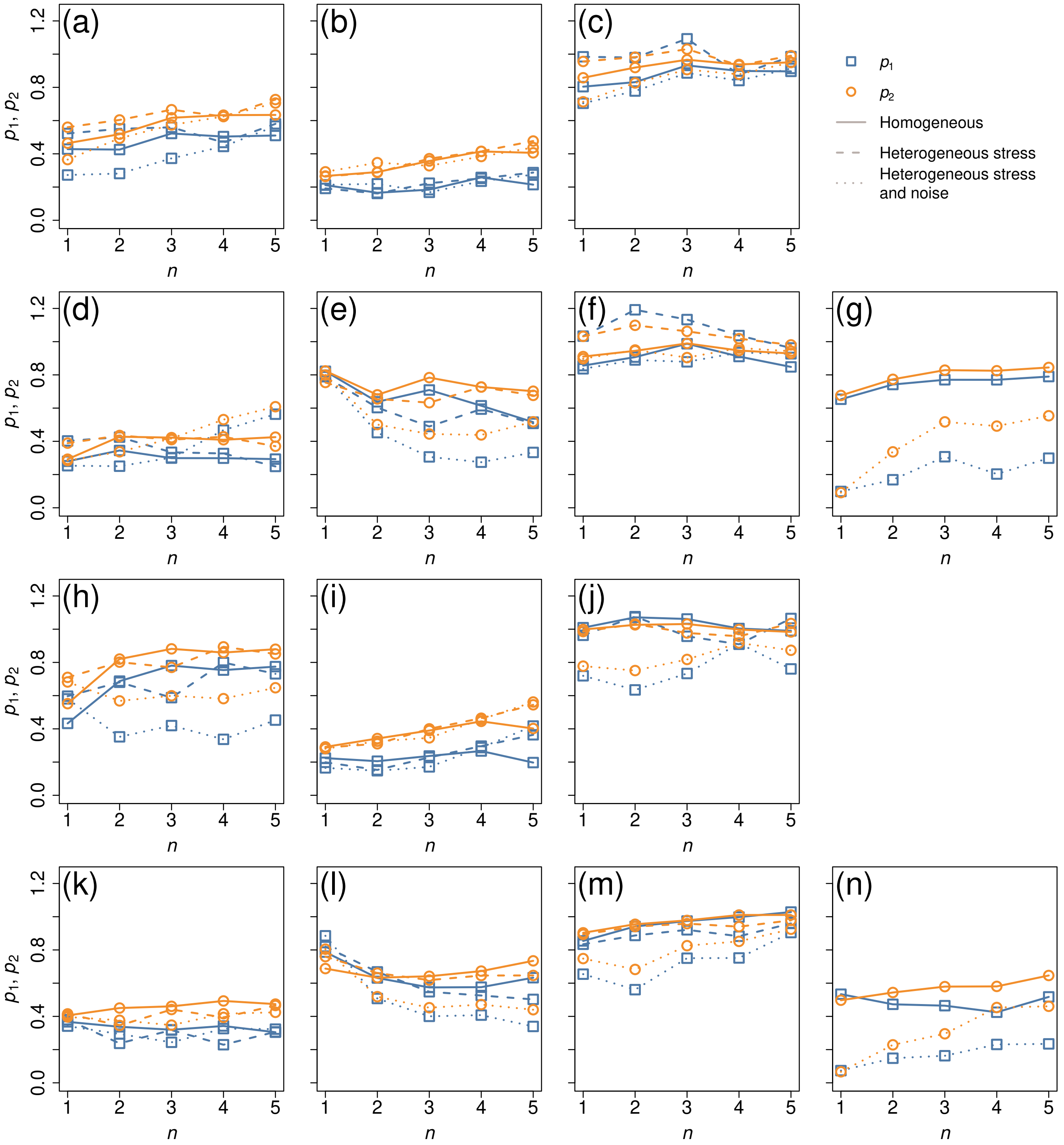

Figure S28: Performance of the node set maximizing d on the freshwater stream food web and dolphin social networks when $k^{(1)} = 0.7\tilde{K}$ and $k^{(2)} = 0.9\tilde{K}$. (a)–(g): Freshwater stream food web network. (h)–(n): Dolphin social network. (a) and (h): Double-well, u. (b) and (i): Mutualistic interaction, u. (c) and (j): Gene regulatory, u. (d) and (k): Double-well, D. (e) and (l): Mutualistic interaction, D. (f) and (m): Gene regulatory, D. (g) and (n): SIS, λ.

Supplementary Note 3: Uniqueness and stability of the solution satisfying $x_1^*, x_2^* < 0$ in the dynamical system on a chain with three nodes

We rewrite Eqs. (19) and (20) in the main text as

$$x_2 = -\frac{(x_1)^2}{w} - 1 - \frac{r}{w}, \tag{S7}$$

$$x_1 = -\frac{(x_2)^2}{2w} - 1 - \frac{r}{2w}, \tag{S8}$$

respectively, where we have omitted the asterisk in the superscript. Equations (S7) and (S8) are parabolas symmetric with respect to the x_1 and x_2 axes, respectively.

The vertex of the parabola given by Eq. (S7) is $\mathrm{V}_1 = (0, -1 - \frac{r}{w})$. This parabola intersects the x_1 axis at $\mathrm{P}_1^+ = (\sqrt{-w-r}, 0)$ and $\mathrm{P}_1^- = (-\sqrt{-w-r}, 0)$ if $-w - r \geq 0$, i.e., $r \leq -w$. Similarly, the vertex of the parabola given by Eq. (S8) is $\mathrm{V}_2 = (-1 - \frac{r}{2w}, 0)$. This parabola intersects the x_2 axis at $\mathrm{P}_2^+ = (0, \sqrt{-2w-r})$ and $\mathrm{P}_2^- = (0, -\sqrt{-2w-r})$ if $-2w - r \geq 0$, i.e., $r \leq -2w$. See Fig. S29(a) for visualization.

As r increases from -1, V_2 and P_1^+ collide on the x_1 axis before V_1 and P_2^+ collide on the x_2 axis (see Fig. S29(b)). This is because $-1 - \frac{r}{2w} < -1 - \frac{r}{w}$ and $\sqrt{-w-r} > \sqrt{-2w-r}$, which together imply that $-1 - \frac{r}{2w} - \sqrt{-w-r} < -1 - \frac{r}{w} - \sqrt{-2w-r}$. Points V_2 and P_1^+ collide when $-1 - \frac{r}{2w} - \sqrt{-w-r} = 0$ and $-1 - \frac{r}{2w} > 0$, i.e.,

$$r_\mathrm{c}' \equiv 2w\left[-(w+1) - \sqrt{w(w+1)}\right]. \tag{S9}$$

When $-1 \leq r < r_\mathrm{c}'$, there are four equilibria (x_1^*, x_2^*), each in a different quadrant (see Fig. S29(a)), which we refer to as p_1 (with $x_1^* > 0$ and $x_2^* > 0$), p_2 (with $x_1^* < 0$ and $x_2^* > 0$), p_3 (with $x_1^* < 0$ and $x_2^* < 0$), and p_4 (with $x_1^* > 0$ and $x_2^* < 0$). Therefore, the solution satisfying $x_1^*, x_2^* < 0$, i.e., p_3, is unique.

When $r = r_\mathrm{c}'$, equilibria p_1 and p_4 collide on the x_1 axis at V_2 (equivalently, P_1^+). See Fig. S29(b). As r increases slightly beyond r_c', we find that p_1 and p_4 disappear, and p_2 and p_3 remain in the second and third quadrants, respectively. As r further increases, p_2 hits the x_1 axis at V_2, which coincides with P_1^-, and this event occurs when

$$r = r_\mathrm{c}'' \equiv 2w\left[-(w+1) + \sqrt{w(w+1)}\right]. \tag{S10}$$

See Fig. S29(c). This event always occurs because V_1 moves to the negative segment of the x_2 axis at an r value larger than r_c'', i.e., at $r = -w$. It is straightforward to verify that $-w > r_\mathrm{c}''$. As r increases beyond r_c'', we find that p_2 enters the third quadrant (see Fig. S29(d)). Then, as r further increases, p_2 eventually collides with p_3 at the r value, denoted by r_c, when the two parabolas given by Eqs. (S7) and (S8) are tangent to each other. When $r > r_\mathrm{c}$, there is no equilibrium. The analytical expression of r_c is complicated but characterized in the following text.

We have shown that the equilibrium satisfying $x_1^*, x_2^* < 0$ is unique when $r \leq r_\mathrm{c}''$. However, there are two equilibria satisfying $x_1^*, x_2^* < 0$, i.e., p_2 and p_3, when $r_\mathrm{c}'' < r < r_\mathrm{c}$.

We now examine the stability of p_2 and p_3 when $x_1^*, x_2^* < 0$. Matrix A given by Eq. (21) in the main text is the negative Jacobian. Therefore, the eigenvalues of the Jacobian, denoted by λ, is given by the characteristic equation

$$\det(-A - \lambda I) = -(\lambda - x_1^*)\left[\lambda^2 - 2(x_1^* + x_2^*)\lambda + 4x_1^* x_2^* - 2w^2\right] = 0, \tag{S11}$$

where I is the 3×3 identity matrix.

One eigenvalue $\lambda_1 \equiv x_1^*$ is trivially negative because we are considering the equilibria satisfying $x_1^*, x_2^* < 0$. A necessary and sufficient condition for the other two eigenvalues, denoted by λ_2 and λ_3, to be negative is

$$\lambda_2 + \lambda_3 = 2(x_1^* + x_2^*) < 0 \tag{S12}$$

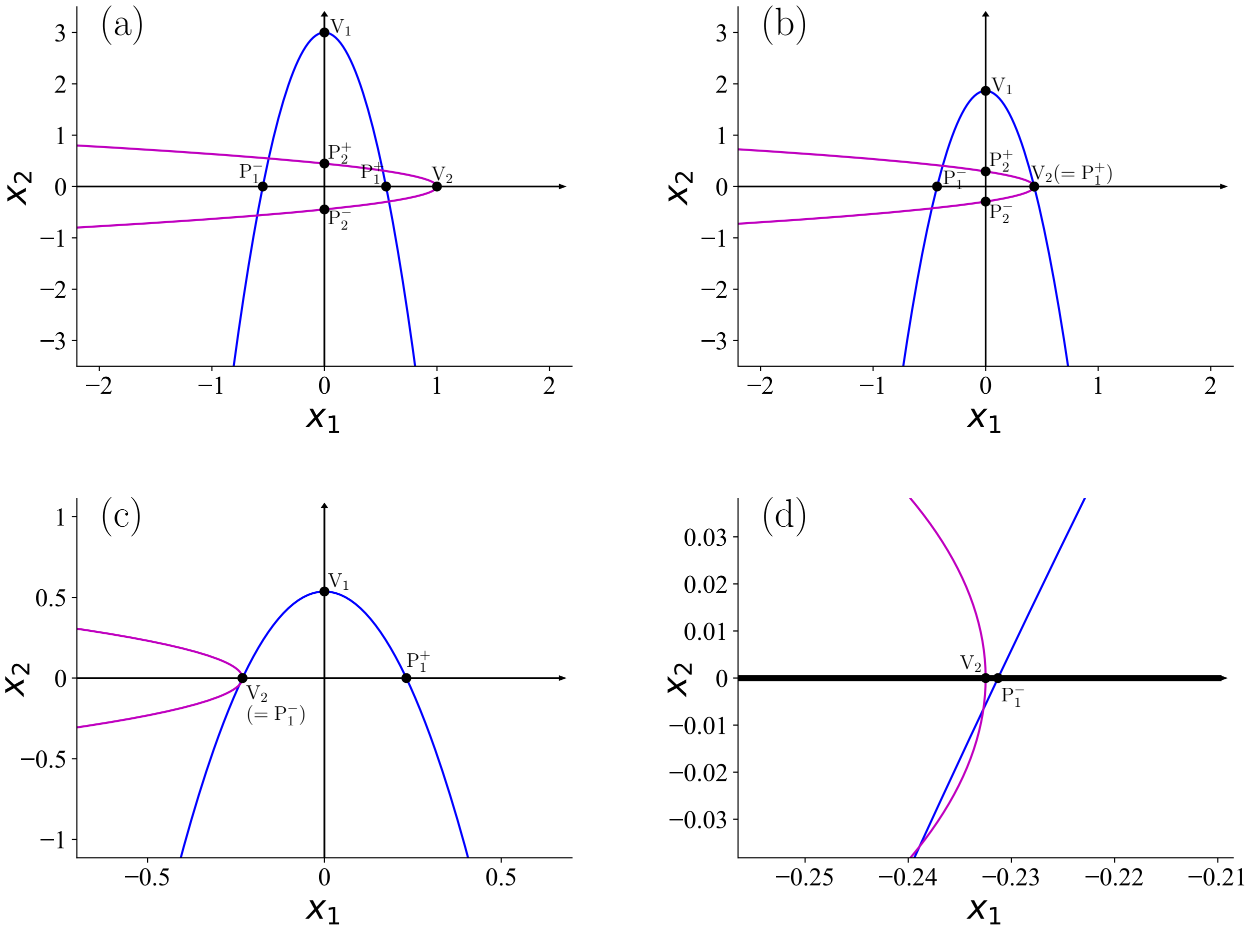

Figure S29: Two parabolas in the (x_1, x_2) plane. The blue curve represents Eq. (S7). The magenta curve represents Eq. (S8). For visualization purposes, we set $w = 0.1$. (a) $r = -0.4$. (b) $r = r'_c \approx -0.28633$. (c) $r = r''_c \approx -0.153667$. (d) $r = -0.1535$.

and

$$\lambda_2\lambda_3 = 4x_1^* x_2^* - 2w^2 > 0. \tag{S13}$$

Equation (S12) is trivially satisfied.

To examine if Eq. (S13) is also satisfied, we focus on p_3 and start by assuming that x_1^* (< 0) and x_2^* (< 0) are small enough to satisfy Eq. (S13) at $r = -1$. Otherwise, we cannot discuss the tipping points as r gradually increases. Equations (19) and (20) in the main text yield

$$1 + 2x_1^* \frac{\mathrm{d}x_1^*}{\mathrm{d}r} + w\frac{\mathrm{d}x_2^*}{\mathrm{d}r} = 0 \tag{S14}$$

and

$$1 + 2x_2^* \frac{\mathrm{d}x_2^*}{\mathrm{d}r} + 2w\frac{\mathrm{d}x_1^*}{\mathrm{d}r} = 0, \tag{S15}$$

respectively. By solving Eqs. (S14) and (S15), we obtain

$$\frac{\mathrm{d}x_1^*}{\mathrm{d}r} = \frac{-2x_2^* + w}{2(2x_1^* x_2^* - w^2)} \tag{S16}$$

and

$$\frac{\mathrm{d}x_2^*}{\mathrm{d}r} = \frac{-x_1^* + w}{2x_1^* x_2^* - w^2}. \tag{S17}$$

Equations (S16) and (S17) imply that x_1^* (< 0) and x_2^* (< 0) monotonically increase as r increases until $2x_1^*x_2^* - w^2 > 0$ is violated. Therefore, equilibrium p_3 is stable for r small enough to satisfy $2x_1^*x_2^* - w^2 > 0$.

In fact, the two parabolas given by Eqs. (S7) and (S8) are tangent to each other at the value of r satisfying $2x_1^*x_2^* = w^2$. This event necessarily occurs in the third quadrant and implies that equilibria p_2 and p_3 collide with each other at $r = r_c$, marking a saddle-node bifurcation. It is a saddle-node bifurcation because $\lambda_2 + \lambda_3 = 2(x_1^* + x_2^*) < 0$ and $\lambda_2\lambda_3 = 2(2x_1^*x_2^* - w^2) = 0$ at $r = r_c$, which together indicate that one real eigenvalue crosses the imaginary axis at $r = r_c$. To verify that the two parabolas are tangent at $r = r_c$, we use Eqs. (19) and (20) in the main text to obtain

$$\frac{\mathrm{d}x_2}{\mathrm{d}x_1} = -\frac{2x_1}{w} \tag{S18}$$

and

$$\frac{\mathrm{d}x_1}{\mathrm{d}x_2} = -\frac{x_2}{w}, \tag{S19}$$

respectively. Therefore, the two parabolas are tangent to each other when

$$\frac{\mathrm{d}x_2}{\mathrm{d}x_1} = -\frac{2x_1}{w} = -\frac{w}{x_2}, \tag{S20}$$

i.e., $2x_1^*x_2^* = w^2$.

Equilibrium p_2 also satisfies $x_1^* < 0$ and $x_2^* < 0$ when $r_c'' < r \le r_c$. However, it is unstable for the following reason. When r is slightly above r_c'', we obtain $2x_1^*x_2^* - w^2 < 0$ because x_2^* is only slightly negative. Then, Eqs. (S16) and (S17) imply that $\mathrm{d}x_1^*/\mathrm{d}r < 0$ and $\mathrm{d}x_2^*/\mathrm{d}r < 0$, respectively, such that x_1^* and x_2^* decreases as r increases. This trend continues until $2x_1^*x_2^* - w^2 = 0$ is satisfied, i.e., until r reaches r_c. Because Eq. (S13) is violated, p_2 is unstable when $r_c'' < r \le r_c$.

We have shown that p_3 is the unique stable equilibrium satisfying $x_1^*, x_2^* < 0$ and that it exists when $r < r_c$.

Supplementary Note 4: Stopping criterion for node set selection

Unless the number of nodes in the given network, N, is small, it is infeasible to find the node set S that maximizes d. Furthermore, although a larger node set S (i.e., S with large n) tends to produce a larger value of d (see Fig. 6 in the main text), monitoring signals from many nodes may be costly, which motivates us to look for approximate maximizers of d without making n too large. In the main text, we only examined $n \in \{1, 2, 3, 4, 5, N\}$. In this section, we provide a stopping criterion when we gradually increase n to look for better solutions.

Our stopping criterion is as follows. For a given n, there are $\binom{N}{n}$ choices of S, where $\binom{}{}$ denotes the binomial coefficient. Starting from $n = 1$, if $\binom{N}{n}$ is smaller than 5000, we test all S with $|S| = n$ to find the maximizer of d for that n value. Otherwise, we sample 5000 node sets with n nodes uniformly at random and find the maximizer of d. These procedures are the same as those used in the remainder of this paper. Now, as we increment n by one every time, we track how much the maximum of d realized by the best S for the given n increases. We stop increasing n when the d maximized with $|S| = n$ is not larger than 1.01 times the d maximized with $|S| = n - 1$ for the first time. In the code we have released on Github (https://github.com/ngmaclaren/mixing-EWS), we also provide a variant of this method in which we greedily optimize the node set as we increase n one by one. In other words, given the node set with n nodes realizing the largest d, denoted by $\tilde{S}_n$, we look for the maximizer of d among the node sets with $n + 1$ nodes that contain $\tilde{S}_n$. In this manner, one only needs to assess $N - n$ node sets with $n + 1$ nodes. Therefore, the optimization algorithm runs fast with the greedy algorithm although it is not guaranteed that the maximized d at each n is sufficiently close to the exact maximum.

We numerically demonstrate our node set optimization algorithm with the stopping criterion using the coupled double-well dynamics on the BA network used in Fig. 6 in the main text. We show in Fig. S30 the d and τ values as we increase n. Our algorithm stops at $n = 11$, shown by the dashed line. This is because the d value for the node set chosen at $n = 11$ is less than 1% larger than the previous d value (i.e., 0.3% larger) for the first time when we increase n from $n = 1$ one by one. Although Kendall's τ continues to increase beyond $n = 11$, the τ value at $n = 11$ (i.e., $\tau = 0.889$) is not much smaller than those at larger values of n including the τ value at $n = N = 50$. (The largest τ value for $n > 11$ is $\tau = 0.942$.) On the other hand, the τ value at $n = 11$ is notably larger than those at smaller n, particularly compared to $n \leq 7$. These results support the effectiveness of our stopping criterion, which balances the benefit of making d (and potentially τ) as large as possible and the cost of examining S with large n.

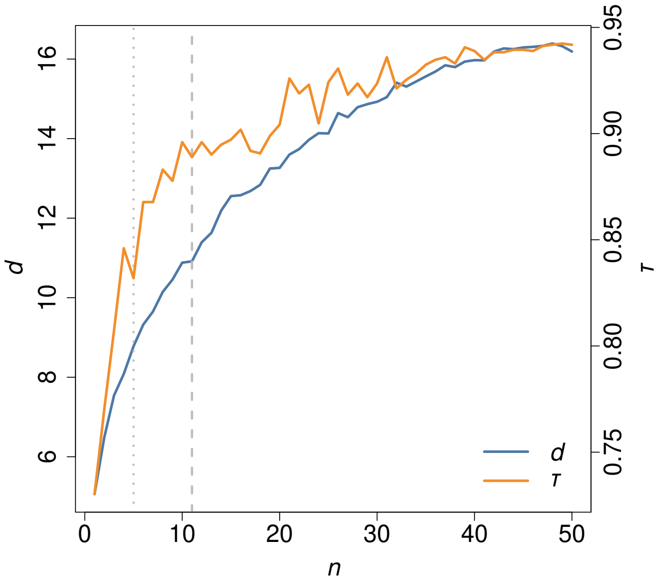

Figure S30: Demonstration of the node set optimization algorithm with the stopping criterion. We used the coupled double-well dynamics on the BA network with $N = 50$ nodes used in Fig. 6 in the main text. The dashed line indicates $n = 11$, at which the search stops. The dotted line indicates $n = 5$, which is the largest n value except $n = N$ used in the main text.

Supplementary Note 5: Definition of p_1 and p_2 when large negative τ values are better

For the mutualistic interaction and gene regulatory dynamics, x_i transits from the upper to the lower state as the bifurcation parameter gradually changes. Therefore, larger negative τ values are better. We defined p_1 and p_2, i.e., the performance of the node set maximizing d, for this case by applying the definition of p_1 and p_2 for $-\tau$ instead of τ, roughly speaking. Precisely, for a given n, we define p_1 as twice the fraction of node sets whose τ is smaller (i.e., more negative) than that for the maximizer of d. We define p_2 as $(\tau^* - \tau_{\min})/(\langle\tau\rangle - \tau_{\min})$, where $\tau_{\min}$ is the smallest (i.e., the most negative, which means the best) τ value among the node sets with n nodes examined; we recall that $\langle\tau\rangle$ is the average of τ over all the node sets with n nodes examined and that τ^* is the τ value realized by the maximizer of d. If the maximizer of d is the best node set, realizing the smallest τ, then both p_1 and p_2 are equal to 0 and the smallest. A small p_1 or p_2 value indicates that the maximizer of d provides a relatively good early warning signal.

Supplementary Note 6: Performance of the maximizer of d on the other four networks

We show in Fig. S31 the p_1 and p_2 values for the Erdős-Rényi network, node fitness network, freshwater stream food web network, and dolphin social network. The results are similar to those for the BA network and the Chesapeake Bay carbon flow network shown in Fig. 7 in the main text.

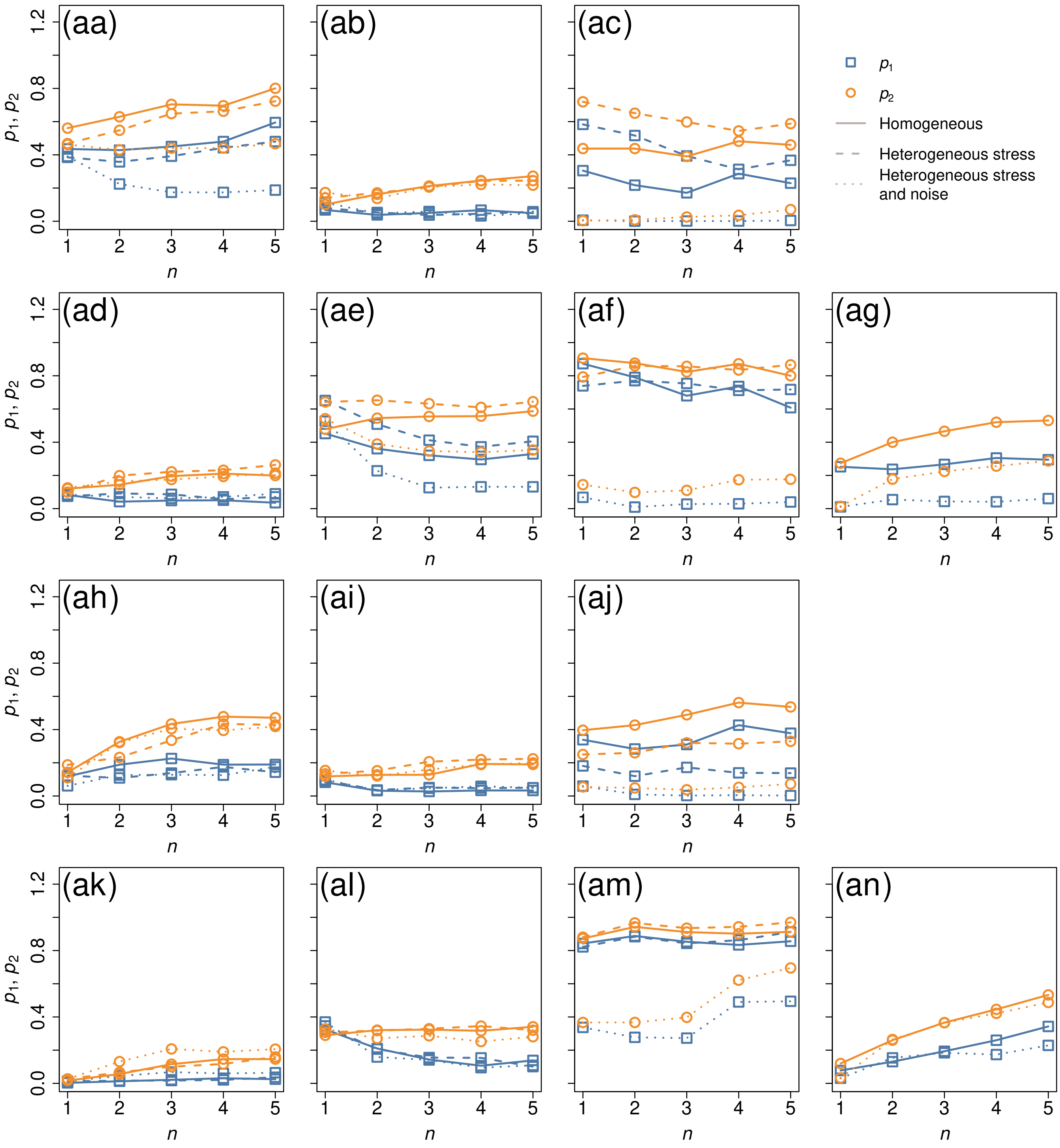

Figure S31: Performance of the node set maximizing d for four networks. The squares and circles represent p_1 and p_2, respectively, for the given n, dynamics, network, and condition (i.e., whether u_i or σ_i is homogeneously or heterogeneously distributed) averaged over 50 series of simulations. (aa)–(ag): A network generated by the Erdős-Rényi random graph. (ah)–(an): A network generated by the node fitness model. (ba)–(bg): Freshwater stream food web network. (bh)–(bn): Dolphin social network. The panels on the leftmost column correspond to the double-well dynamics, and the second to the fourth columns to the mutualistic interaction, gene regulatory, and SIS dynamics, respectively. The combination of the dynamics model and bifurcation parameter is as follows. (aa), (ah), (ba), and (bh): Double-well, u. (ab), (ai), (bb), and (bi): Mutualistic interaction, u. (ac), (aj), (bc), and (bj): Gene regulatory, u. (ad), (ak), (bd), and (bk): Double-well, D. (ae), (al), (be), and (bl): Mutualistic interaction, D. (af), (am), (bf), (bm): Gene regulatory, D. (ag), (an), (bg), and (bn): SIS, λ.

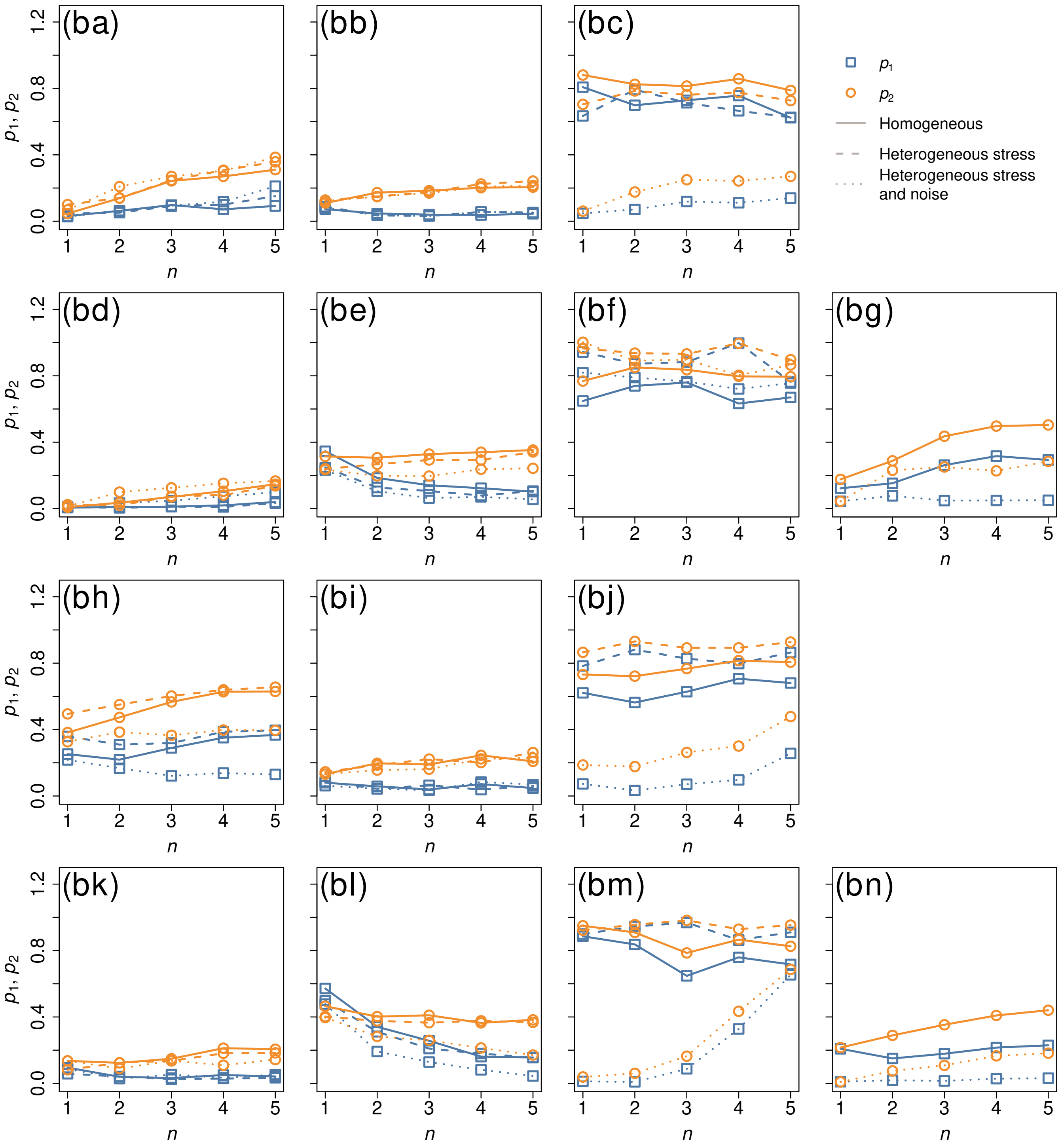

(Fig. S31 continued)

Supplementary Note 7: Regime shifts without critical slowing down

To show the behavior of $\hat{V}_S$ when regime shifts occur without critical slowing down, we simulated the coupled double-well dynamics on a BA network in the following two situations. We used the same BA network with $N = 50$ nodes and initial condition as those used in the main text.

First, dynamical noise may induce transitions from the lower to the upper states of x_i without critical slowing down. To investigate this scenario, we poise the dynamical system close to the saddle-node bifurcation point by setting $u = 0$, $D = 0.05$, and $\sigma = 0.094$. For simplicity, we set $\Delta u_i = 0$ and $\sigma_i = 0$ for all $i \in \{1, \ldots, N\}$. We also set $r_2 = 2$ to make the attractive basin of the lower state smaller than that of the upper state. This is because, with $r_2 = 2$, a transition from the lower to the upper state driven by dynamical noise, which we focus on here, occurs more easily than a transition from the upper to the lower state. The other parameter values are the same as those used in the main text. Then, we run a simulation until the first node, denoted by i_0, transits from its lower state (i.e., $x_{i_0}(t) \leq 2$) to the upper state (i.e., $x_{i_0}(t) > 2$). To calculate Kendall's τ and the node set S maximizing d, we need a sequence of sample covariance matrices. In the analysis shown in the main text, we sampled covariance matrices at every value of the bifurcation parameter. However, in the present scenario, the bifurcation and other parameters of the model are fixed throughout the simulation. Therefore, we take $\tilde{K} = 70$ consecutive time windows of length 100 TUs backward in time starting from the time at which the first node has transited from its lower state to the upper state. In each time window, we collect $L = 100$ evenly spaced samples from each $x_i(t)$, $i \in \{1, \ldots, N\}$ such that the adjacent samples are 1 TU apart. This sampling scheme within each time window is the same as that used in the main text. Then, we calculate the covariance matrix in each of the $\tilde{K}$ time windows. We use the round$(0.1\tilde{K})$th and round$(0.9\tilde{K})$th covariance matrices to identify the S maximizing d. We also use the $\tilde{K}$ covariance matrices to calculate τ by pretending that the kth time window (with $k \in \{1, \ldots, \tilde{K}\}$) is the kth smallest value of a bifurcation parameter. We emphasize that there is in fact no bifurcation parameter varying towards the saddle-node bifurcation in this simulation.

We show in Fig. S32(a) a time course of $\hat{V}_S$ for the maximizer of d, that of $\hat{V}_S$ with $S = \{i_0\}$, i.e., the single node that transits from its lower to the upper state first, and $x_i(t)$ for each i. We find that $\hat{V}_S$ is not responsive to the impending regime shift for both the maximizer of S and the i_0th node until the transition is about to occur. The relationship between τ and d for all the node sets with $n \in \{1, 2, 3, 4, 5, N\}$, shown in Fig. S32(b), indicates that τ is close to 0 for any node set S and that the maximizer of d does not particularly generate a good early warning signal in terms of τ.

To examine a second scenario of regime shifts without critical slowing down, we use the same parameter values as those in the last simulations except that we set $r_2 = 3$ and $\sigma = 0.05$, which are the values used in the main text. Now we add impulse input to all nodes by suddenly increasing u from $u = 0$ to $u = 5$ at $t = 10^4$ TU. Because $u = 5$ is large, all $x_i(t)$ values almost suddenly approach and cross the threshold ($= 2$; see Fig. 4 of [6] for a schematic), realizing a regime shift without notable critical slowing down. Then, we compute d and τ in the same manner as in the first scenario.

We show time courses of $\hat{V}_S$ for the maximizer of d and for $S = \{i_0\}$, and $x_i(t)$ $\forall i \in \{1, \ldots, N\}$ in Fig. S32(c). We show the relationships between τ and d for all node sets with $n \in \{1, 2, 3, 4, 5, N\}$ in Fig. S32(d). The results are similar to those shown in Figs. S32(a) and (b). We conclude that our early warning signals, $\hat{V}_S$, do not anticipate regime shifts that do not accompany critical slowing down, at least under these two scenarios.

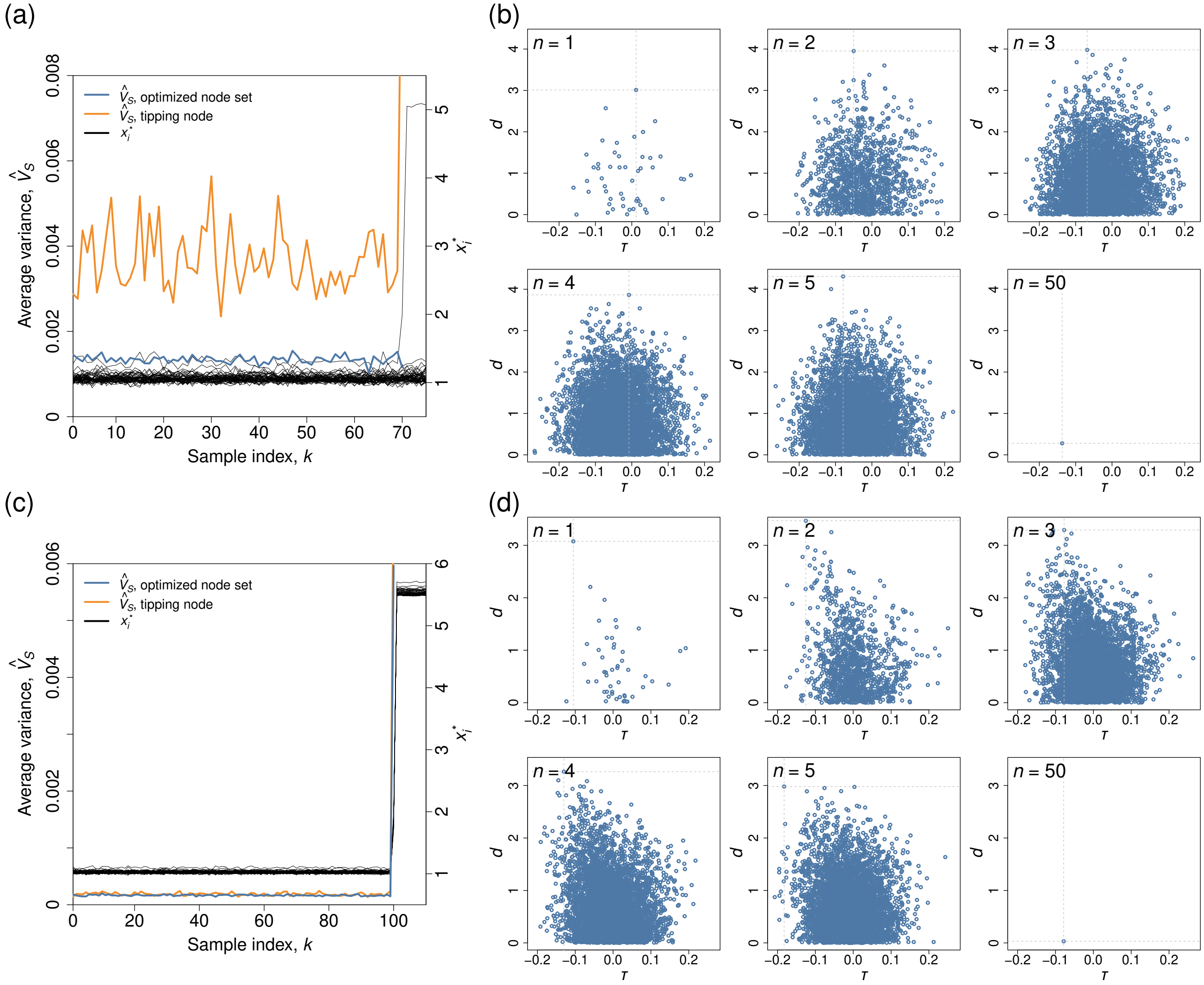

Figure S32: Early warning signals when regime shifts occur without critical slowing down. (a) $\hat{V}_S$ and $x_i(t)$ when dynamical noise induces a regime shift. (b) Relationships between τ and d when dynamical noise induces a regime shift. (c) $\hat{V}_S$ and $x_i(t)$ when impulse input induces a regime shift. (d) Relationships between τ and d when impulse input induces a regime shift. In (a) and (c), we only show one $x_i(t)$ value per TU for each i to prevent the plots from being too congested. In (b) and (d), each circle represents a node set, and the τ and d values for the node set maximizing d for each n value are highlighted by the dashed lines. We used the coupled double-well dynamics on the BA network with $N = 50$ nodes.

Supplementary Note 8: Comparison of the maximizer of d and Large SD

In Figs. S33, S34, S35, S36, S37, and S38, we compare the Kendall's τ values obtained with the maximizer of d (plotted on the vertical axis) and those obtained with Large SD (plotted on the horizontal axis) for the BA network, ER random graph, node fitness network, Chesapeake Bay network, freshwater stream food web network, and dolphin social network, respectively. We tested the four models of dynamics and the three heterogeneity conditions for each network.

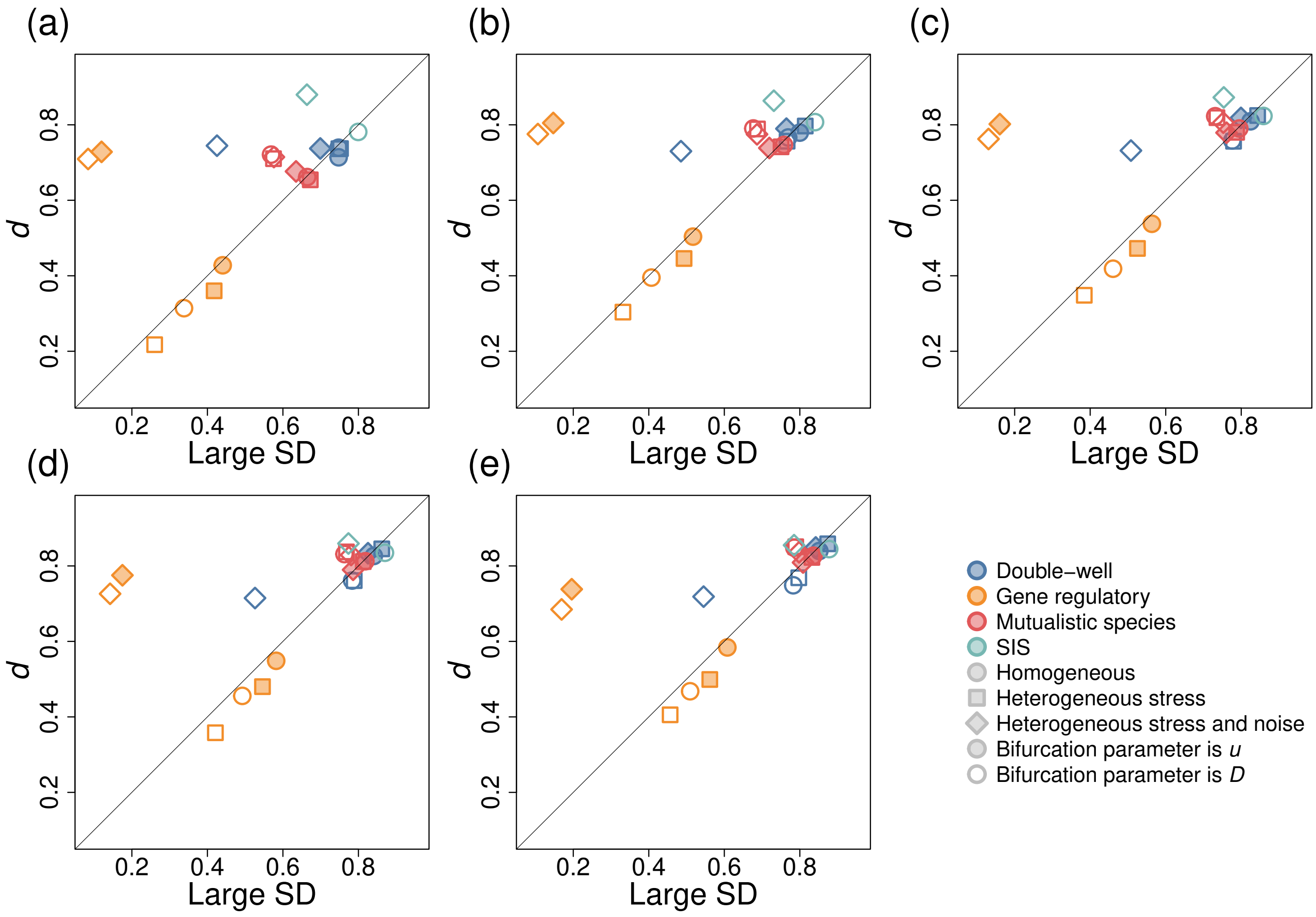

Figure S33: Relationships between the Kendall's τ values for the maximizer of d and those for Large SD on the BA network. (a) $n = 1$. (b) $n = 2$. (c) $n = 3$. (d) $n = 4$. (e) $n = 5$. The marker color identifies the dynamics model. The marker shape identifies the numerical simulation condition: the circles indicate the homogeneous stress and noise case; the squares indicate the heterogeneous stress and homogeneous noise case; the diamonds indicate the heterogeneous stress and noise case. The filled and open markers correspond to the numerical simulations in which the bifurcation parameter is u and D, respectively. These conventions are indicated by the legends. Because we do not introduce or vary the stress in the SIS dynamics, for the SIS dynamics, there are no squares, and the diamonds indicate the heterogeneous noise case. Each marker shows the average τ value over 50 series of simulations. The solid lines represent the diagonals.

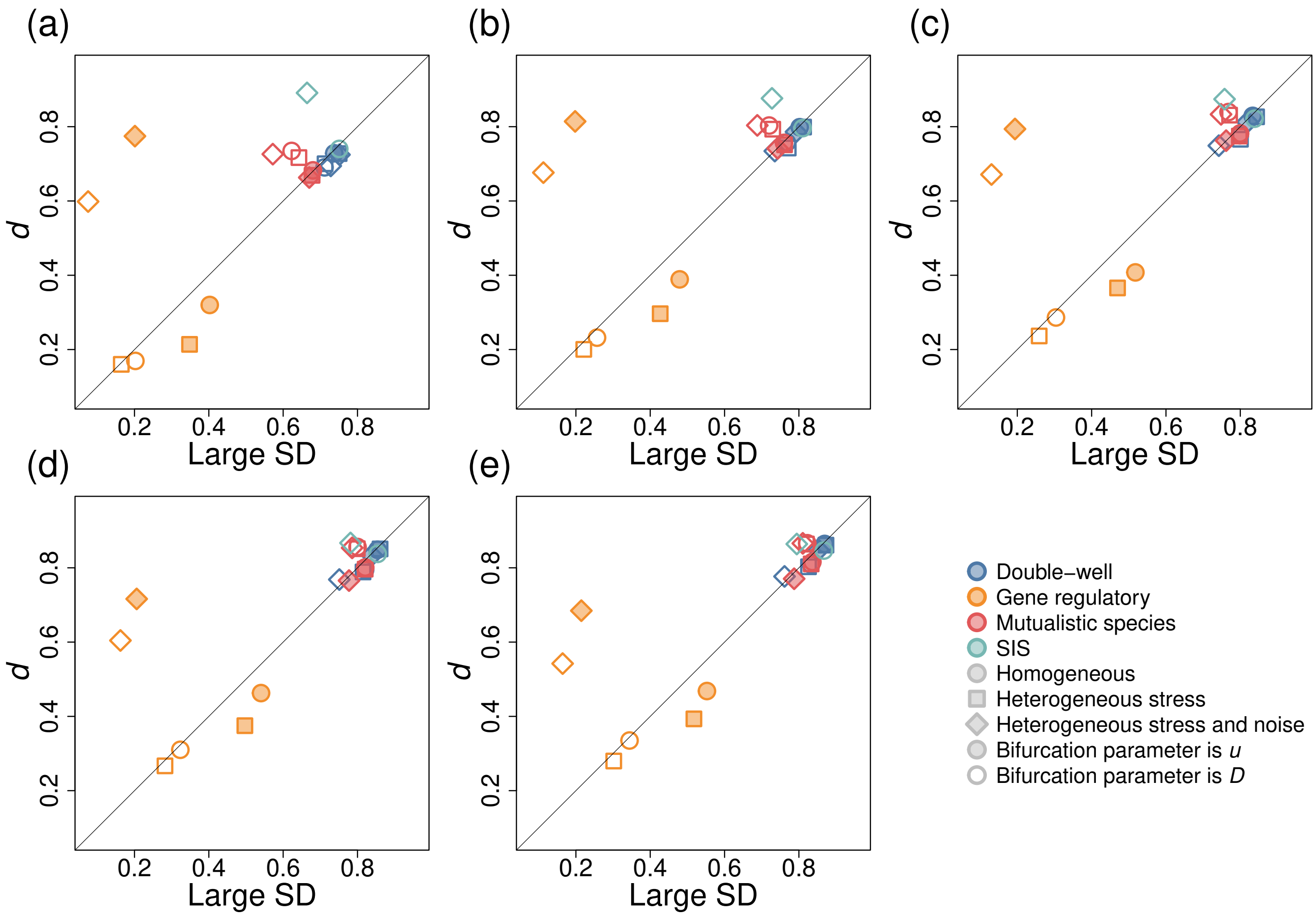

Figure S34: Relationships between the Kendall's τ values for the maximizer of d and those for Large SD on the ER random graph. (a) $n = 1$. (b) $n = 2$. (c) $n = 3$. (d) $n = 4$. (e) $n = 5$. See the legends of Fig. S33 for the detailed explanation of the marker conventions.

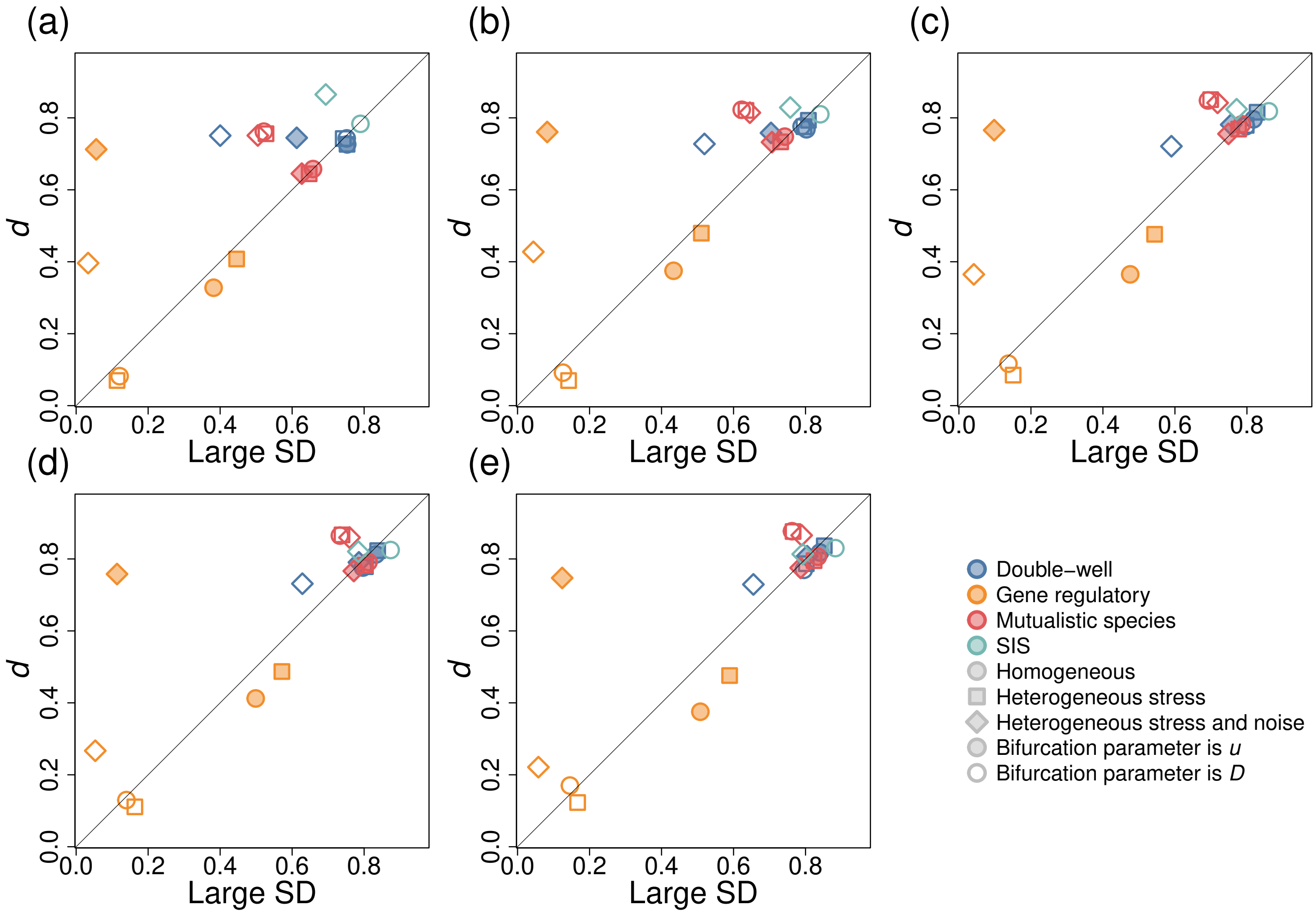

Figure S35: Relationships between the Kendall's τ values for the maximizer of d and Large SD on the network generated by the node fitness network. (a) $n = 1$. (b) $n = 2$. (c) $n = 3$. (d) $n = 4$. (e) $n = 5$.

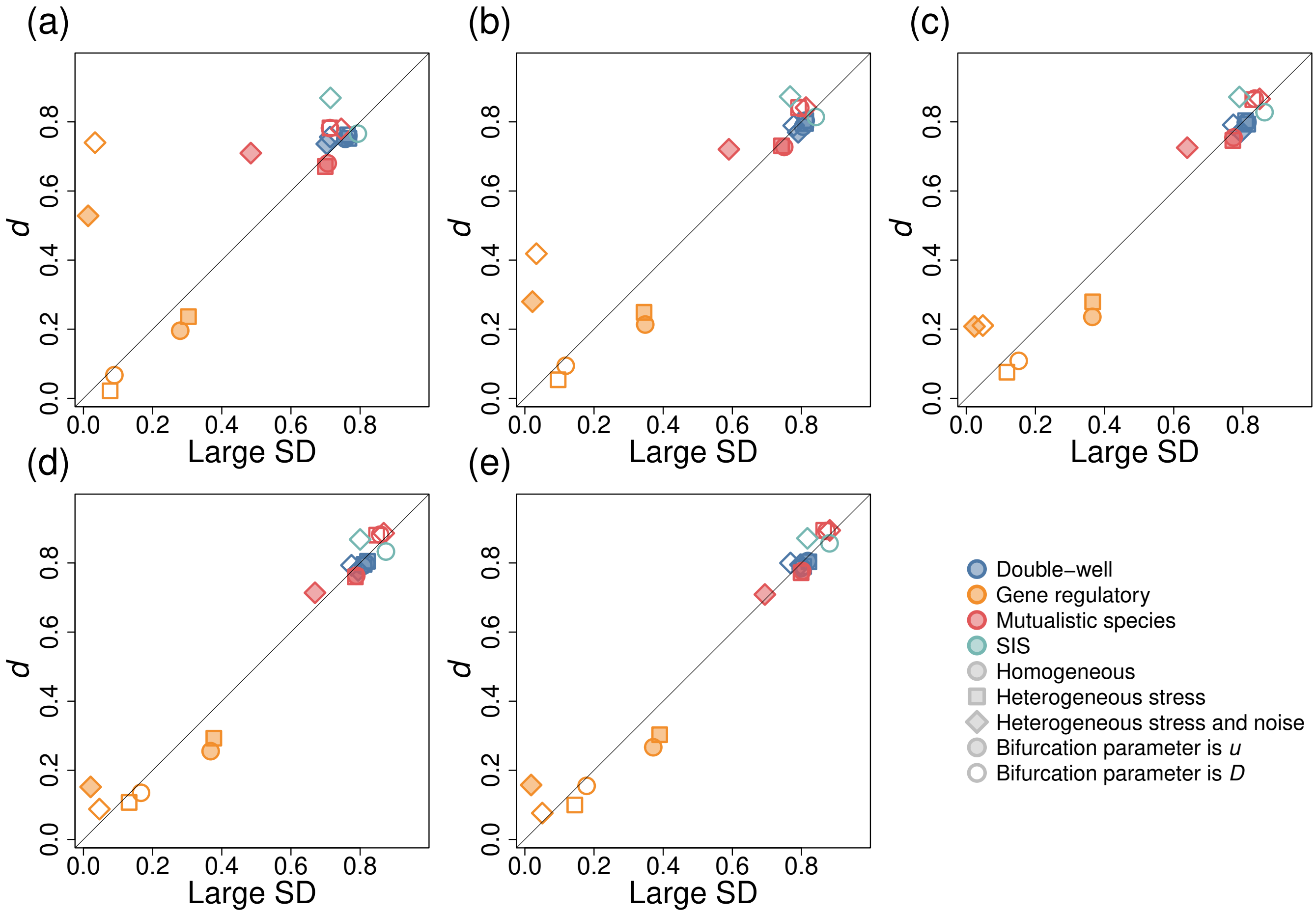

Figure S36: Relationships between the Kendall's τ values for the maximizer of d and Large SD on the Chesapeake Bay network. (a) $n = 1$. (b) $n = 2$. (c) $n = 3$. (d) $n = 4$. (e) $n = 5$.

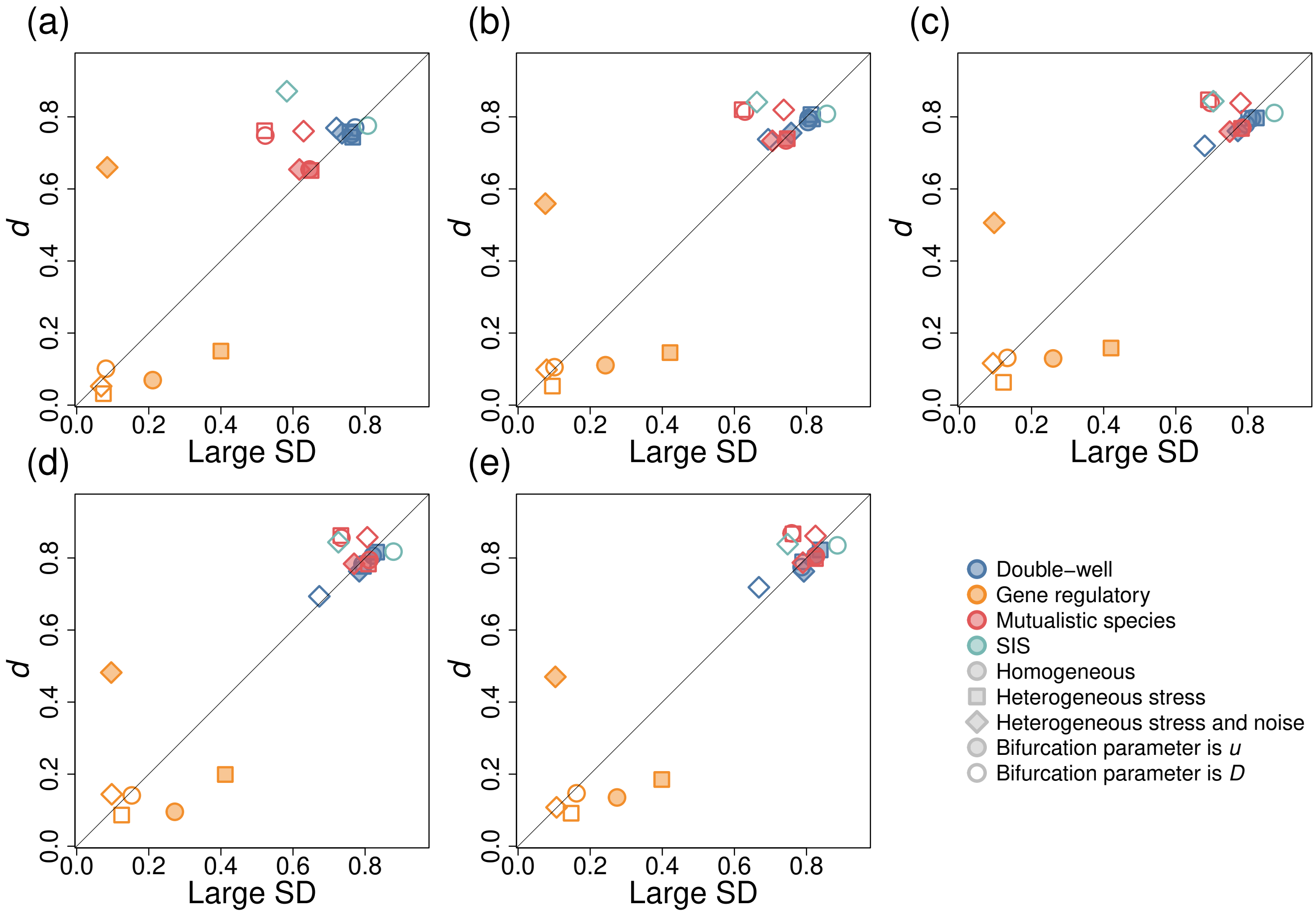

Figure S37: Relationships between the Kendall's τ values for the maximizer of d and Large SD on the freshwater stream food web network. (a) $n = 1$. (b) $n = 2$. (c) $n = 3$. (d) $n = 4$. (e) $n = 5$.

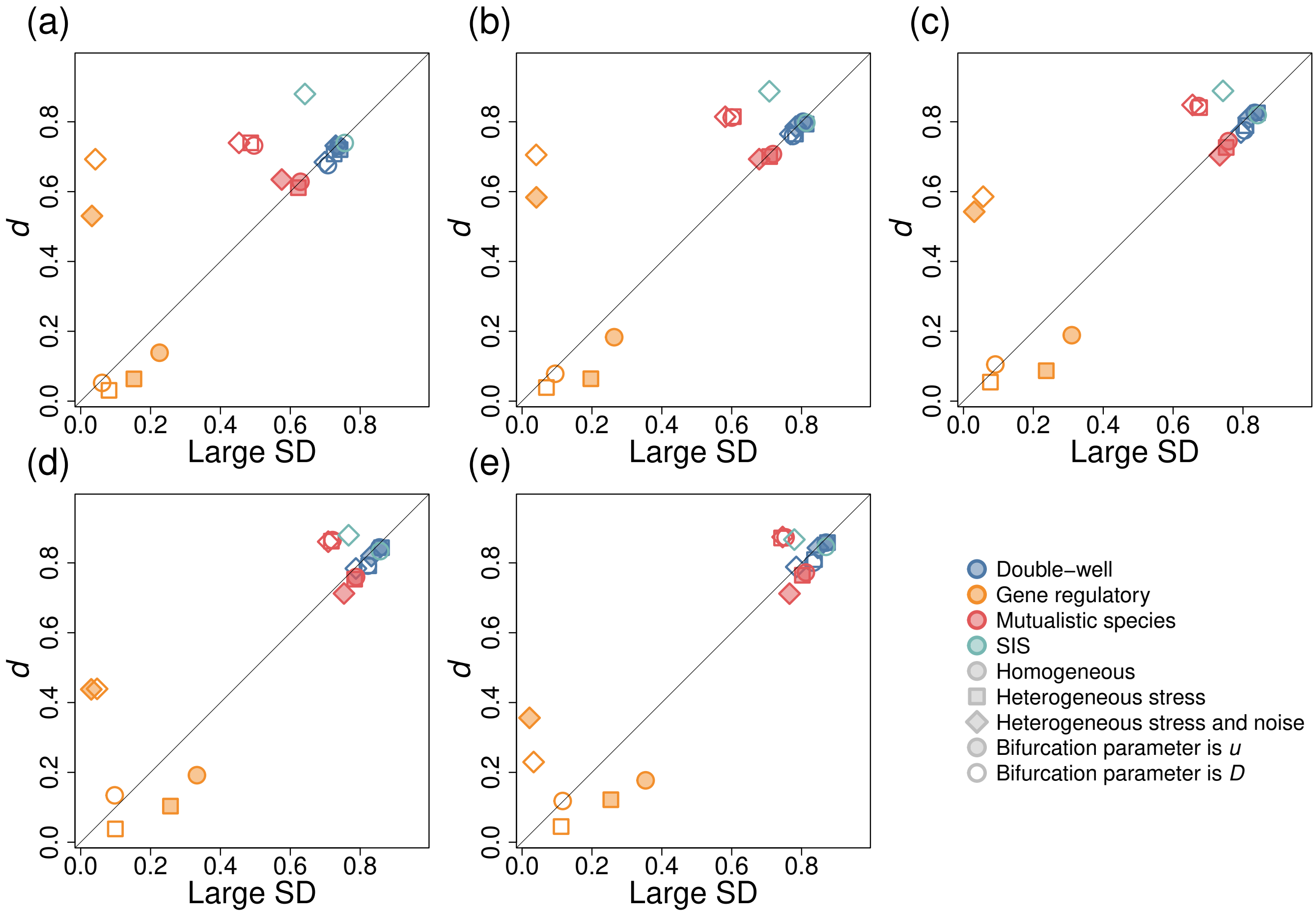

Figure S38: Relationships between the Kendall's τ values for the maximizer of d and Large SD on the dolphin social network. (a) $n = 1$. (b) $n = 2$. (c) $n = 3$. (d) $n = 4$. (e) $n = 5$.

Supplementary Note 9: Performance of the maximizer of d when the early warning signal is the standard deviation of $x_i(t)$ averaged over the selected nodes

We show in Fig. S39 the p_1 and p_2 values for the four dynamics on the BA network and the Chesapeake Bay network when the early warning signal is the average of the standard deviation rather than the variance of $x_i(t)$ over the n nodes in node set S selected by maximization of d. The results are almost the same as those when the early warning signal is the variance of $x_i(t)$, which are shown in Fig. 8 in the main text.

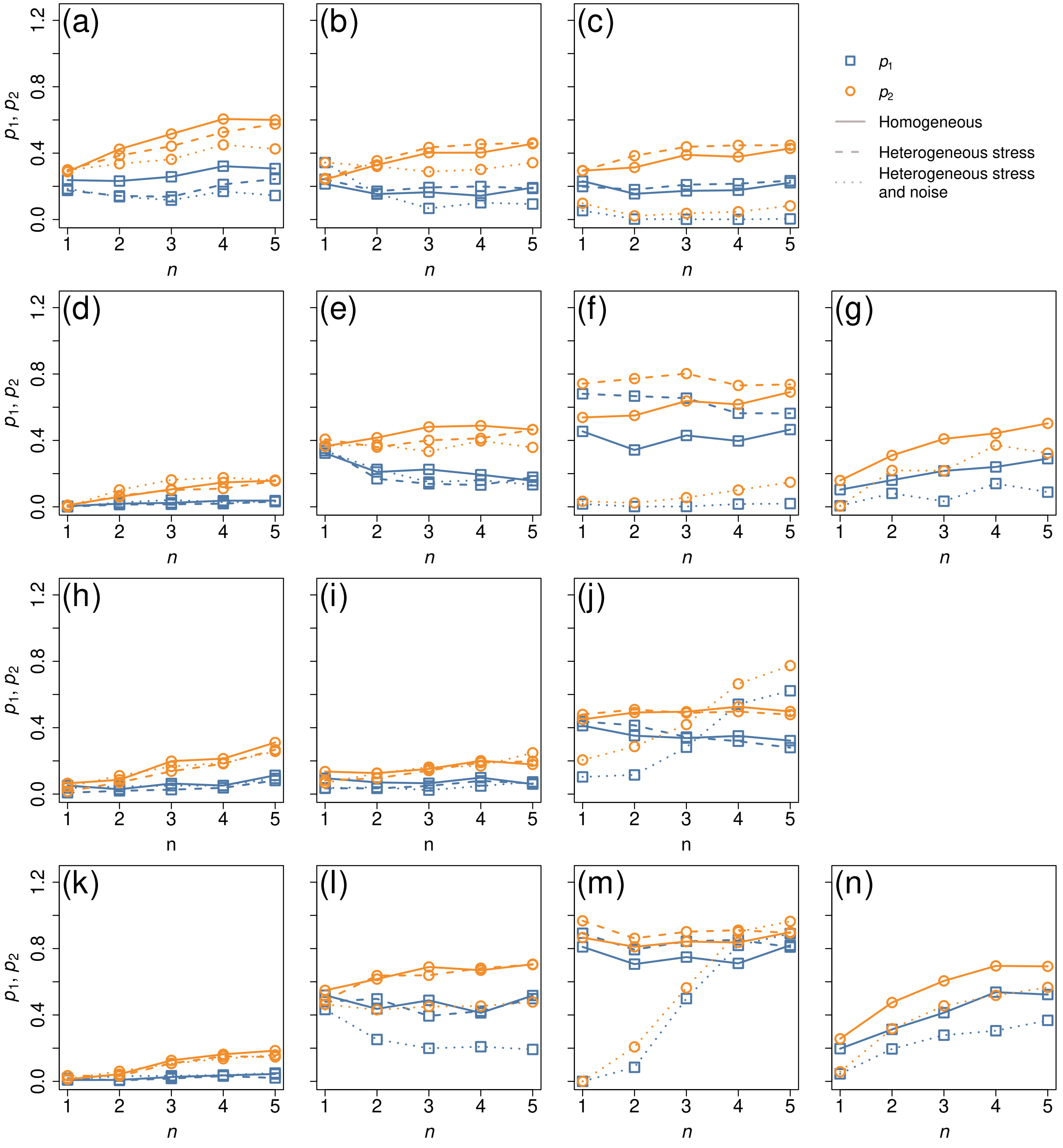

Figure S39: Performance of the node set S maximizing d quantified by p_1 and p_2 when the early warning signal is the standard deviation of $x_i(t)$ averaged over the n nodes in S. As in Fig. 7, the squares and circles represent p_1 and p_2, respectively, for the given n, dynamics, network, and condition (i.e., whether u_i or σ_i is homogeneously or heterogeneously distributed) averaged over 50 series of simulations. (A)–(G): BA network. (H)–(N): Chesapeake Bay network. The combination of the dynamics model and bifurcation parameter is (A) and (H): double-well, u, (B) and (I): mutualistic interaction, u, (C) and (J): gene regulatory, u, (D) and (K): double-well, D, (E) and (L): mutualistic interaction, D, (F) and (M): gene regulatory, D, and (G) and (N): SIS, λ.

Supplementary Note 10: Comparison with the High Input and Low Input algorithms

In the High Input algorithm, we select the n nodes with the largest value of $R_i = \sum_{j=1}^{N} w_{ij}\overline{x}_j$, where $\overline{x}_j$ is the average of $x_i(t)$ calculated over the L evenly spaced samples after the transient, which we described in detail in the Methods section of the main text. This quantity is the sum of the input from all the other nodes if the coupling is linear, as in the case of the coupled double-well dynamics (see Eq. (26) in the main text). In the Low Input algorithm, we select the n nodes with the smallest R_i values. In a previous study, early warning signals calculated from the nodes selected by High Input were effective at anticipating transitions of the nodes from their lower to upper state; likewise those calculated with Low Input were effective at anticipating transitions from their upper to lower states [7]. Therefore, we calculated the Kendall's τ with the High Input node set for the double-well and SIS dynamics, for which we gradually changed the bifurcation parameters to induce the transitions from the lower to upper states. Similarly, we calculated τ with the Low Input node set for the mutualistic interaction and gene regulatory dynamics.

We compare the Kendall's τ values obtained with the maximizer of d and those obtained with High/Low Input in Figs. S40, S41, S42, S43, S44, and S45 for the BA network, ER random graph, node fitness network, Chesapeake Bay network, freshwater stream food web network, and dolphin social network, respectively. The results are similar to those for the comparison between the maximizer of d and Large SD shown in Fig. 9 in the main text and section Supplementary Note 8:.

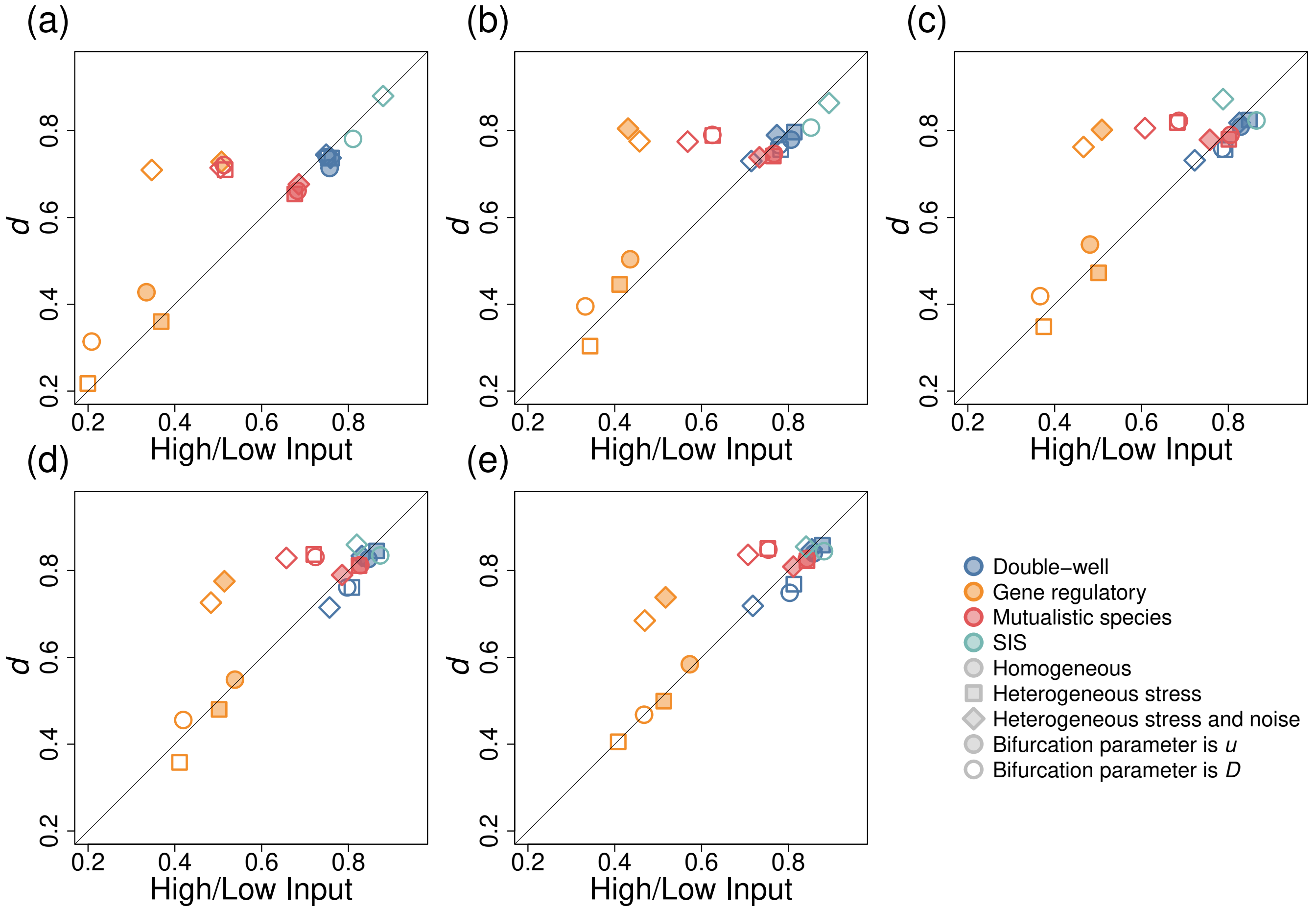

Figure S40: Relationships between the Kendall's τ values for the maximizer of d and those for High/Low Input on the BA network. (a) $n = 1$. (b) $n = 2$. (c) $n = 3$. (d) $n = 4$. (e) $n = 5$. See the legends of Fig. S33 for the detailed explanation of the marker conventions. Each marker shows the average τ value over 50 independent series of simulations. The solid lines represent the diagonals.

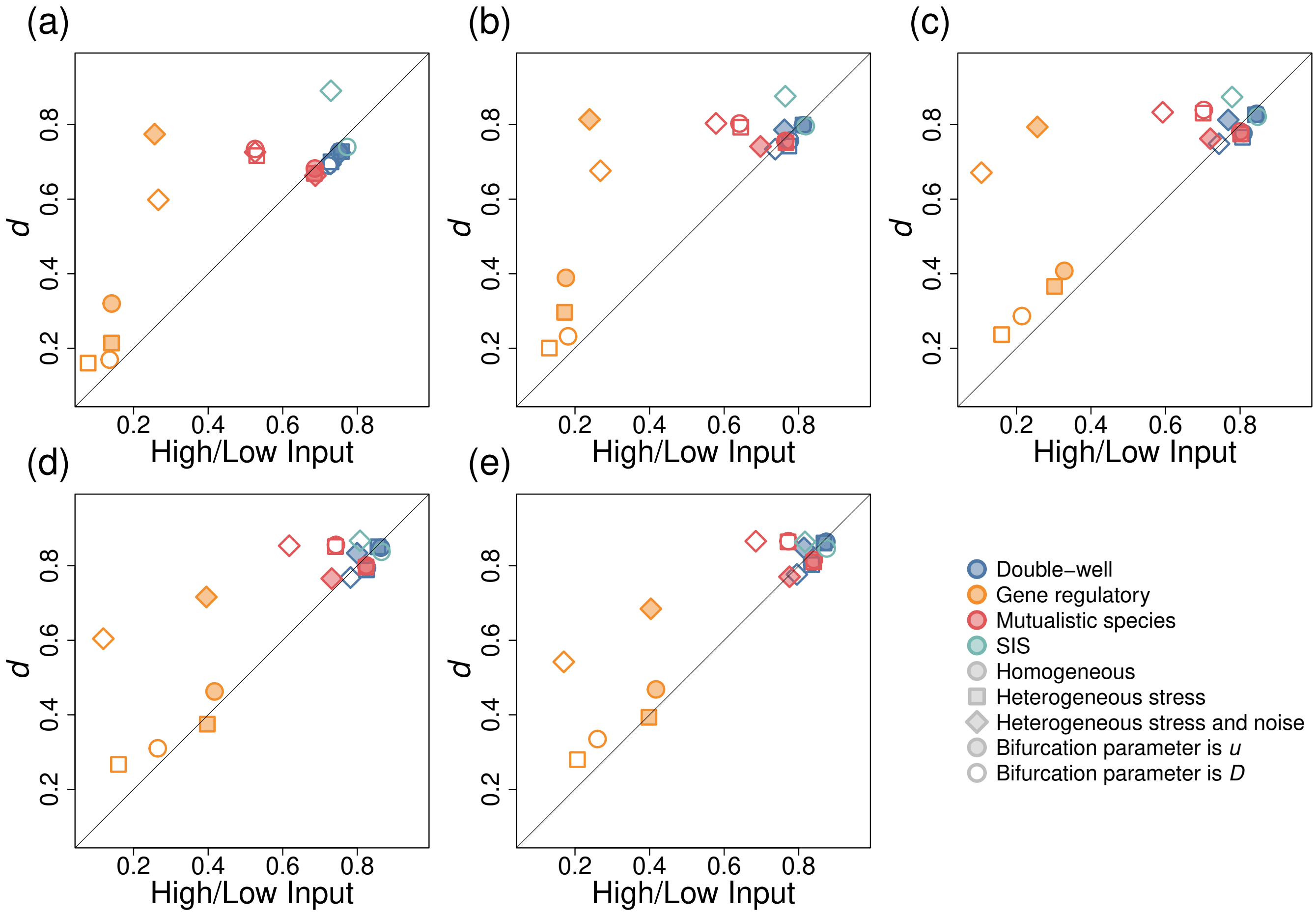

Figure S41: Relationships between the Kendall's τ values for the maximizer of d and those for High/Low Input on the ER random graph. (a) $n = 1$. (b) $n = 2$. (c) $n = 3$. (d) $n = 4$. (e) $n = 5$.

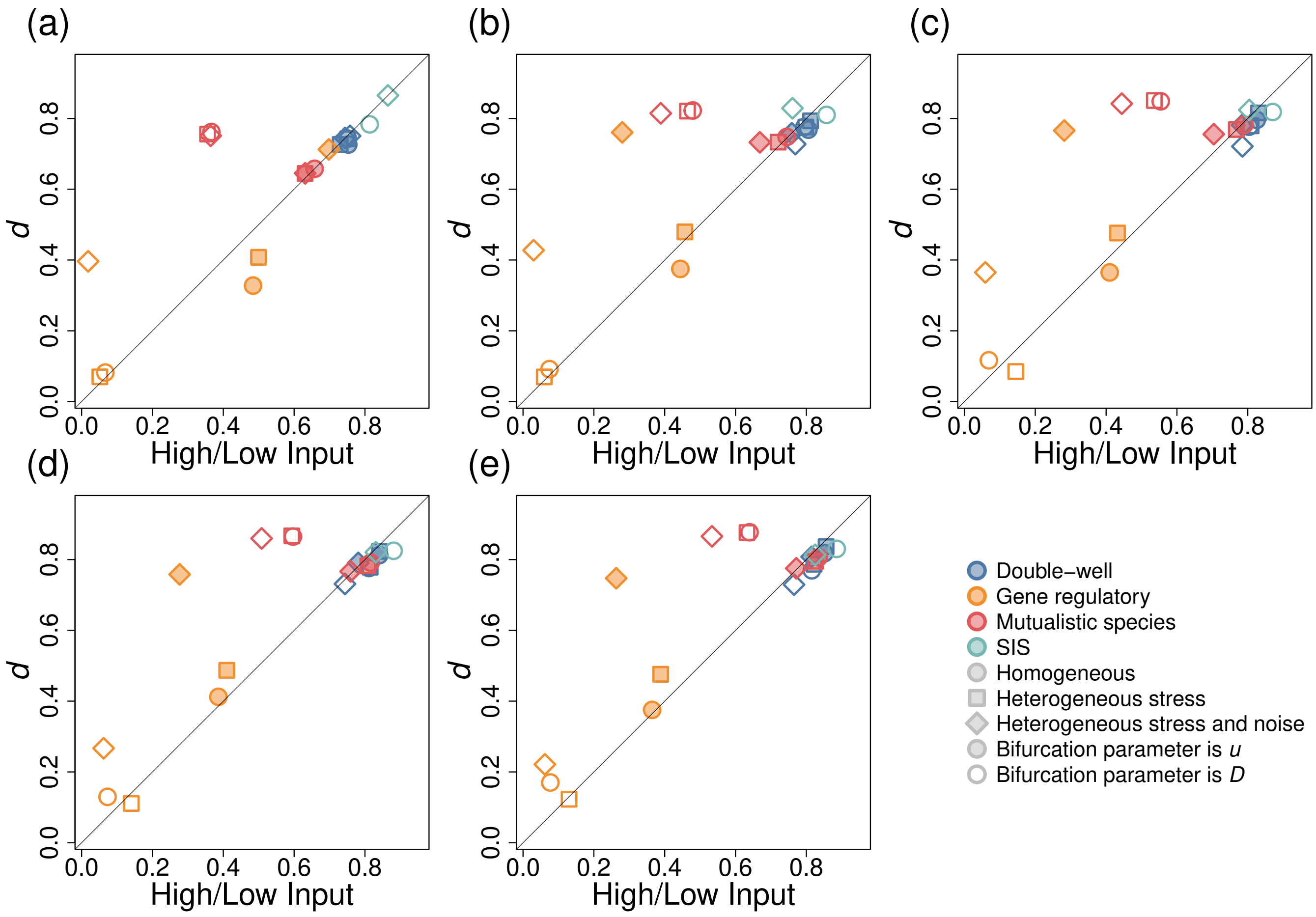

Figure S42: Relationships between the Kendall's τ values for the maximizer of d and those for High/Low Input on the network generated by the node fitness network. (a) $n = 1$. (b) $n = 2$. (c) $n = 3$. (d) $n = 4$. (e) $n = 5$.

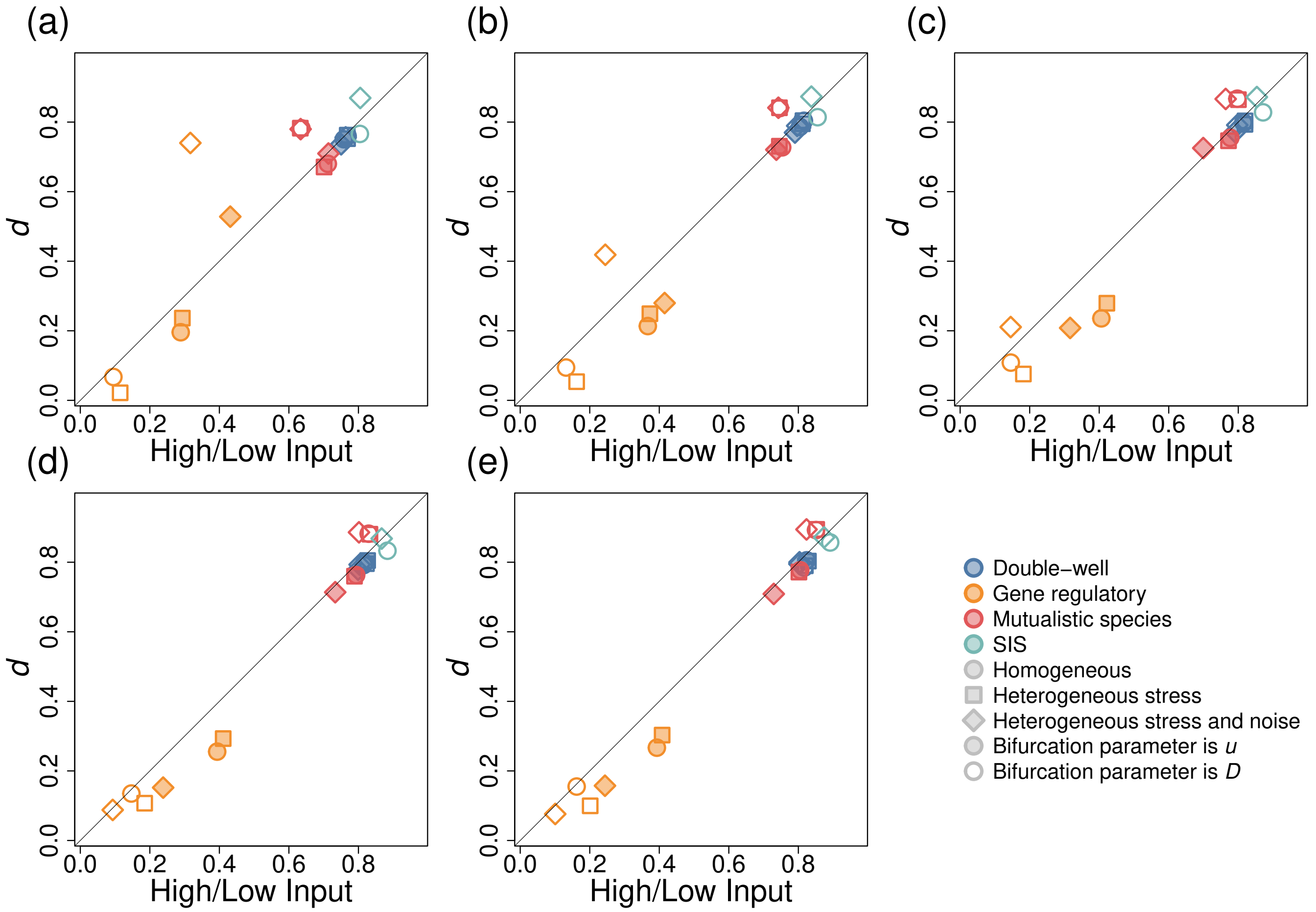

Figure S43: Relationships between the Kendall's τ values for the maximizer of d and those for High/Low Input on the Chesapeake Bay network. (a) $n = 1$. (b) $n = 2$. (c) $n = 3$. (d) $n = 4$. (e) $n = 5$.

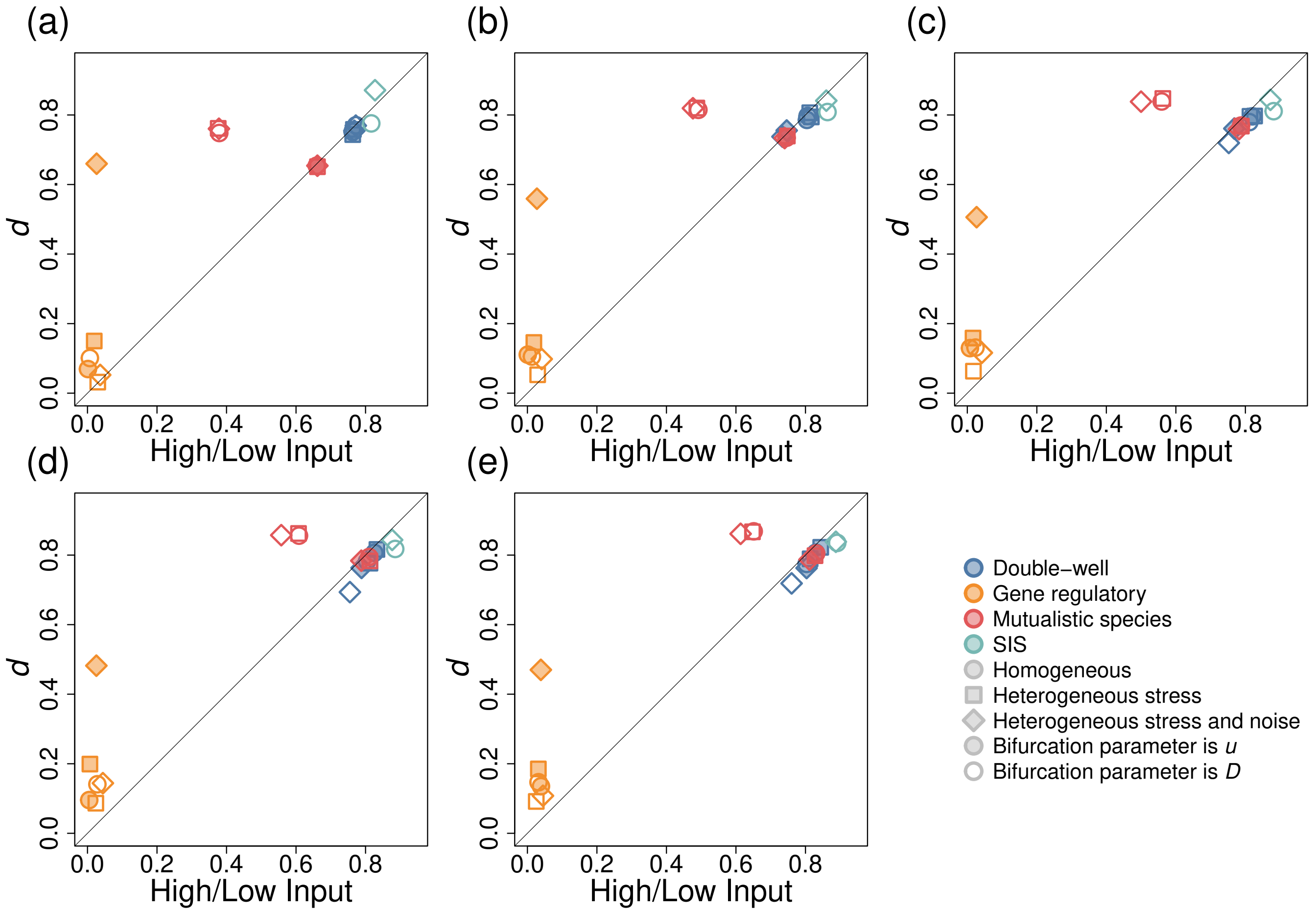

Figure S44: Relationships between the Kendall's τ values for the maximizer of d and those for High/Low Input on the freshwater stream food web network. (a) $n = 1$. (b) $n = 2$. (c) $n = 3$. (d) $n = 4$. (e) $n = 5$.

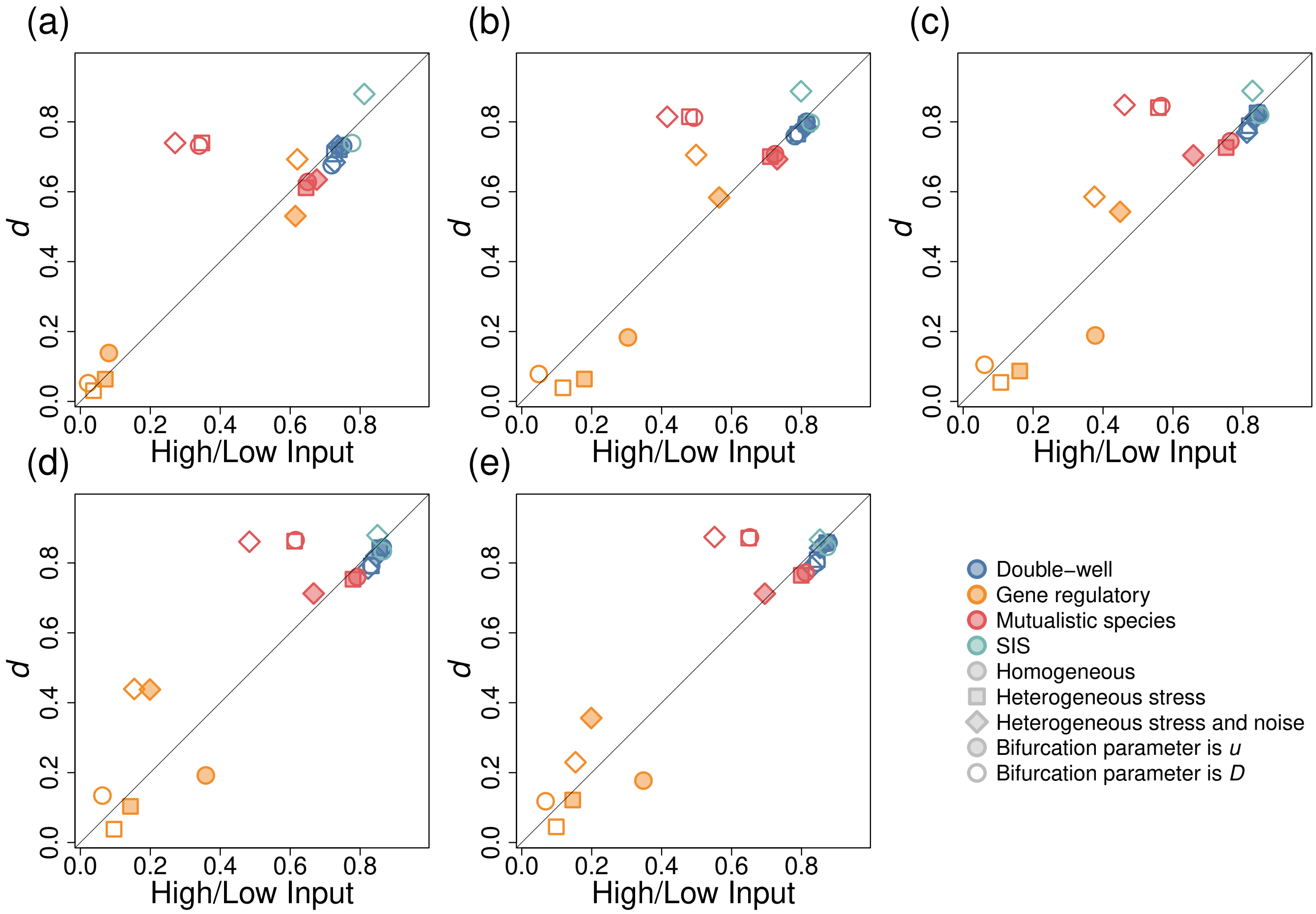

Figure S45: Relationships between the Kendall's τ values for the maximizer of d and those for High/Low Input on the dolphin social network. (a) $n = 1$. (b) $n = 2$. (c) $n = 3$. (d) $n = 4$. (e) $n = 5$.

Supplementary References

[1] T. P. Speed. John W. Tukey's contributions to analysis of variance. *Ann. Stat.*, 30:1649–1665, 2002.

[2] E. Cho and M. J. Cho. Variance of the with-replacement sample variance. In *JSM Proceedings, Survey Research Methods Section*, pages 1291–1293. American Statistical Association, Alexandria, VA, 2008.

[3] G. Vegas-Sánchez-Ferrero, S. Aja-Fernández, M. Martín-Fernández, and C. Palencia. A direct calculation of moments of the sample variance. *Math. Comput. Simul.*, 82:790–804, 2012.

[4] I. Karatzas and S. E. Shreve. *Brownian Motion and Stochastic Calculus*. Springer Science+Business Media Inc., NY, USA, second edition, 1991.

[5] P. Vatiwutipong and N. Phewchean. Alternative way to derive the distribution of the multivariate Ornstein–Uhlenbeck process. *Adv. Diff. Equ.*, 2019:276, 2019.

[6] C. Boettiger, N. Ross, and A. Hastings. Early warning signals: The charted and uncharted territories. *Theor. Ecol.*, 6:255–264, 2013.

[7] N. G. MacLaren, P. Kundu, and N. Masuda. Early warnings for multi-stage transitions in dynamics on networks. *J. R. Soc. Interface*, 20:20220743, 2023.
